# Required duration of mass ivermectin treatment for onchocerciasis elimination in Africa: a comparative modelling analysis

**DOI:** 10.1186/s13071-015-1159-9

**Published:** 2015-10-22

**Authors:** Wilma A. Stolk, Martin Walker, Luc E. Coffeng, María-Gloria Basáñez, Sake J. de Vlas

**Affiliations:** Department of Public Health, Erasmus MC, University Medical Center Rotterdam, Rotterdam, The Netherlands; London Centre for Neglected Tropical Disease Research, Department of Infectious Disease Epidemiology, School of Public Health, Imperial College London, London, UK

**Keywords:** Onchocerciasis, *Onchocerca volvulus*, Africa, Mathematical model, Mass treatment, Ivermectin, Elimination, Prevalence, Breakpoint, Forecasting

## Abstract

**Background:**

The World Health Organization (WHO) has set ambitious targets for the elimination of onchocerciasis by 2020–2025 through mass ivermectin treatment. Two different mathematical models have assessed the feasibility of reaching this goal for different settings and treatment scenarios, namely the individual-based microsimulation model ONCHOSIM and the population-based deterministic model EPIONCHO. In this study, we harmonize some crucial assumptions and compare model predictions on common outputs.

**Methods:**

Using a range of initial endemicity levels and treatment scenarios, we compared the models with respect to the following outcomes: 1) model-predicted trends in microfilarial (mf) prevalence and mean mf intensity during 25 years of (annual or biannual) mass ivermectin treatment; 2) treatment duration needed to bring mf prevalence below a provisional operational threshold for treatment interruption (pOTTIS, i.e. 1.4 %), and 3) treatment duration needed to drive the parasite population to local elimination, even in the absence of further interventions. Local elimination was judged by stochastic fade-out in ONCHOSIM and by reaching transmission breakpoints in EPIONCHO.

**Results:**

ONCHOSIM and EPIONCHO both predicted that in mesoendemic areas the pOTTIS can be reached with annual treatment, but that this strategy may be insufficient in very highly hyperendemic areas or would require prolonged continuation of treatment. For the lower endemicity levels explored, ONCHOSIM predicted that the time needed to reach the pOTTIS is longer than that needed to drive the parasite population to elimination, whereas for the higher endemicity levels the opposite was true. In EPIONCHO, the pOTTIS was reached consistently sooner than the breakpoint.

**Conclusions:**

The operational thresholds proposed by APOC may have to be adjusted to adequately reflect differences in pre-control endemicities. Further comparative modelling work will be conducted to better understand the main causes of differences in model-predicted trends. This is a pre-requisite for guiding elimination programmes in Africa and refining operational criteria for stopping mass treatment.

**Electronic supplementary material:**

The online version of this article (doi:10.1186/s13071-015-1159-9) contains supplementary material, which is available to authorized users.

## Background

Human onchocerciasis, a neglected tropical disease (NTD), is a vector-borne filarial infection caused by *Onchocerca volvulus.* The infection can lead to skin disease, visual impairment and eventually blindness. It occurs primarily in tropical sub-Saharan Africa (99 % of cases), but some foci also exist in Yemen and Latin America. Over the past decades, the overall disease burden of onchocerciasis has been greatly reduced thanks to the implementation of large-scale control programmes, namely, the Onchocerciasis Control Programme in West Africa (OCP, 1974–2002), the African Programme for Onchocerciasis Control (APOC, 1995–2015) and the Onchocerciasis Elimination Program for the Americas (OEPA, 1991-present). In the first decade of the OCP, vector control interventions (aimed at the immature stages of the *Simulium* vectors) were used to interrupt transmission, but the current mainstay of control is annual or biannual mass treatment with ivermectin.

OEPA has successfully interrupted transmission in most foci in the Americas through 6- or 3-monthly ivermectin mass treatment [1–6]. Success was also reported in several African foci with annual or biannual ivermectin mass treatment [7, 8] and other areas also seem to move towards elimination [9], although there are also reports of ongoing transmission in spite of prolonged ivermectin mass treatment [10, 11]. In view of this evidence, APOC decided to target elimination where feasible [12]. The World Health Organization (WHO) set ambitious targets for the elimination of onchocerciasis, which is to be achieved by 2015 in the Americas and Yemen, by 2020 in selected African countries, and by 2025 in 80 % of African countries [13, 14]. There is broad international commitment towards these goals, expressed through the adoption of World Health Assembly Resolution on Neglected Tropical Diseases (WHA66.12) and the endorsement of the London Declaration on Neglected Tropical Diseases 2012 by pharmaceutical companies, donors, endemic country governments and non-governmental organizations involved in NTD control [15].

While past successes provide reason for optimism, an important question remains regarding where and when elimination can be achieved, and whether treatment strategies need to be adjusted to achieve the WHO targets. Work is ongoing to estimate when mass treatment can likely be stopped in different countries and sub-national regions. Important factors to consider when estimating elimination prospects include local transmission conditions (e.g. the endemicity level at baseline in the core of the transmission zone, vector competence, contiguity of a transmission zone), the start year of treatment, treatment frequency, achieved treatment coverage levels and compliance patterns, and complicating factors such as *Loa loa* co-endemicity, the occurrence of suboptimal responses, or lack of infrastructure [16–18]. All these factors to some extent influence the duration of mass treatment required to achieve elimination.

Mathematical models of onchocerciasis transmission and control provide useful tools with which to estimate the required duration of mass treatment in different settings. Two different models have been used to estimate the required duration for various endemic settings and treatment scenarios: the individual-based microsimulation model, ONCHOSIM [19, 20] and the population-based deterministic model EPIONCHO [21–23]. Both models have predicted that the required duration increases with higher baseline endemicity and lower treatment coverage, and can be shortened by about 30–40 % when treating biannually instead of annually. Estimates of the required duration in absolute terms have been more difficult to compare due to a lack of harmonization of model assumptions, simulated scenarios, and presentation of types of output.

In this paper, we present a comparative modelling study to explore the level of agreement between the ONCHOSIM and EPIONCHO models in their projections of estimated programme duration to achieve elimination. A set of policy-relevant scenarios was simulated with both models, after harmonizing a number of critical input parameters. Congruent and disparate results are discussed to understand factors contributing to similarities and divergences. We also pinpoint areas where our knowledge base about the parasite population biology and drug activity is insufficient and further research is needed.

## Methods

### Mathematical models

ONCHOSIM and EPIONCHO, which were developed independently, have been applied in several previous modelling studies (ONCHOSIM [19, 20, 24–26]; EPIONCHO [21–23, 27–29]). A comparison of key features and key model parameters is presented in Table 1 and Table 2. There are many similarities, but the models also differ in some important aspects, e.g. on the extent to which heterogeneities in the human population (e.g. in exposure to blackfly bites) and density dependencies in various processes are captured (e.g. in parasite establishment rate within humans and excess mortality of infected flies). The sections below provide a brief description of the models and their main characteristics. A detailed comparison of the two models and previously published predictions will also be presented elsewhere (Basáñez et al: River blindness: mathematical models for control and elimination, unpublished results).

**Table 1:** Overview of the main characteristics of the ONCHOSIM and EPIONCHO models

Characteristics	ONCHOSIM	EPIONCHO
Basic model structure		
Number and type of spatial locations modelled	Single place	Single place
Population-based or individual-based	Individual-based regarding humans and worms	Population-based
Way of representing infection in hosts	Presence and density at individual level	Mean density in population subgroups (e.g. age, sex, treatment compliance group). Prevalence as a function of mean density assuming an underlying negative binomial distribution
Role of chance	Stochastic	Deterministic
Interventions considered in previous publications	Mass treatment, selective treatment (test and treat), vector control,	Mass treatment, vector control
Features included in the model		
Human population demographics	Birth and death rate dynamically modelled; age and sex composition	Birth and death rate, age and sex composition
Heterogeneities in the human population	Age, sex, life expectancy, level of exposure to blackflies, compliance with MDA, efficacy of treatment	Age, sex, life expectancy, level of exposure to blackflies, compliance with MDA
Blackfly population density	Fixed input as annual biting rate (ABR); seasonal monthly biting rates	Fixed input as ABR; seasonality in biting rates can be included
Exposure to blackfly vectors	Heterogeneous (dependent on age, sex, personal attractiveness to blackflies)	Heterogeneous (dependent on age and sex)
Uptake of infection by blackfly vectors	Varying non-linearly (density-dependent) with infection intensity in human hosts	Varying non-linearly (density-dependent) with infection intensity in human hosts
Infection in blackfly vectors	Density (average L3 load per fly)	Density (average L3 load per fly)
Excess mortality of infected flies	No	Yes
Parasite acquisition in humans	Proportional to mean number of L3 larvae inoculated, denoted by the success ratio	Non-linearly (density-dependent) related to rate of exposure to L3 larvae
Infection in humans	Density (immature or mature worms, mf per skin snip)	Density (non-fertile and fertile worms, mf per mg of skin)
Diagnostic outcomes	Mf count sampling to relate model predictions to data	Sampling process and diagnostic performance of skin snipping not yet included

**Table 2:** Parameter assumptions used for the comparisons presented in this paper

Assumption	ONCHOSIM	EPIONCHO
Life expectancy of adult worms	10 years [38]	10 years [38]
Life expectancy of microfilariae	0.75 years [58]	1.25 years [59]
Distribution of worm survival times	Weibull	Exponential
Proportion of blood meals taken by vectors on humans	0.96 [30] and expert opinion	0.96 (matched to ONCHOSIM)^a^
Macrofilaricidal effect of ivermectin	Not included	Not included
Microfilaricidal effect of ivermectin	100 %, instantaneous upon administration [36]	98-99 % at 2 mo. post-treatment following [40]
Embryostatic effect of ivermectin	All female worms temporarily stop producing mf but resume production gradually, reaching maximum production capacity 11 months post-treatment on average [36]	Fertile worms exposed to ivermectin decrease their mf production according to the dynamics presented in [40] and would fully recover if further untreated
Cumulative effect on mf production by adult worms	35 % reduction in the rate of mf production per dose, on average [36].	35 % reduction in the rate of mf production per dose [36]^a^

^a^Different values were applied in previous publications, but for the current model comparison presented in this paper the assumptions were harmonized with those in ONCHOSIM

#### ONCHOSIM

##### Model background

ONCHOSIM is an individual-based model for simulating onchocerciasis transmission and control in a dynamic human population, based on the technique of stochastic microsimulation [30]. The underlying generalised modelling framework has formed the basis for similar models for other helminthic diseases, including lymphatic filariasis [31], schistosomiasis [32] and soil-transmitted helminthiases (presented elsewhere in this collection [33]).

The model simulates a dynamic human population, consisting of a discrete number of individuals. The population composition changes over time due to birth, aging and death of individuals. Through exposure to bites of *Simulium damnosum* vectors, humans are populated by worms and microfilariae (mf); transmission of infection between human individuals is simulated by means of one central population of blackflies. The fly density is expressed in terms of the average number of fly bites received per (adult) man per year, which is assumed to be constant over time with fixed seasonal variation during the year. At each fly bite, infection may be transferred from human to fly and vice versa. The model considers a non-linear relationship between the mf intensity in human skin (microfilaridermia) and the average number of infective stage (L3) larvae that will develop, from L1 larvae, in flies after taking a blood meal. The biting rate varies between individuals, both randomly and as a function of host age and sex. Therefore, the rate of acquisition of new, incoming worms and the intensity of infection vary between individuals. The relative contribution of different individuals to infection levels in the blackfly population varies in exactly the same way. Only a small, random proportion of the L3 larvae that are released during a bite will develop successfully into an adult worm, defined by a parameter named as the success ratio.

Before introducing an intervention in the simulation, a burn-in period is included to allow infection levels to reach a dynamic, endemic equilibrium. The equilibrium infection levels can be adjusted by modifying assumptions on the average biting rate and, if opportune, exposure heterogeneity among individuals. Mass ivermectin treatment programmes are simulated by specifying the timing of treatment and the therapeutic coverage (i.e. the proportion of the total population taking treatment). The probability that a simulated individual participates in mass treatment with ivermectin is governed by age and sex (children under five years of age are not treated; a random proportion of women of reproductive age is not treated, assuming that they are pregnant or lactating), and a lifelong compliance factor (the higher the factor, the higher the probability that an individual participates in any given treatment round). Furthermore, some individuals never participate in treatment, because they are chronically ill or because they may refuse treatment (these individuals comprise the systematic non-compliers, 5 % of the population in this study). Regarding ivermectin efficacy, we assume the same working mechanism as in previous simulation studies [19, 24, 34]. Drug effects include a microfilaricidal effect, a temporal embryostatic effect, and an anti-macrofilarial cumulative effect that reduces mf production by adult female worms with each treatment dose. In this paper, we adopt a set of assumptions about ivermectin efficacy from a recent publication [20] (termed “assumption set 1” in the cited paper), which has been shown to fit well to trends in skin mf levels as observed in a community trial encompassing five consecutive annual ivermectin treatments in Ghana [35, 36]. According to this set of assumptions: i) the microfilaricidal efficacy of ivermectin is 100 % and it acts instantaneously upon administration; ii) there is no macrofilaricidal effect; iii) the embryostatic effect causes all female worms to temporarily cease mf production, which then recovers gradually over time and reaches maximum production capacity after an average of 11 months; iv) the cumulative effect on female worm fertility amounts to an average 35 % reduction per treatment, with cumulative effects in worms repeatedly exposed to ivermectin.

ONCHOSIM has been previously used to successfully mimic observed longitudinal epidemiological data from various locations [35–38], and has been used for policy making in the West-African Onchocerciasis Control Programme [19, 34]. Further, ONCHOSIM predictions fit reasonably well to longitudinal data from villages along the Gambia and Bakoye River basins in West Africa [20], where 15 to 17 years of annual and/or biannual ivermectin mass treatment have led to elimination of onchocerciasis [7, 8].

More information is provided in the additional files. Additional file 1 provides a formal mathematical description of the model, instructions on installing and running the model, a complete overview of the probability distributions, functional relationships, and parameter values that are used for this study, and annotated input and output files. Additional file 2 contains a zip file, which includes the computer simulation program itself (with the JAVA program code embedded in it), batch files used to run the model, PDF documentation of the XML input, and example input and output files.

##### Model outputs

ONCHOSIM keeps track of changes over time in the infection status (number of immature and mature, male and female worms, and mf density per skin snip) in human individuals, and of the mean infective load in the blackfly populations. Output is obtained by simulating an epidemiological survey, in which mf intensity is measured for each individual as the mean mf count per skin snip (ss), assuming that two snips are taken of about 2 mg each. Measurement variation in mf counts is considered (described by a Poisson distribution around the true mf density) and mf counts may sometimes be false negative (with the probability of false negatives decreasing with higher mf loads). Individual outputs are aggregated to obtain information on the mf prevalence (proportion of all individuals with a positive mf count in either of the two snips), arithmetic mean of individuals’ mf counts per snip (per individual calculated as the mean of two skin snips), and the geometric mean (calculated as exp [(Σ log (*x* +1))/n] - 1, with *x* being the an individual’s mean mf count per skin snip (as above) and *n* the number of individuals included). These outputs are provided for the population as a whole and stratified by age group and sex. In this paper, we always present the mf prevalence in the population aged 5 years and above. The community microfilarial load (CMFL) is equal to the geometric mean mf load per snip in adults aged ≥ 20 years [39].

#### EPIONCHO

##### Model background

EPIONCHO is a deterministic onchocerciasis transmission model that describes the rate of change with respect to time and host age (in both sexes) of the mean number of fertile and non-fertile female adult worms per host, the mean number of mf per milligram (mg) of skin, and the mean number of L3 larvae per simuliid fly. Full mathematical details of EPIONCHO can be found in Turner et al. [21] and Basáñez et al: River blindness: mathematical models for control and elimination, unpublished results. Briefly, the model is based on a prototype presented by Basáñez and Boussinesq [27], extended to include age and sex structure of the host population [28]; the population-level effects of a single [40] and multiple treatments with ivermectin, and increased programmatic realism related to patterns of treatment coverage and systematic non-compliance [21]. Aligning with ONCHOSIM and in accordance with empirical data [41], we have assumed that 5 % of the population is systematically non-compliant with treatment.

The human demography reflects that of savannah areas of northern Cameroon, where the prevailing *O. volvulus–Simulium damnosum sensu lato* combinations (i.e. savannah parasites–*S. damnosum sensu stricto* / *S. sirbanum*) are responsible for the most severe sequelae of onchocerciasis. The age distribution is assumed stationary and the population closed (i.e. no migration). The model captures age- and sex-specific host exposure to blackfly bites, reproducing observed pre-control age-mf (intensity) profiles in Cameroon; patterns also reported in forest areas of Cameroon [42] and elsewhere in former OCP areas of West Africa [39]. EPIONCHO reflects pre-control infection levels in a range of hypo-, meso-, hyper- and highly hyperendemic onchocerciasis foci by varying the annual biting rate (ABR, number of bites received per person per year) of the simuliid vectors.

##### Model outputs

The natural output of EPIONCHO is the per host mean number of mf per mg of skin. Microfilarial prevalence is determined by assuming a negative binomial distribution of mf among hosts with overdispersion parameter treated as a non-linear (hyperbolic) function of the (modelled) mean [43], and fitted to (pre-control) data on the prevalence and intensity of microfilaridermia in Cameroon [27]. In these data, the prevalence and intensity of microfilaridermia were measured by counting mf in two skin snips per person (from the right and left iliac crests), after 24 h incubation in saline. By assuming that this parameterization holds in all population age groups, EPIONCHO estimates: (a) mf prevalence in children aged ≥ 5 years and (b) by Monte Carlo simulation, and using an average weight of 1.7 mg per skin snip [44], the community microfilarial load (CMFL, the geometric mean intensity of mf *per skin snip* in people aged ≥ 20 years.

Additional files 3, 4 and 5 provide instructions for installing and running EPIONCHO, and the source C code (EPIONCHO.c) and R script (EPIONCHO.R) needed to run the simulations presented in this paper.

### Design of the model comparison study

#### Simulated scenarios

In this paper, we present a comparative modelling study to explore the level of agreement between the ONCHOSIM and EPIONCHO models regarding three different outcomes. This was done for a range of pre-control endemicity levels, varying from mesoendemic to very highly hyperendemic or holoendemic (mf prevalence in the population aged ≥5 years ranging from 51 % to 91 %). Treatment scenarios varied with respect to the achieved treatment coverage (50 %, 65 % or 80 %) and treatment frequency (annual, biannual). An overview of all scenarios is provided in Table 3. By tuning the assumed biting rates, both models were calibrated to the predefined levels of mf prevalence in the population aged ≥5 years (as this is the population group that typically participates in epidemiological surveys). For ONCHOSIM, the epidemiological settings are matched to the settings considered by Coffeng et al. [20], where the inter-individual variation in exposure to blackfly bites was low (see also Table 4 below). In this paper, we provide additional model output for the same simulated scenarios. EPIONCHO matched the pre-control levels of mf prevalence, whereas the assumed annual biting rates (partly influenced by the assumed proportion of human blood meals taken by the vectors) and the resulting CMFL are not necessarily identical in the two models.

**Table 3:** Setting characteristics and treatment scenarios for simulations

Factors varied in the simulations:	Values considered
Setting characteristics	
Pre-control endemicity (mf prevalence in the population aged ≥ 5 years)^a^	51 %, 62 %, 81 %, 87 %, 91 %
Treatment scenarios (treatment frequency and coverage constant over time)	
Population coverage of mass treatment	Coverage low (50 %), intermediate (65 %), or high (80 %)
Treatment frequency	Annual or biannual
Duration of mass treatment	Up to 25 years

^a^See Table 4 for information regarding the corresponding biting rates and CMFL

**Table 4:** Comparison of ONCHOSIM and EPIONCHO with respect to the annual biting rate and community microfilarial load (CMFL, the geometric mean no. of mf per skin snip in those aged 20 years and above) that correspond to the pre-set value of mf prevalence in the population aged ≥5 years matched by both models

Pre-set value of mf prevalence in the 5+ population	ONCHOSIM		EPIONCHO	
	ABR (bites / person / year)	CMFL (mf/ss)	ABR (bites / person / year)	CMFL (mf/ss)
51 %	9,409	5.9	2,250	5.5
62 %	10,150	10.5	3,375	9.8
81 %	14,098	33.6	18,906	30.5
87 %	18,078	56.7	34,219	55.0
91 %	22,212	79.4	46,875	83.6

#### Outcomes on which the models are being compared

In past publications, ONCHOSIM provided predictions of the treatment duration needed to drive the parasite population irreversibly to local elimination as evaluated many years post-treatment, while EPIONCHO focused on the time needed to bring mf prevalence below a critical threshold, measured just before what would be the next treatment round [19–21, 23, 43]. This was chosen to reflect the provisional operational thresholds for treatment interruption and commencement of surveillance proposed by APOC in 2010. We now consider both outcomes, to allow comparison with previous work and to understand how the choice of endpoint influences the required durations. In addition, we will compare the models’ predicted trends in infection indicators (prevalence and intensity of microfilaridermia) during mass ivermectin treatment. This is explained in more detail below.

##### Outcome 1: predicted trends in infection with skin microfilariae during ivermectin mass treatment

We compared the models with respect to their predicted trends in microfilarial infection over time during a 25-year programme of annual mass ivermectin treatment, assuming that 65 % of the total population is treated per round. In particular, we looked at predicted trends in mf prevalence among the population aged ≥5 years and the arithmetic mean mf intensity in the whole population, for each of the five baseline mf prevalence levels considered. The prevalence and intensity of mf were assessed annually at the moments of treatment, just before the scheduled treatment round. The dynamic changes in-between treatment rounds are therefore not visualized. For ONCHOSIM, we performed 150 repeated runs per scenario all with the exact same inputs. After exclusion of runs with extinction of infection during the burn-in period (only at the lowest endemicity level, where this occurs in about 10 % of simulation runs) we calculated the average trend in mf prevalence. For EPIONCHO, in accordance with the deterministic nature of the model, only a single simulation was needed per scenario.

##### Outcome 2: treatment duration needed to achieve a provision operational threshold for treatment interruption

For each baseline mf prevalence and for the different treatment scenarios considered, we determined the minimum duration of mass treatment that would be required to bring the mf prevalence as measured just before what would be the next treatment round below a provisional Operational Threshold for Treatment Interruption followed by Surveillance (pOTTIS), as previously reported and defined in [22]. The pOTTIS is based on the working thresholds proposed by APOC in its conceptual and operational framework for onchocerciasis elimination with ivermectin treatment [12]. These thresholds are defined (by APOC) as an mf prevalence of <5 % in all surveyed villages and <1 % in 90 % of such villages, as well as fewer than 0.5 infective larvae per 1000 examined flies (which, given the probability that – near elimination – infective flies will carry only one L3 larva, translates into 0.05 % infective flies). The APOC criteria involve a dual threshold, to capture distribution of mf prevalence levels in multiple communities in an area. APOC’s first criterion (prevalence <5 % in all surveyed villages) suggests that bringing prevalence below 5 % should be sufficient for achieving elimination. The second criterion may serve to verify that mass treatment was effectively implemented throughout the area: if this 5 % threshold were reached even in the communities closest to breeding sites, then considerably lower levels would be expected in most other communities with less intense transmission. This definition has been rendered compatible with the closed population structure of the two models under comparison by defining a single threshold. Rather than using the upper threshold of 5 %, which is still subject to uncertainty and may lead to misinterpretation of the criteria, we have chosen to use the weighted average of the upper and lower thresholds: when the modelled mf prevalence falls to <1.4 %, measured just before the next treatment round, the pOTTIS has been achieved [22]. The pOTTIS is assumed to refer to the mf prevalence in the population aged ≥5 years rather than in the total population, because children under 5 are generally excluded from field surveys or strongly underrepresented.

To estimate the number of treatment rounds required for achieving the pOTTIS, we simulated the respective treatment scenarios (see below) for a maximum duration of 25 years. Trends in mf prevalence were simulated as described above for outcome 1, with mf prevalence measured at the moments of treatment (either annually or biannually, always just before treatment). Treatment was assumed to be no longer needed if the average mf prevalence dropped below the pOTTIS threshold. The required duration in years is then either the minimum number of annual treatments needed to reach the pOTTIS or the number of biannual treatments multiplied by 0.5.

##### Outcome 3: the treatment duration that is needed to drive the parasite population irreversibly to local elimination

The third outcome considered is the minimum required treatment duration that is needed to drive the parasite population irreversibly to local elimination, as previously done with ONCHOSIM and described by Coffeng et al. [20]. As laid out by the transmission breakpoint theory for dioecious parasite species [45, 46], the prevalence (or intensity) of infection does not need to be reduced exactly to zero for mass treatment to be able to stop. Below some epidemiological threshold, which depends on transmission conditions, the probability that a worm successfully reproduces and brings forth at least one new reproducing worm falls below 1 so that transmission becomes unsustainable and the worm population will gradually disappear for the scenario analysed.

With ONCHOSIM, the required duration of mass treatment was estimated based on the eventual occurrence of elimination in a simulation, 50 years after the last treatment, allowing for stochastic fade-out or natural disappearance. Because many processes simulated in ONCHOSIM involve probabilities, repeated model simulations based on the same assumptions will result in slightly different predictions because of stochastic variation. Therefore, with ONCHOSIM, we estimated the probability of elimination as the fraction of 1000 repeated simulations that result in elimination. Elimination was defined as absence of infection 50 years after the last mass treatment, where infection diagnosis was based on two skin snips per person (assuming that the chance of finding zero mf-positive individuals among all simulated individuals (~400) is negligible during sustainable transmission). As in previous ONCHOSIM publications, the required duration is the minimum number of treatment rounds that result in a probability of elimination of ≥99 %.

Deterministic models sometimes allow analytical exploration of breakpoints, e.g. in the absence of interventions or by applying simplifying assumptions on the dynamical responses elicited by interventions [45]. This is not feasible with relatively more complex models such as EPIONCHO. Therefore, for EPIONCHO we evaluated numerically whether the breakpoint was reached by tracking the parasite population long after cessation of the simulated intervention. The implicit breakpoint and hence required treatment duration to drive the parasite to elimination depend on assumptions concerning the mating probability (the probability that female worms are mated), which in turn is influenced by the worm sex ratio, the sexual system (monogamous or polygamous), and the distribution of adult worms in the host population [47]. For the purposes of this paper we have assumed a balanced sex ratio (1:1), a system of polygamy [48], and a Poisson distribution of adult worms in the human host population (assumed to follow a negative binomial distribution in previous papers), with male and female worms distributed together.

### Availability of data and materials

Data and simulation software (EPIONCHO and ONCHOSIM) are made available or can be reproduced via the additional files included in this paper. See the description of additional files below.

## Results

**Fig. 1:**
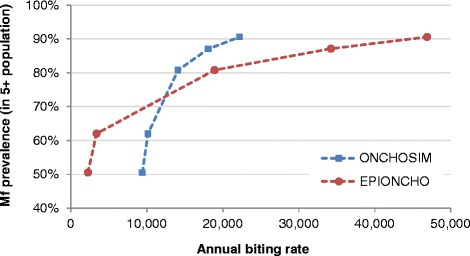
Relationship between the annual biting rate (bites per person per year) and microfilarial (mf) prevalence in the population aged 5 years and above in the two models

The two models were calibrated to match the required pre-control mf prevalence levels in the population aged ≥5 years by adjusting the annual biting rate. Table 4 shows the biting rates that were used as well as the corresponding mf prevalence and CMFL levels. The relationship between annual biting rate and mf prevalence differs somewhat between the models (Fig. 1). The biting rates in ONCHOSIM varied from about 9 to 22 thousand to simulate the required levels of mf prevalence (50–90% in the population aged ≥5 years), whereas in EPIONCHO the biting rates covered a wider range, from about 2 to 47 thousand bites per person per year. The corresponding predicted CMFL values (which were not matched by design) are comparable for the two models (Table 4).

**Fig. 2:**
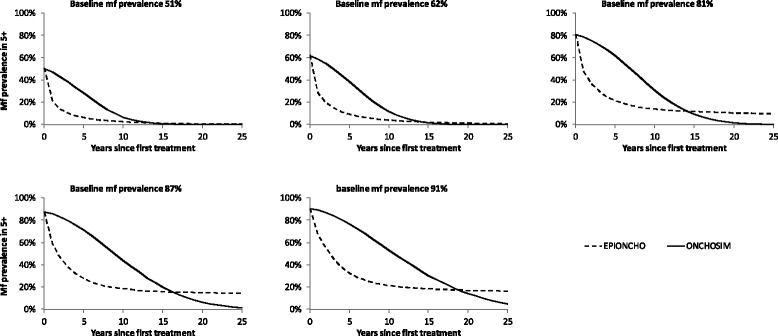
Comparison of expected trends in microfilarial (mf) prevalence during mass treatment, as predicted by ONCHOSIM and EPIONCHO, for settings with different baseline endemicity (mf prevalence in the population aged ≥ 5 years) assuming a coverage of 65 %

**Fig. 3:**
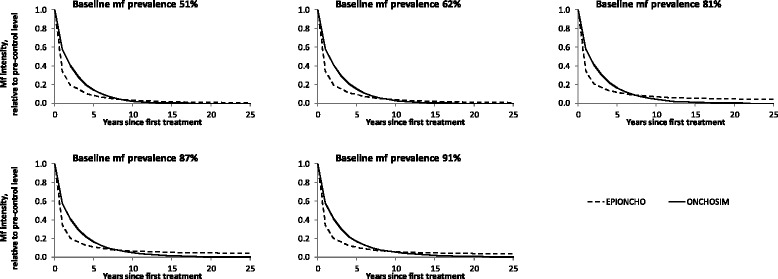
Comparison of expected trends in arithmetic mean mf intensity during mass treatment, as predicted by ONCHOSIM and EPIONCHO, for settings with different baseline endemicity (mf prevalence in the population aged 5 years and above) assuming a coverage of 65 %

Figure 2 compares the predicted trends in mf prevalence in the population aged ≥5 years during a 25-year mass treatment programme where 65 % of the population is treated annually with a single dose of ivermectin. Similarly,

Figure 3 compares predicted trends in the arithmetic mean intensity of mf in the population (all ages) relative to the pre-control (endemic equilibrium) level. EPIONCHO predicts a fast initial decline in both mf prevalence and mean mf count for all 5 endemic settings, but the decline levels off and the two infection indicators tend to move towards a new equilibrium. In ONCHOSIM, the initial decline is less pronounced, but it does not level off as much. Eventually, the infection indicators reach zero faster in ONCHOSIM than in EPIONCHO. The difference between the two models is more pronounced for the mf prevalence than for the mean mf intensity.

**Fig. 4:**
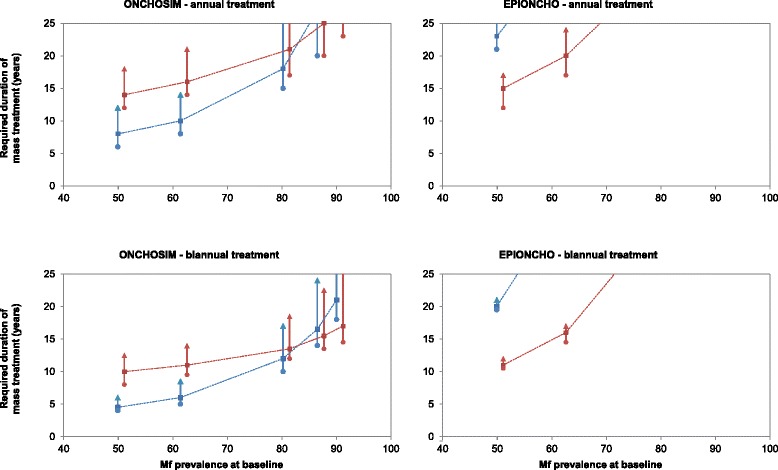
Duration of mass ivermectin treatment in years that is needed to bring mf prevalence below the pOTTIS (red lines and symbols) or to eventually reach local elimination (blue lines and symbols), for ONCHOSIM (left) and EPIONCHO (right) and for annual (top) and biannual treatment (bottom). Dashed lines in each graph connect estimates obtained for different endemicity levels under the assumption that 65 % of the total population is treated per round (coverage). The vertical bars indicate how the duration would change if the coverage was 50 % per round (triangles) or 80 % (circles). To be able to differentiate the prediction intervals obtained for the different endpoints, the results are displayed slightly to the left or right of the actual simulated baseline prevalence (+/− 0.6 %)

Table 5 summarises for both models the estimated required durations to achieve the pOTTIS and to drive the parasite population to local elimination for all settings and treatment scenarios. The same data are graphically represented in Fig. 4 to visualize the patterns in the results. The EPIONCHO- and ONCHOSIM-predicted treatment durations for reaching the pOTTIS are pretty close for settings with moderate baseline prevalence (51 or 62 % mf prevalence). Yet, EPIONCHO predicts a greater lengthening in required treatment duration with increasing baseline endemicity than ONCHOSIM; also predictions for areas with higher baseline endemicity levels (≥81 % mf prevalence) are more divergent. ONCHOSIM predicts that pOTTIS can still be reached by 20–25 rounds of annual mass treatment, if coverage is high enough (80 % required in the highest transmission settings) and that the required treatment duration can be reduced by ~35 % if mass treatment is provided biannually. EPIONCHO is more pessimistic, suggesting that the pOTTIS cannot be achieved in settings with baseline mf prevalence of 81 % or higher, not even with 25 years of biannual treatment and 80 % coverage.

**Table 5:** Comparison of ONCHOSIM and EPIONCHO with respect to estimated duration of treatment that is needed to bring mf prevalence below the provisional operational threshold for treatment interruption followed by commencement of surveillance (pOTTIS) of 1.4 %, measured just before what would be the next treatment round, and the estimated duration of treatment needed to drive the parasite population to local elimination in the absence of further treatment (allowing for the slow natural extinction in the absence of further interventions)

Approximate initial mf prevalence (%) in the population aged ≥ 5 years	Coverage (%)	Treatment duration needed to bring the 12-month or 6-month post-treatment mf prevalence below pOTTIS (years)		Treatment duration needed to drive the parasite population irreversibly to extinction in the absence of further treatment (years)	
		ONCHOSIM	EPIONCHO	ONCHOSIM	EPIONCHO
Annual treatment					
51	50	18	17	12	>25
	65	14	15	8	23
	80	12	12	6	21
62	50	21	24	14	>25
	65	16	20	10	>25
	80	14	17	8	>25
81	50	>25	>25	>25	>25
	65	21	>25	18	>25
	80	17	>25	15	>25
87	50	>25	>25	>25	>25
	65	25	>25	>25	>25
	80	20	>25	20	>25
91	50	>25	>25	>25	>25
	65	>25	>25	>25	>25
	80	23	>25	>25	>25
Biannual treatment					
51	50	12.5	12	6	21
	65	10	11	4.5	20
	80	8	10	4	19.5
62	50	14	17	8.5	>25
	65	11	16	6	>25
	80	9.5	10	5	>25
81	50	18.5	>25	17	>25
	65	13.5	>25	12	>25
	80	12	>25	10	>25
87	50	22.5	>25	24	>25
	65	15.5	>25	16.5	>25
	80	13.5	>25	14	>25
91	50	>25	>25	>25	>25
	65	17	>25	21	>25
	80	14.5	>25	18	>25

Results are shown for different settings, varying with respect to the pre-control mf prevalence in the population aged ≥ 5 years, and for several treatment scenarios, varying with respect to the treatment frequency and achieved coverage (defined as the percentage of people who receive treatment in the total population)

EPIONCHO is also more pessimistic than ONCHOSIM about the possibility of driving the parasite population to local elimination. EPIONCHO suggests that this will only be achievable within 25 years for the setting with 51 % baseline mf prevalence, and that this would require longer continuation of mass treatment than required to achieve the pOTTIS. ONCHOSIM suggests that local extinction is achievable everywhere, although in settings with very high baseline endemicity this might require biannual treatment and/or high treatment coverage (80 %). For areas with moderate baseline endemicity (51 % or 62% mf prevalence), ONCHOSIM suggests that the required treatment duration for driving the parasite population to local elimination is shorter than that needed for achieving the pOTTIS. The reverse was found in settings with the highest baseline mf prevalence.

## Discussion

This paper presents for the first time a vis-à-vis comparison of the ONCHOSIM and EPIONCHO models. We found that whilst EPIONCHO predicts a faster initial decline in mf prevalence and intensity than ONCHOSIM, EPIONCHO is more pessimistic about the long-term prospects of achieving the pOTTIS and local elimination.

### Harmonized input assumptions

For the purpose of the presented comparisons, we harmonized some key assumptions which have previously been identified as very influential on the duration of ivermectin MDA programmes [21]. One critical assumption is the magnitude and irreversibility of the effect of ivermectin on fertility (production of live mf) by adult *O. volvulus* females. By fitting ONCHOSIM to data on mf loads obtained during an early community trial of annual ivermectin treatment in Asubende, Ghana [35], Plaisier et al. [36] had estimated a loss of mf production ranging from 22 to 40% per treatment round. A value of 35 % was recently used in ONCHOSIM by Coffeng et al. [20], but a more conservative value of 7 % (varied in a sensitivity analysis from 1 to 30 %) had been used in EPIONCHO by Turner et al. [22]. In this paper we have used the value of 35 %, which has yielded a good qualitative match for both models to the longitudinal parasitological data on mf loads from the feasibility of elimination study conducted by Diawara et al. [7] in some foci of Mali and Senegal [20].

A previous modelling study by Bottomley et al. [49]—who fitted a model to data from a community trial of biannual ivermectin treatment in Guatemala [44]—had reached the conclusion that the effect of repeated ivermectin treatments on mf production by adult worms was not cumulative. Other studies, e.g. [50, 51], have reported that repeated ivermectin doses may have deleterious effects on adult worms, but the mechanisms and magnitude of such effects remain poorly understood. Model predictions on required treatment duration are also highly sensitive to this parameter, and both models therefore assumed a cumulative effect. It remains, however, critical to better understand the impact of ivermectin on the survival and reproduction (the components of fitness) of *O. volvulus*, to improve our ability to accurately project the outcome of interventions and to appreciate the potential evolutionary implications of such interventions (e.g. selection pressure due to treatment [16]).

The fraction of bites that a blackfly takes on humans (assumed to be 0.96) is also a key parameter. By aligning it between the two models, we brought together the annual biting rates necessary to reproduce initial mf prevalence values (Fig. 1). However, field studies on blood host choice by onchocerciasis vectors [52] have indicated that the human blood index may be variable among component species of the *S. damnosum* s.l. complex, and this information remains important when modelling transmission in different epidemiological settings across Africa, in particular to get an accurate reflection of biting rates needed to produce different infection endemicity levels.

We also harmonized assumptions on the proportion of the population that is systematically non-compliant with treatment, a common parameter in both models. This was done, because a core group of individuals who are untreated and remain infected, potentially provides a source of onward transmission in the human host population, as was also indicated by epidemiological observations of lymphatic filariasis in Haiti, where continuing transmission was related to rates of systematic noncompliance [53]. Harmonization of assumptions on systematic non-compliance does not make the models completely comparable; differences remain in the distribution of treatments over the remainder of the population because of the different approaches to modelling compliance patterns. We need to understand better how treatment compliance patterns can best be modelled. More programmatic data on patterns of individual compliance to inform the mathematical constructs used to model compliance are therefore essential [54].

### Predicted trends in infection during mass treatment

In spite of harmonized treatment efficacy assumptions, EPIONCHO predicted a faster initial decline in mean mf intensity and mf prevalence than ONCHOSIM. In the longer term, ONCHOSIM predicts that infection intensity will decline to zero everywhere, while EPIONCHO suggests that mf intensity may stabilizes at a level above zero. The factors contributing to differences in long-term predictions are discussed below. Here we discuss the factors that contribute to differences in the shorter-term predictions.

The differences in the initial decline in mf intensity may be explained by somewhat different assumptions regarding the temporal dynamics of the microfilaricidal effect of ivermectin as well as the rate of mf production by female worms and mf lifespan, leading to different mf repopulation rates in the period between treatments. This, however, does not fully explain the more marked differences in predicted mf prevalence trends. The individual-based model ONCHOSIM always predicts a relatively slow initial decline in prevalence, because treated individuals are expected to remain mf positive for some time, albeit with considerably lower mf loads. This is in line with observations from a study in Ghana, which showed that mf prevalence rapidly bounced back in the interval between treatment rounds, nearly to pre-treatment levels, while the bounce back in mean mf intensity is less pronounced [35]. In EPIONCHO, mf prevalence is indirectly derived from the predicted mean mf load, through a non-linear prevalence–intensity relationship fitted to pre-control data [43]. In this relationship, low mf loads are associated with similarly low mf prevalence levels. The relationship between the two indicators was assumed to remain unchanged during mass treatment, for consistency with previous EPIONCHO publications. This assumption will have to be adjusted in future work, as the mf prevalence-intensity is likely to be altered by mass treatment, due to the direct microfilaricidal effect of treatment and the relatively slow rate of mf repopulation. Quantification of the post-treatment relationship, ideally using parasitological data obtained during MDA programmes, is therefore an imperative area of further investigation for EPIONCHO.

The model-predicted trends in infection prevalence and intensity, as well as corresponding frequency distributions of mf counts, should be compared against epidemiological data on trends in mf prevalence and intensity during mass ivermectin treatment. Such data are available from the previously mentioned 5-year community intervention trial on the impact of ivermectin mass treatment that was carried out in a highly endemic setting in Ghana [35]. ONCHOSIM has been fitted to these data [36], and the validity of EPIONCHO-predicted trends can be tested against the same data. However, models should also be tested with similar data from other endemic settings, covering a range of pre-control endemicity levels.

### Required duration to reach the pOTTIS or to drive the parasite population to local elimination

An important question for ongoing onchocerciasis elimination programmes concerns the required duration of mass treatment. We explored this on the basis of two endpoints, namely 1) the duration of ivermectin mass treatment required to reach a defined threshold of mf prevalence below which treatment can be stopped (the pOTTIS), and 2) the duration required to drive the parasite locally to elimination, even without further interventions. The first reflects operational criteria for deciding when to stop interventions, although the critical threshold remains to be validated. A limitation of the pOTTIS approach is the focal nature of onchocerciasis, whereby communities with ongoing transmission may act as a source of new infections for those communities where the infection has been eliminated. It is noteworthy that neither EPIONCHO nor ONCHOSIM currently capture spatial transmission processes that may couple transmission among geographically distinct foci. Hence, the elimination projections should be interpreted as capturing the likely outcome of interventions undertaken in circumscribed foci with negligible influx of extraneous infections.

Although predicted trends in infection during mass treatment differ between the two models, estimates of the required duration of annual treatment for achieving the pOTTIS were comparable for settings with moderate baseline mf prevalence (51–62 % mf prevalence). The predictions for areas with higher baseline endemicity levels became more pessimistic and divergent. ONCHOSIM suggests that reaching the pOTTIS would often still be feasible, albeit with longer continuation of treatment, higher coverage, or more frequent treatment. EPIONCHO, however, suggests that even 25 years of biannual treatment with 80 % coverage is not sufficient to achieve the pOTTIS. This is reflected in the EPIONCHO-predicted trends in mf intensity and prevalence, which tend to stabilize at a new non-zero equilibrium after long-term mass treatment (Figs. 2 and 3).

ONCHOSIM is also more optimistic than EPIONCHO about the possibility of driving the parasite population to local extinction. EPIONCHO suggests that the parasite can only be driven to elimination in settings with moderate baseline mf prevalence, although this would require longer continuation of treatment than needed to achieve the pOTTIS. ONCHOSIM suggests that the parasite population would be driven to elimination even before the pOTTIS is reached in settings with moderate baseline endemicity; elimination can also be achieved in settings with higher baseline mf prevalence, although treatment will have to be continued longer than needed for achieving the pOTTIS. This suggests that the fixed operational elimination thresholds proposed by APOC may overestimate the required duration for elimination in the former settings, but underestimate it in the latter.

Long-term predictions on the time needed to reach the pOTTIS or drive the parasite population to local elimination should be interpreted with caution for both models. It will be difficult to validate the models’ predictions regarding the time needed to drive the parasite locally to elimination. Yet, empirical data may help to validate predicted durations for reaching the pOTTIS. In this respect, useful data are available from a study performed in Mali and Senegal, which provided the first evidence that onchocerciasis can be eliminated in Africa through ivermectin mass treatment [7, 8]. Baseline endemicity levels of these regions reflect the lower range of values considered in this study. Data from epidemiological monitoring of ongoing elimination programmes in Africa (such as [9]) will also be informative, in particular if baseline data are available and the area is highlyendemic. Whether or not elimination will really be feasible in very highly endemic areas, with either annual or biannual treatment, remains an important question.

### Possible explanations for differences in required durations for elimination

Several factors contribute to the longer treatment duration required for achieving elimination in EPIONCHO compared to ONCHOSIM, in spite of the faster initial drop in mf prevalence and to a lesser extent intensity. Firstly, EPIONCHO does not account for the possibility of chance elimination of the parasite population (stochastic fade-out), which becomes increasingly likely at very low intensities of infection, especially for small settings (villages) with a couple of hundred inhabitants (as assumed by ONCHOSIM). Secondly, the models differ with respect to assumptions about density dependence in the various processes involved in transmission dynamics (as indicated in Table 1), which may also be important for elimination prospects [45, 55]. In particular, EPIONCHO includes a (negative) density-dependent relationship between the annual transmission potential and the parasite establishment rate; ONCHOSIM does not capture this mechanism, which makes the model more optimistic. Thirdly, the assumed distribution of adult worm and microfilarial survival times and assumptions regarding mf productivity in relation to worm-age may play a role. EPIONCHO assumes an exponential distribution of worm survival times with a long right tail, implying that worm mortality rates are independent of worm age (an implicit assumption of the exponential model). ONCHOSIM assumes a Weibull distribution [38], a more symmetrical distribution with the same mean survival time but a shorter right tail, implying age-dependency of worm-mortality rates. Therefore, it takes considerably longer for the parasite population to die out naturally in EPIONCHO than in ONCHOSIM. In addition to this, ONCHOSIM assumes that the mf production rate declines in older worms, so that the relatively old worm population remaining after long-term ivermectin mass treatment has a relatively low mf production. Such a process is not considered by EPIONCHO. Lastly, the distribution of adult worms among the human population will play a role again through its influence on the mating probability. This assumed distribution is explicit in EPIONCHO (in this paper by using a Poisson distribution) and implicit in ONCHOSIM, driven by between-host heterogeneities in exposure and compliance with treatment.

EPIONCHO and ONCHOSIM also differ considerably in their assumptions regarding the life expectancy of microfilariae, being 0.75 years in ONCHOSIM and 1.25 years in EPIONCHO (Table 2). This is unlikely to have a strong influence on the projected programme durations, because (a) the potency of ivermectin against mf is such that their natural life-span becomes much less relevant and (b) the transmission breakpoint (and the chance of stochastic fade-out) is much more influenced by the life span of adult worms that have a life-expectancy an order of magnitude greater than that of mf (about 10 years versus 1 year). Yet, this difference may explain at least partly—and in combination with the different modelled density-dependent population processes—the markedly different shapes in the relationship between the fitted annual biting rate and the pre-set endemic mf prevalence presented in Fig. 1. In EPIONCHO, on account of the longer life expectancy of mf, and the greater parasite establishment rate at low levels of transmission intensity, a lower biting rate is initially required to produce the same prevalence and (approximate) intensity (CMFL, Table 4) of infection as ONCHOSIM. However, for higher endemicities, and due to the action of the density-dependent establishment of adult worms that is modelled in EPIONCHO but not in ONCHOSIM (Table 1), a higher biting rate is required by EPIONCHO to arrive at the same levels of endemic infection prevalence (and intensity) as ONCHOSIM.

Disentangling the relative importance of different assumptions for various outcomes would require in-depth theoretical research, which is beyond the scope of this paper. This can be done through the development and stepwise comparison of structurally different models of increasing complexity and realism, similar to a previous study on HIV elimination models [56]. To understand which level of complexity is required to address policy questions on control and elimination, it would also be useful to consider the predicted frequency distributions of mf among the host population.

## Conclusion

With the eventual aim to improve the predictive accuracy of simulation models for onchocerciasis transmission and control, and shed more light on whether current interventions are on track to achieve the time-bound elimination goals, two modelling groups working from different methodological traditions have joined forces to harmonize their models and examine the level of agreement in their predictions. This paper focused on comparing, contrasting and understanding the similarities and differences in projected elimination outcomes by two independently developed, well-established models for onchocerciasis transmission, ONCHOSIM and EPIONCHO. Predicting eventual achievement of elimination is a challenge in infectious disease modelling, and possibly even more so when it concerns neglected tropical diseases, because of a general lack of long-term empirical data on the outcome of interest and gaps in knowledge on influential key population-biological parameters. This makes cross-validation between models particularly relevant: converging results help to build trust in predictions, while deviations trigger investigation into the causes and re-evaluation of available evidence which helps to improve model quality. Transparency is required and following “good modelling practice” [57] we provide complete access to the models, with the necessary documentation.

For this first model comparison, we have used a limited set of hypothetical scenarios regarding epidemiological features (initial endemicity, mf prevalence, CMFL and vector biting rates), ranging from mesoendemic to holoendemic onchocerciasis. As was to be expected, this revealed several differences in model predictions, in spite of harmonization of some key parameters. We identified several explanations for the differences, which will be further explored to help to understand strengths and weaknesses of the different modelling approaches and to help to reach consensus on predicted timeframes and optimum interventions for the elimination of onchocerciasis in Africa.

Our next steps using EPIONCHO and ONCHOSIM will include testing model-predicted trends with observed trends in infection during mass treatment, elucidating the differences between the pOTTIS and the transmission breakpoints, refining operational guidance to programme managers based on these results, and identifying APOC projects where elimination goals can be achieved with current strategies and where adjusted, alternative, or complementary interventions are required.

## Ethics approval and consent to participate

Not applicable.

## Consent for publication

Not applicable.

## Additional files

Additional file 1:
**A PDF file, providing a formal mathematical description of the model, instructions on installing and running the model, a complete overview of the probability distributions, functional relationships, and parameter values that are used for this study, and annotated input and output files (Documentation ONCHOSIM v2.58Ap9.pdf).** (PDF 1360 kb) Additional file 2:
**A zip-file, which includes the computer simulation program itself (with the JAVA program code embedded in it), batch files used to run the model, PDF documentation of the XML input, and example input and output files.** Instructions on how to run the model are provided in Additional file 1 (ONCHOSIM simulation program.zip). (DOCX 15 kb) Additional file 3:
**A Word document containing instructions for installing and running EPIONCHO (Instructions for installing & running EPIONCHO.docx).** (DOCX 71 kb) Additional file 4:
**Source EPIONCHO code, written in programming language C (EPIONCHO.c).** (C 30 kb) Additional file 5:
**R script needed to run the simulations presented in this paper (EPIONCHO.R).** (R 5 kb)

## Abbreviations

ABR: Annual biting rate
APOC: African Programme for Onchocerciasis Control
CMFL: Community microfilarial load
mf: Microfilariae/microfilarial
mg: Milligram
OEPA: Onchocerciasis Elimination Program for the Americas
OCP: Onchocerciasis Control Programme in West Africa
pOTTIS: Provisional operational thresholds for treatment interruption followed by surveillance
ss: Skin snip
