# Supplementary material for: Required duration of mass ivermectin treatment for onchocerciasis elimination in Africa: a comparative modelling analysis
Source: Parasit Vectors. 2015 Oct 22;8:552. doi: 10.1186/s13071-015-1159-9 (PMC4618738; doi:10.1186/s13071-015-1159-9)
Supplement: Additional file 1: — A PDF file, providing a formal mathematical description of the model, instructions on installing and running the model, a complete overview of the probability distributions, functional relationships, and parameter values that are used for this study, and annotated input and output files (Documentation ONCHOSIM v2.58Ap9.pdf). (PDF 1360 kb) [file 13071_2015_1159_MOESM1_ESM.pdf]

Additional file 1.

The ONCHOSIM simulation model: a disease-specific model variant of the WORMSIM modelling framework.

This document is a supplement to the following manuscript:

> Wilma A. Stolk, Martin Walker, Luc E. Coffeng, María Gloria Basáñez, Sake J. de Vlas. Required duration of mass ivermectin treatment for onchocerciasis elimination in Africa: a comparative modelling analysis. Submitted to Parasites and Vectors (2015)

Contents

1 Introduction 4

1.1 ONCHOSIM: modelling the transmission and control of onchocerciasis 4

1.2 ONCHOSIM as a variant of WORMSIM 4

1.3 This document 4

2 WORMSIM 5

2.1 Modelling approach 5

2.2 Implementation of WORMSIM in software 5

3 Formal description of WORMSIM (v2.58Ap9) 6

3.1 Human demography 6

3.2 Transmission of infection 7

3.2.1 Exposure to infection and acquisition of new worms 7

3.2.2 Within-host dynamics of infection 8

3.2.3 Host contribution of infective material to the cloud 9

3.2.4 Dynamics of infective material in the cloud 10

3.3 Morbidity 10

3.4 Mass treatment coverage and compliance 11

3.5 Parasitological effects of treatment 12

3.6 Vector control 13

3.7 Surveys 13

3.8 Simulation warm-up 14

4 ONCHOSIM input: Probability distributions, functions and parameter values 15

5 Instructions for installing and running WORMSIM 19

6 Annotated input file 22

6.1 Inputfile header 22

6.2 <Simulation> 22

6.3 <Demography> 23

6.4 <Blindness> 24

6.5 <Exposure.and.contribution> 24

6.6 <Immunity> 25

6.7 <Worm> 25

6.8 <Fly> 26

6.9 <Mass.treatment> 26

6.10 <Vector.control> 27

7 Annotated output file 28

8 Reference 30
Appendix I Probability distributions for stochastic processes available in WORMSIM .. 32
Appendix II Functional relationships available in WORMSIM 34

1 Introduction

1.1 ONCHOSIM: modelling the transmission and control of onchocerciasis

ONCHOSIM is a computer program for modelling the transmission and control of the tropical parasitic disease onchocerciasis, or river blindness. It was developed in collaboration with the Onchocerciasis Control Programme in West Africa (OCP, 1974-2002), as a tool in the evaluation and planning of control operations. The African Programme for Onchocerciasis Control (1995-2015) also adopted the program as a tool. The model comprises a detailed description of the life history of the parasite *Onchocerea volvulus* and of its transmission from person to person by *Simulium* flies. The effects of different control strategies, based on larvicide application and chemotherapy (ivermectin), on the transmission and on the disease symptoms can be evaluated and predicted. Since its conception, the model has been used for multiple applications as described elsewhere [1-11].

1.2 ONCHOSIM as a variant of WORMSIM

The ONCHOSIM modelling framework for simulating transmission and control was first described by Plaisier et al in 1990 [1]. In subsequent years, Erasmus MC has developed similar models for lymphatic filariasis (LYMFASIM [12]), schistosomiasis (SCHISTOSIM [13]), and most recently also soil-transmitted helminthiasis [14]. The initial models were implemented in three separate, disease-specific computer programs (written in C++), although all had very similar features. Recently, WORMSIM was developed as a generalised framework for modelling transmission and control of helminth infections in humans, and it contains extra features to allow the simulation of these different infections. Through adjustment of input specifications on structural assumptions and the value of model parameters, WORMSIM can be made to represent onchocerciasis or other diseases. We continue to use the name ONCHOSIM to denote the WORMSIM model variant for onchocerciasis, because ONCHOSIM is well known in the field and we want to make clear that the model is still the same despite a different software implementation.

1.3 This document

In section 2, we briefly describe the general properties of the WORMSIM modelling framework, followed by a formal description in section 3. In footnotes we highlight details and alternative options that are not evident from the mathematical descriptions. In section 4, we provide an overview of the probability distributions, functional relationships, and parameter values that are used in ONCHOSIM. In section 5, we provide instructions for installing and running WORMSIM. In sections 6 and 7, we present annotated WORMSIM input and output files, respectively.

2 WORMSIM

2.1 Modelling approach

WORMSIM simulates the life histories of individual helminths and their transmission from person to person mediated by either a cloud of vectors or an environmental reservoir. In addition, WORMSIM can be used to evaluate the effects of different control strategies, such as vector control and chemotherapy. WORMSIM combines two simulation techniques; *stochastic microsimulation* is used to calculate the life events of individual persons and their inhabitant parasites, while the dynamics of infective material in the cloud (i.e. the vector population or environmental reservoir) is simulated *deterministically*.

2.2 Implementation of WORMSIM in software

WORMSIM was programmed in Java using object-oriented principles. Individual people and mature worms are modelled as distinct objects. WORMSIM is event-driven, which means that time progresses as a result of events (although monthly events are used for most processes). The main advantages of the implementation in Java are high code quality and therefore easier maintenance and extension. Model input parameters are specified in a structured XML-file, which is automatically validated using an XML Schema before the start of a set of simulations.

The WORMSIM framework is very flexible in that it allows the user to choose probability distributions for stochastic processes (Appendix I) and functional relationships for deterministic processes (Appendix II), and to change the associated parameter values. Table A1-1 provides an overview of the probability distributions, functional relationships, and parameter values used in this study.

3 Formal description of WORMSIM (v2.58Ap9)

3.1 Human demography

The human population dynamics is governed by birth and death processes. We define *F(a)* as the probability to survive to age a. The cumulative survival for intermediate ages is obtained by linear interpolation.

The expected number of births (per year) at a given moment t is given by:

$$R_b(t) = \sum_{a=1}^{n_a} N_f(a,t) \cdot r_b(a) \qquad (1)$$

with:

$N_f(a,t)$	number of women in age group a at time t
$r_b(a)$	annual birth rate in age-group a.
n_a	number of age-groups considered.

Each month, $R_b(t)$ is adapted according to the number of women and their age-distribution.

Once every year, the total number of human individuals is checked; if the total number is larger than a user-defined value, a fraction (also user-defined) is randomly removed from the simulation.

The population distribution resulting from the aforementioned parameters is illustrated in Figure A1-1. In this example, the simulated population structure closely follows the age distribution in Sub-Saharan Africa as estimated by the UN Population Division for the year 2000 (Figure A1-1) [1].

Figure A1-1. Population demography simulated in WORMSIM in absence of excess mortality due to disease (bars), compared to the 2000 population for Sub-Saharan Africa (diamonds; UN Population Division, World Population Prospects: The 2012 Revision).

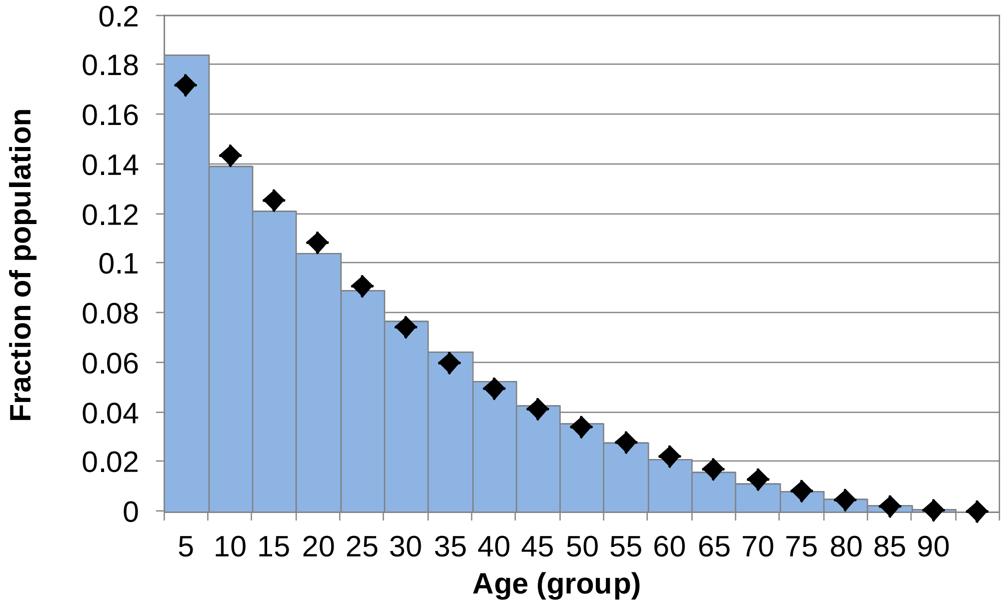

3.2 Transmission of infection

In WORMSIM, transmission between individuals is mediated by a conceptual cloud, which either represents a vector population or an environmental reservoir of infection. Individual human hosts are *exposed* to the infective material in the cloud at varying rates, given their age, sex, and personal factors. Vice versa, individual hosts *contribute* infective material (larvae or eggs) to the cloud, the amount depending on the number and reproductive statues of worms in the individual, as well as an individual host's contribution rate (depending on age, sex, and personal factors). The amount of infective material in the cloud is updated in discrete monthly time steps. Below, we describe how WORMSIM simulates a full transmission cycle: human exposure to infection and acquisition of worms, dynamics of infection within humans, contribution of infective material to the cloud, and within-cloud dynamics of infective material.

3.2.1 Exposure to infection and acquisition of new worms

First, we define the overall force of infection $lr(t)$ acting on the human population in month *t* as a function of the current absolute amount of infective material in the cloud $\overline{lu}(t)$:

$$lr(t) = \overline{lu}(t) \cdot \zeta \cdot v \qquad (2)$$

Here, ζ (zeta) is a scalar representing the overall exposure rate, and *v* is the probability that an infective particle in the reservoir successfully develops into a parasite life stage that is capable of infecting a human host.[a]

Next, we define the force of infection acting upon individual *i* of age *a* and sex *s* as:

$$foi_i(t) = lr(t) \cdot \frac{Ex_i}{\sum_{i=1}^{N(t)} Ex_i} \qquad (3)$$

Here, Ex_i is the relative exposure of an individual, taking into account age *a* and sex *s*, as well as personal factors:

$$Ex_i = Exa(a_i, s_i) \cdot Exi_i \qquad (4)$$

with:

$Exa(a_i, s_i)$	Relative exposure of person with age *a* and sex *s*, defined as a linearly interpolated function of user-defined exposure rates for a finite set of ages (for each sex).
Exi_i	Exposure index of person *i*, which captures personal factors related to e.g. behaviour and occupation. Exi_i is assumed to follow a gamma distribution with mean 1.0 and shape and rate (or 1/scale) equal to α_{Exi}. The exposure index of a person remains constant throughout lifetime.[b]

[a] ζ is perfectly negatively correlated with transmission probability *v*, success ratio *sr*, relative biting rate *rbr*, and vector zoophily *z*. See also the section on contribution of infective material to reservoir. For filarial transmission, we set $\zeta = 1$, quantify *v* based on vector biology, set *sr* to a constant value, and calibrate transmission with *rbr*. For STH, we set $v = rbr = sr = 1$, and calibrate transmission with ζ, which has a more natural explanation in the STH context (exposure to the reservoir) than *rbr*.

[b] If desired, other continuous probability function can be chosen.

Finally, a person i is assumed to become infected in month m, according to a Poisson process with rate equal to $foi_i(t) \cdot sr \cdot Imm_i(t)$. Here, success ratio sr is a constant representing the probability that an inoculated infective particle will develop into a macroparasite.[a] Finally, $Imm_i(t)$ represents the impact of the host's immune response in montht t on incoming infections [2]:

$$Imm_i(t) = 1 - \alpha_{imm} \cdot imm_i \cdot W_{cum,i}(t) \tag{5}$$
$$W_{cum,i}(t) = W_i(t) + \beta_{Imm} \cdot W_{cum,i}(t-1)$$

Here, α_{imm} is the effect of immunity and imm_i is an individual host's capacity to elicit an immune response, drawn from a positive bounded probability distribution with mean one (e.g. a gamma distribution with equal shape and rate (1/scale) parameters). $W_{cum,i}(t)$ is the cumulatively experienced worm burden of host i in month t, $W_i(t)$ is the worm burden of host i in month t, and β_{Imm} represents the immunological memory span ($\beta_{Imm} = e^{-\ln(2)/\lambda_{imm}}$, where λ_{imm} is the desired half-life (in months) of the immunological response; vice vera $\lambda_{imm} = -\ln(2)/\ln(\beta_{Imm})$).

3.2.2 Within-host dynamics of infection

For convenience, in this section we drop the subscript i for individual humans. The lifespan of male and female parasites within human hosts is a random variable: $Tl \sim \text{Weibull}(\mu_{Tl}, \alpha_{Tl})$, with mean μ_{Tl} years and shape α_{Tl}.[c] Once parasites come of age (i.e. when they pass the prepatent age pp), female worms can start producing larvae or eggs, and males can inseminate female worms. The reproductive capacity $r(a,t)$ of a patent female worm of age a at time t is calculated as follows (in absence of drug effects):

$$r(a,t) = R(a - pp) \cdot m(t) \cdot z(t) \tag{6}$$

with:

- $R(A)$ Potential reproductive capacity of a female worm, A years after reaching patency, defined as a linearly interpolated function of user-defined values for a finite set of ages.
- $m(t)$ Mating factor at time t
- $z(t)$ The exponential fecundity coefficient at time t, defined as $z(t) = e^{-W(t)\lambda_z}$, where $W(t)$ is the number of adult worms (males and females) in a given host at time t, and $\lambda_z \in \mathbb{R}^+$ quantifies the amount of negative density dependence. If $\lambda = 0$, there is no exponential saturation in egg production ($z(t) = 1$).

To produce larvae or eggs, a female worm must be inseminated each reproductive cycle rc, defined in terms of months. If insemination took place less than rc months ago, then $m(t) = 1$. Otherwise, the probability of insemination or reinsemination $P_{ins}(t)$ in month t is given by:

---

[c] For readers used to the other commonly used parameterization of the Weibull distribution in terms of shape k and scale λ, shape k is α_{Tl} (as described in this document) and scale $\lambda = \mu_{Tl}/\Gamma(1 + 1/\alpha_{Tl})$.

$$P_{ins}(t) = \begin{cases} pot \cdot W_m(t)/W_f(t) & \text{if} \quad pot \cdot W_m(t) < W_f(t) \\ 1 & \text{if} \quad pot < 0 \;\text{ or otherwise} \end{cases} \tag{7}$$

with:

$W(t)$ the number of male (W_m) or female (W_f) parasite in the human at time t

pot the number of female worms that a male worm can inseminate per month[d]

If no insemination takes place then $m(t) = 0$ and the female worm has a new opportunity to be inseminated in the next month $t + 1$. If insemination occurs in month t_i then $m(t) = 1$ during $t_i \le t < t_i + rc$.

The density of larvae (e.g. per skin snip) or eggs (e.g. per gram faeces) $sl(t)$ in a host at time t is calculated by accumulating the production of all female parasites over the past Tm months within that host:

$$sl(t) = O\big(el(t)\big) \tag{8}$$

$$el(t) = \sum_{j}^{n_i} d_j \sum_{x=1}^{Tm} r_j\big(a_j - x, t - x\big) \tag{9}$$

with:

$el(t)$ the *effective parasite load* at time t. This intermediate variable describes the female parasite load obtained by weighting each worm according to the mf-productivity during the past Tm months.

$O(.)$ A function that returns the total amount of infective material produced by female parasites. For onchocerciasis, we assume that $O(.)$ is a linear function through the origin with slope 7.6 mf per fully productive adult female worm.[e]

d_j *dispersal factor* of female parasite j. This is a random variable (mean 1.0) drawn for every "newborn" worm, and accounts for differences in the contribution of female worms to the density at the standard site of the body where samples are taken or vectors bite.

Tm (fixed) lifespan of larvae or eggs within the host in terms of months.

n_i number of parasites alive during at least one of the months $t\text{-}1,\ldots,t\text{-}Tm$.

3.2.3 Host contribution of infective material to the cloud

Given the density of larvae or eggs $sl_i(t)$ in all $N(t)$ host in month t, the total amount of infective material that is contributed to the cloud by the host is defined as

---

[d] When the user specifies a negative value for *male potential*, female worms can produce larvae or eggs in the absence of male worms.

[e] Alternatively, other functional relationships between *el* and *sl* can be defined. Saturating functionsshould not be used when $Tm > 1$, as this will cause partial saturation of female worm productivity in month t, given the output in months $t - 1$ through $t - Tm$. This will be alleviated in a future version of WORMSIM by setting $sl(t) = \sum_{x=1}^{Tm} O\big(\sum_{j}^{n_i} r_j(a_j - x, t - x)\big)$.

$$\bar{lu}(t)_{in} = \sum_{i=1}^{N(t)} Mbr(t) \cdot rbr \cdot U(sl_i(t)) \cdot Co_i \qquad (10)$$

Here, $Mbr(t)$ is the average contribution rate in month *t* (*monthly biting rate* for filarial infections), allowing the user to define a seasonal pattern (in absence of vector control). The relative biting rate *rbr* is used to scale this seasonal pattern to some desired level.[a] The function $U(.)$ returns the amount of infective material taken up by the cloud given the density of eggs or larvae $sl_i(t)$ in a host, possibly in a density dependent manner to represent e.g. limited vectorial capacity to transmit infection.[f] Last, Co_i is the relative contribution of an individual, given age, sex, and personal factors:

$$Co_i = Coa(a_i, s_i) \cdot Coi_i \qquad (11)$$

with:

$Coa(a_i, s_i)$	Relative contribution of person with age *a* and sex *s*, defined as a linearly interpolated function of user-defined exposure rates for a finite set of ages (for each sex).
Coi_i	Contribution index of person *i*, which captures personal factors related to e.g. behaviour and occupation. Coi_i is assumed to follow a gamma distribution with mean 1.0 and shape and rate (or 1/scale) equal to α_{Coi}. The contribute index of a person remains constant throughout lifetime. In WORMSIM default assumption is that $Coi_i = Exi_i$, unless separate distributions are defined.

3.2.4 Dynamics of infective material in the cloud

For the dynamics of infective material in the cloud we define a deterministic, discrete model:

$$\bar{lu}(t) = \bar{lu}(t)_{in} + \psi \cdot \bar{lu}(t-1) \qquad (12)$$

Each month, new infective material $\bar{lu}(t)_{in}$ is added to the cloud, and a fixed proportion ψ of the infective material from the previous month is carried over, assuming exponential survival of infective material. The average life span of infective material in the cloud is then defined as $-1/\ln(\psi)$ months. To simulate filarial transmission, we set $\psi = 0$, such that the cloud represents a vector population in which larvae survive for much shorter than a month. To simulate hookworm or other STH infections, we set $0 < \psi < 1$, such that the cloud represents an environmental reservoir of infection in which infective material survives for a non-negligible time.

3.3 Morbidity

The event of a person developing symptoms at age *a* depends on the *accumulated parasite load* (elc) of a person:

$$elc(a) = \sum_{x=0}^{a} el(x) \qquad (13)$$

[f] For filarial infection, $U(.)$ typically is a density-dependent function of $sl_i(t)$ to represent limited vectorial capacity to transmit infection, whereas for STH, we take $U(.)$ to be the identity function.

Each person has a threshold level elc (denoted as Elc) at which a person goes blind. Elc follows a probability distribution: $Elc \sim \text{Weibull}(\mu_{Elc}, \alpha_{Elc})$, with mean μ_{Elc} and shape α_{Elc}. Person i goes blind at age a when:

$$elc_i(a) \geq Elc_i > elc_i(a-1) \tag{14}$$

At that moment the remaining lifespan at age a is reduced by a factor *rl* which follows a user-defined distribution on [0,1].[g]

3.4 Mass treatment coverage and compliance

The primary characteristic of a certain ivermectin mass treatment *w* is the coverage C_w (fraction of the population treated). However, a difficulty in calculating individual chances of participation is that there are several exclusion criteria for the drug. Moreover, compliance to treatment differs from person to person. Exclusion criteria can be either permanent (chronic illness) or transient (e.g. related to age or pregnancy). We define the eligible population as the total population *minus* a fraction f_c that is permanently (lifelong) excluded from. The coverage among the eligible population C'_w is now given by:

$$C'_w = C_w/(1 - f_c) \tag{15}$$

Here, C'_w cannot be larger be than one (i.e. is capped off at one).

To capture transient contra-indications and other age- and sex-related factors for participation in mass treatment, we the age- and sex-specific relative compliance $c_r(k,s)$. Note that in $c_r(k,s)$ only the *ratio* between the values for the different groups is relevant.

Now, the coverage $c(k,s,w)$ in each of the age- and sex-groups at treatment round *w* is calculated as:

$$c(k,s,w) = \frac{c_r(k,s) \cdot N(w)}{\sum_{s=1}^{2} \sum_{k=1}^{n_a} c_r(k,s) \cdot N(k,s,w)} \tag{16}$$

with:

$N(k,s,w)$
: Number of individuals eligible to treatment in age-group *k* and sex *s* at treatment round *w*.

$N(w)$
: Total number of eligible individuals at treatment round *w*.

Finally, the probability to participate in treatment round *w* for an eligible person *i* of age-group *k* and sex *s* is given by:

$$Ptr_{i,w} = co_i^{\frac{1-c(k,s,w)}{c(k,s,w)}} \tag{17}$$

with:

co_i
: Personal compliance index. This is considered as a lifelong property and is generated by a uniform distribution on [0,1]

Note that for all *k* and *s* the average value of $Ptr_{i,w}$ equals $c(k,s,w)$. Now, in WORMSIM we define 3 coverage models. In model 0, the probability to be treated is as given in equation

[g] For STH modelling, we do not use this feature, and thus assume zero excess mortality from infection.

(17). In model 1, the probability is equal to $c(k, s, w)$ and the compliance index co_i is ignored. The simplest model is model 2 in which the treatment probability simply equals C'_w. All models take account of a fraction f_c of permanently excluded persons. Figure A1-2 illustrates the impact of different assumptions about compliance patterns on the proportion of the population that has never been treated after a certain number of treatment rounds.

Figure A1-2. Relation between compliance patterns and proportion of population that has never been treated. For simplicity, here we assume that compliance is independent of age and sex. Random compliance (solid line) means that eligible individuals participate completely at random (compliance model 1 or 2 in WORMSIM, depending on whether age and sex-patterns are required). Systematic compliance (dotted line) means that an individual either always participates (if eligible) or never (compliance model 1 or 2 in WORMSIM, combined with a fraction of excluded people equal to one minus the target coverage). The mixed compliance pattern (dashed line) means that some individuals are systematically more likely to participate than others (but everyone will participate at some point; compliance model 0 in WORMSIM).

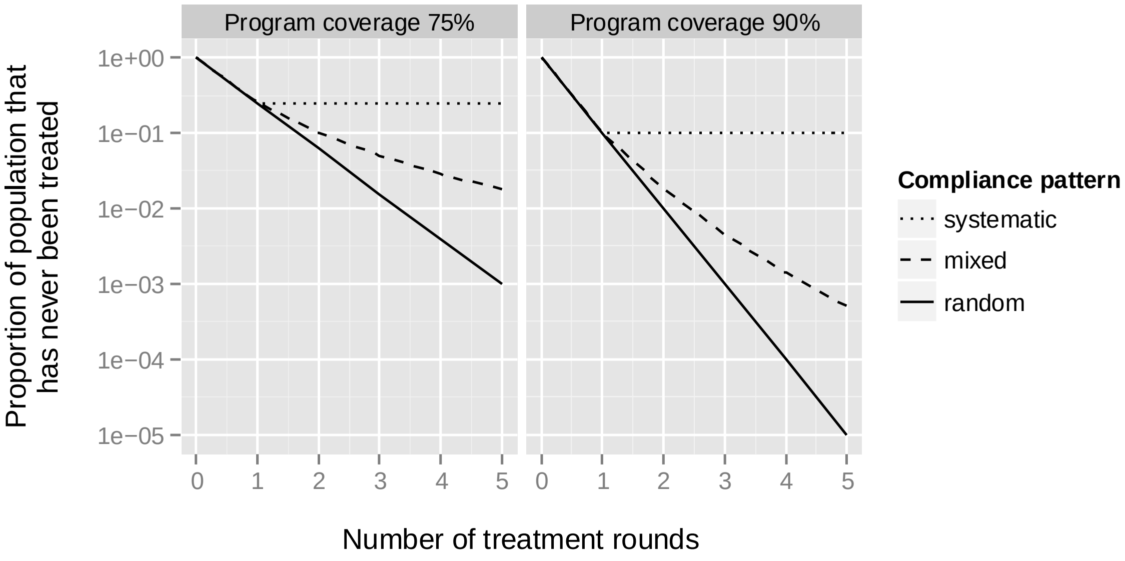

3.5 Parasitological effects of treatment

In WORMSIM, drug treatment affects parasites in three main ways. First, a drug may instantly kill a proportion of larvae or eggs present in a host. This proportion is either fixed or a randomly drawn from a user-defined probability distribution for each host and treatment.

Second, a drug may instantly kill pre-patent and adult worms with probability m_i in host i. A worm j dies when a random variate u_j on [0,1] (redrawn for every new treatment) is smaller than or equal to m_i.

Third, a drug may temporarily and/or permanently (and cumulatively) reduce the reproductive capacity of female worm by a proportion d_i in host i. In case of a temporary effect, the reproductive capacity will restore within a period Tr_i to its maximum value (in case of any concomitant permanent reductions, reproductive capacity will regenerate to the new, permanently reduced maximum value. The second and third effect are jointly defined as follows:

$$m_i = v_i m_0 \tag{18}$$

$$d_i = v_i d_0$$
$$Tr_i = v_i Tr_0$$
$$r_j(a_j,t) = r_j^0(a_j,t) \cdot (1-d_i) \cdot \left(\frac{\tau}{Tr_i}\right)^s, \quad \text{if } u_j > m_i,\ d_i < 1, \text{ and } t < Tr_i$$
$$r_j(a_j,t) = r_j^0(a_j,t) \cdot (1-d_i) \quad \text{if } u_j > m_i,\ d_i < 1, \text{ and } t \geq Tr_i$$
$$r_j(t) = 0 \quad \text{otherwise}$$

with:

- v_i Relative effectiveness of treatment in person i. For every separate treatment and person, a new value is drawn for v_i from a user-defined distribution (i.e. the relative effectiveness applies to all worms in a person during a specific treatment).
- m_0 Average fraction of prepatent and adult parasites killed.
- d_0 Average permanent (unrecoverable) reduction in female reproductive capacity.
- Tr_0 Average duration until full recovery from temporary effects on female reproductive capacity.
- $r_j(a_j,t)$

  Reproductive capacity of female worm j in month t, τ months after the last treatment.
- $r_j^0(a_j,t)$

  Reproductive capacity of female worm j had person i not been treated at the last round, τ months ago.
- s Shape parameter of the recovery function.

In addition to this, we explicitly consider that some persons (a user-defined random fraction of the treated population) do not at all react to the drug during a certain treatment due to malabsorption (e.g. due to vomiting or diarrhoea).

3.6 Vector control

Vector control is modelled as a reduction of the monthly biting rates during a given period of time. A period of vector control[h] is specified as the year + month of the beginning of the strategy and the year + month of the end of a strategy. If a certain month during a period of d days larvicides have been applied, then the reduction in $Mbr(t)$ in that month equals d/30 x 100%.

3.7 Surveys

During the simulation, user-defined surveys will take place. During a survey, for all simulated individuals the actual number of male and female worms is recorded, and a diagnostic test is simulated to detect infective material (larvae, eggs). For the diagnostic test, the expected amount of infective material per sample (e.g. microfilariae per skin snip, or eggs per gram faeces) for an individual is given by $sl_i(t)$.

---

[h] Multiple periods of vector control can be specified, each with its own effectiveness.

The actual number of infective particles (microfilariae, eggs, etc.) in the sample is assumed to follow a discrete distribution like a Poisson or negative binomial distribution, with mean equal to $sl_i(t)$.[i] At each epidemiological survey a user-defined number of samples are taken from all simulated persons, for which the results are averaged (per simulated person). The results of such a survey are post-processed to arrive at age and sex-specific prevalences and intensities of infection.

3.8 Simulation warm-up

In general, before starting simulation of interventions in ONCHOSIM, a 200-year warm-up period is simulated, such as to allow the human and worm population to establish equilibrium levels, given the parameters for average fly biting rate and inter-individual variation in exposure to infection. At the start of the warm-up period, an artificial force of infection is simulated for a user-defined number of years, allowing worms to establish themselves in the human population (here: 4 worms per person per year for 7.5 years). After the 200 warm-up years, the simulated infection levels are no longer correlated with the initial conditions at the start of the warm-up period.

---

[i] For filariasis transmission, we typically assume that sampling error is Poisson distributed.

4 ONCHOSIM input: Probability distributions, functions and parameter values

Table A1-1. WORMSIM quantification used to simulate onchocerciasis transmission. Given that WORMSIM is a general modelling framework that covers various helminthic infections, certain parameters do not apply to onchocerciasis transmission but are listed anyway for the sake of completeness (indicated where applicable).

Parameter	Value	Source
**Human demography**		
Cumulative survival ($F(a)$), by age (see also Figure A1)		[15]
0	1.000	
5	0.804	
10	0.772	
15	0.760	
20	0.740	
30	0.686	
50	0.509	
90	0.000	
Fertility rate per woman ($R(a)$), by age		[15]
0–14	0.000	
15–29	0.109	
30–49	0.300	
50+	0.000	
Population trimming	10% if population size exceeds 440.	Assumption
**Transmission of infection**		
General transmission parameters		
Relative biting rate (*rbr*)	Varied between simulations to modify the annual biting rate.	
Overall exposure rate of human hosts to central reservoir of infection (ζ)	Not applicable to onchocerciasis transmission ($\zeta = 1$)	
Seasonal variation in contribution to reservoir (*mbr*)	104%, 91%, 58%, 75%, 75%, 66%, 102%, 133%, 117%, 128%, 146%, and 105% times the average monthly biting rate (January–December)	[16]
Transmission probability (v), i.e. the probability that an infective particle in the reservoir successfully develops into a parasite life stage that is capable of infecting a human host	$v = 0.07345$; see reference for the derivation of this value, given parameters for fly biology and development of infective L3 larvae within the fly.	[11]
Success ratio (*sr*)	$sr = 0.31\%$	[17, 18]

Parameter	Value	Source
Zoophily (*z*)	$z = 0.04$.	[6], expert opinion (OCP entomologists)
Individual relative exposure to flies		
Variation in by age and sex (*Exa*)	Zero at birth, linearly increasing between ages 0–20 from 0 to 1.0 for men and from 0 to 0.7 for women, and then constant from the age of 20 years onwards	[17]
Variation due to personal factors (fixed through life) given age and sex (α_{Exi})	Gamma distribution with mean 1.0 and shape and rate equal to 3.5	[17], unpublished data from OCP
Individual relative contribution to infection in the fly population		
Variation by age and sex (*Coa*)	$Coa = Exa$; individual contribution and exposure to the cloud are perfectly correlated, given they are governed by the same fly bites.	Assumption
Variation due to personal factors (fixed through life) given age and sex (α_{Coi})	$Coi = Exi$; individual contribution and exposure to the cloud are perfectly correlated, given they are governed by the same fly bites.	Assumption
Host immunity to incoming infections		
Average impact of host immunity (α_{Imm})	Assumed irrelevant for onchocerciasis, hence $\alpha_{Imm} = 0$; i.e. no effect of immunity on incoming infections.	Assumption
Immunological memory (β_{Imm})	Irrelevant given that $\alpha_{Imm} = 0$.	Assumption
**Life history and productivity of the parasite in the human host**		
Average worm lifespan (*Tl*)	10 years	[2]
Variation in worm lifespan	Weibull distribution with shape 3.8.	Assumption [2]
Prepatent period (*pp*)	1 year	[2], which refers to [19, 20]
Age-dependent microfilaria production capacity (*R(a)*)	*R(a)* = 1 for 0 ≤ *a* < 5<br>*R(a)* = 1-((a-5)/15) for 5 ≤ *a* < 20<br>*R(a)* = 0 for *a* > 20	[2], which refers to [21, 22]
Longevity of microfilariae within host (*Tm*)	9 months	[17]
Mating cycle (*rc*)	3 months	[17], which refers to [23, 24]
Male potential (*pot*)	100 female worms.	[17]
Density-dependent female worm reproductive capacity		
Worm contribution to host load of infective material ($O(.)$)	7.6 mf/worm	[17]

Parameter	Value	Source
Exponential saturation of individual female worm productivity per worm present in host (λ_z)	$\lambda_z = 0$, i.e. no exponential saturation.	Assumption
**Morbidity**		
Disease threshold (*Elc*) for blindness	Weibull distribution with mean 10.000 and shape 2.0	[9]
Reduction in remaining life expectancy due to blindness (*rl*)	50%	[9], which refers to partly published data from OCP [25]; and [1], which refers to [26, 27]
**Infection dynamics in the cloud**		
Cloud uptake of infectious material ($U(.)$)	Exponential saturating function with parameters *a* = 1.2, *b* = 0.0213, and *c* = 0.0861 (see appendix II for the definition of an exponential saturating function).	[3], which refers to [28, 29]
Monthly cumulative survival of infective material in the central reservoir (ψ)	0%; i.e. the cloud represents a cloud of vectors that transmit infection within the same month.	Assumption
**Mass treatment coverage**		
Coverage (C_w)	User-defined.	

Relative compliance ($c_r(k,s)$) by age and sex (descriptive label used in graphs) — Source: Based on unpublished OCP data

age-group	cr(k,males)	cr(k,females)
0-4	0	0
5-9	0.75	0.5
10-14	0.8	0.7
15-19	0.8	0.74
20-29	0.7	0.65
30-49	0.75	0.7
50+	0.8	0.75

Parameter	Value	Source
**Drug treatment**		
Proportion of microfilariae cleared from host	100%	[5]
Duration of temporary reduction in female reproductive capacity (Tr_0), average	11 months	[5]
Permanent reduction in female worm reproductive capacity (d_0), average	34.9%	[5]
Proportion of adult worms killed (m_0)	0%	[5]
Relative effectiveness (v)	Weibull distribution with mean 1 and shape 2	[5]

Parameter	Value	Source
**Vector control**		
Timing	Not used.	
Coverage	Not used.	
**Surveys**		
Dispersal factor for worm contribution to measured density of infective material (d)	Exponential distribution with mean 1	[2]
Variability in measured host load of infective material (eggs per gram faeces)	Poisson distribution with mean $ss(t)$	[2]

5 Instructions for installing and running WORMSIM

5.1 Installing *WORMSIM*

Download and install the Java SE Runtime Environment 8 from
http://www.oracle.com/technetwork/java/javase/downloads/jre8-downloads-2133155.html

Download and unzip wormsim-2.58Ap9.zip to a location of your choice on your computer.

A folder named wormsim-2.58Ap9 will be created that contains:

- a number of example XML input files (all ending in .xml)
- *wormsim.xsd*, the XML Schema that is used to validate input files
- *wormsim.jar*, a Java archive with the .class and .java files of Wormsim
- *colt.jar*, the Colt library by Wolfgang Hoschek (CERN) that is used for statistical distributions
- *run.sh* and *run.bat*, a script / batch file to run Wormsim
- *avg.sh* and *avg.bat*, a script / batch file to aggregate the output of individual runs produced by running Wormsim
- *test.sh*, an example script / batch file that calls run.sh/run.bat and avg.sh/avg.bat
- *readme.txt*, a text file documenting the history of changes to Wormsim
- *license.txt*, a text file describing the license and conditions for using WORMSIM

5.2 Running *WORMSIM*

5.2.1 Microsoft Windows

Test the successful installation by:

- opening a DOS command line window by clicking on Start (Windows 7) and typing `cmd` and pressing enter. Navigate to the folder where WORMSIM has been installed, for instance (if you downloaded the zip file to your desktop and unzipped in that location):
  `cd .\Desktop\wormsim-2.58Ap9`
- if you do not have any experience with running batch files, you will find a tutorial at http://www.computerhope.com/issues/chusedos.htm
- running the test.bat batch file by typing:
  `.\test`
- after running *test.bat* you should find the following files in your WORMSIM folder:

  example_oncho.log

  example_oncho.txt

  example_oncho -19.zip, a zip file containing output of individual runs

- **see the supplement WORMSIM output documentation for details about the output files**

Copy the *example_oncho.xml* input file and edit this file for your specific scenario. To run WORMSIM with the new input file, copy and edit the *test.bat* batch file. Assuming you

copied *example_oncho.xml* to *my_oncho.xml* and *test.bat* to *my_test.bat*, you would edit the contents of the new *my_test.bat* as follows:

```
.\run.bat my_oncho.xml 0 99
.\avg.bat my_oncho.xml 0 99
```

and run your shell script with:

```
.\my_test
```

to do 100 runs and aggregate the output of these runs.

5.2.2 Mac OS X or Linux

Test the successful installation by:

- opening a Terminal window by running the Terminal program (to be found in Utilities) and navigating to the folder where Wormsim has been installed, for instance (if you downloaded the zip file to your desktop and unzipped in that location):
  `cd ~/Desktop/wormsim-2.58Ap9`
- if you do not have any experience with running shell scripts, you will find an excellent Unix/Linux tutorial at http://www.ee.surrey.ac.uk/Teaching/Unix/
- running the test.sh shell script by typing:
  `./test.sh`
- after running test.sh you should find the following files in your Wormsim folder:

  example_oncho.log

  example_oncho.txt

  example_oncho0-19.zip, a zip file containing output of individual runs
- see the supplement Wormsim output documentation for details about the output files

Copy the *example_oncho.xml* input file and edit this file for your specific scenario. To run WORMSIM with the new input file, copy and edit the *test.sh* shell script. Assuming you copied *example_STH.xml* to *my_STH.xml* and *test.sh* to *my_test.sh*, you would edit the contents of the new *my_test.sh* as follows:

```
./run.sh my_oncho.xml 0 99
./avg.sh my_oncho.xml 0 99
```

and run your shell script with:

```
./my_test.sh
```

to do 100 runs and aggregate the output of these runs

5.2.3 Output options

The -d output option will make WORMSIM produce additional detailed output. This output is found in **X.txt* and **Y.txt* (for instance *example_onchoX.txt* and *example_onchoY.txt*).

The –`n` output option suppresses all output except the **.log* output (e.g. *example_oncho.log*).
Either output option can be added to the *run* command as follows:

```
./run.sh my_oncho.xml 0 99 -d
```

or

```
./run.sh my_oncho.xml 0 99 -n
```

6 Annotated input file

The WORMSIM inputfile is an XML file that can be edited with any text editor or alternatively, with an XML editor (such as Oxygen XML Editor).
The advantage of using the XML format is that any input file can be validated against an XML Schema (a formal specification of the grammar used in the specific XML dialect used for the WORMSIM input file).

The Wormsim input file is documented with an annotated example (annotated-example.xml). The XML Schema (wormsim.xsd) is documented in great detail in schema-documentation-wormsim.pdf (provided with the software).

A copy of an annotated input file for ONCHOSIM is included below, split into fragments that cover the different elements of the input file (gray-shaded boxes). Together, these fragments constitute a complete input file.

- `Inputfile header`
- `Simulation`
- `Demography`
- `Blindness`
- `Exposure.and.contribution`
- `Immunity`
- `Worm`
- `Fly`
- `Mass.treatment`
- `Vector.control`

Meaning of the formatting of the input files:

- Text formatted in green as **`<!–- this is a comment -->`**) denotes a comment
- Grouping name tags for sets of input parameters are displayed in blue, while red indicates the specific parameters for which input is to be given.
- The actual inputs are found in the quotation marks, formatted in purple

6.1 *Inputfile header*

```
<?xml version="1.0" encoding="UTF-8"?>
<wormsim.inputfile xmlns:xsi="http://www.w3.org/2001/XMLSchema-instance"
                   xsi:noNamespaceSchemaLocation="wormsim.xsd"
                   model="onchosim">

    <!-- Input file for WORMSIM: -->
    <!-- Wormsim v2.58Ap9 -->
    <!-- Author: Wilma Stolk -->
    <!-- Date: 2015-07-30  -->
```

6.2 *<Simulation>*

The `<simulation>` element specifies the start year of the simulation, the timing of surveys (i.e. output moments), the number of skin snips taken at each survey and the age classes for output.

```
    <!-- general settings for simulation and simulation output       -->
    <simulation start.year="1800">
        <!-- number of skin snip taken per person                  -->
```

```
        <surveillance nr.skin-snips="2">
            <!-- timing of surveys                             -->
            <!-- month 0 represents January 1st                -->
            <!-- see note regarding "delay" below              -->
            <periodic.surveys>
                <start year="2000" month="6" delay="-2"/>
                <stop year="2000" month="7"/>
                <interval years="0" months="6"/>
            </periodic.surveys>
            <extra.surveys>
            <survey year="2002" month="6" delay="-2"/>
            </extra.surveys>
            <!-- upper bounds of age categories in output      -->
            <age.classes>
                <age.class age.limit="5"/>
                <age.class age.limit="90"/>
            </age.classes>
        </surveillance>
        <!-- upper bounds and weights of reference population for -->
        <!-- age and sex-standardized output (OCP standard pop)   -->
        <!-- in literature, the weight for age <5 is usually zero -->
        <standard.population>
            <age.group age.limit="5" n.males="0" n.females="0"/>
            <age.group age.limit="90" n.males="1000" n.females="1000"/>
        </standard.population>
    </simulation>
```

6.3 <Demography>

The <demography> element defines life tables for the male and female population, the maximum population size (above which random persons will be removed), a fertility table, and the initial population size and age distribution. See comments below.

```
    <!-- demographic parameters of simulated population              -->
    <demography>
        <!-- whenever the simulated population size exceeds the   -->
        <!-- the specified maximum, a random fraction is removed  -->
        <!-- see note regarding "delay" below                     -->
        <the.reaper max.population.size="440" reap="0.1" delay="-3"/>
        <!-- survival represents cumulative survival probability  -->
        <!-- and is determined by for unspecified ages by linear  -->
        <!-- interpolation of values for specified age limits     -->
        <life.table>
            <survival age.limit="5" male.survival="0.804" female.survival="0.804"/>
            <survival age.limit="10" male.survival="0.772" female.survival="0.772"/>
            <survival age.limit="15" male.survival="0.760" female.survival="0.760"/>
            <survival age.limit="20" male.survival="0.740" female.survival="0.740"/>
            <survival age.limit="30" male.survival="0.686" female.survival="0.686"/>
            <survival age.limit="50" male.survival="0.509" female.survival="0.509"/>
            <survival age.limit="90" male.survival="0.000" female.survival="0.000"/>
        </life.table>
        <!-- rates represent probabilies for women to give birth  -->
        <!-- to one child in some year, given a woman's age       -->
        <!-- rates are assumed constant within each age category  -->
        <!-- and ages limits represent upper bounds of categories -->
        <!-- see note regarding "delay" below                     -->
        <fertility.table delay="-4">
            <fertility age.limit="5" birth.rate="0"/>
            <fertility age.limit="10" birth.rate="0"/>
            <fertility age.limit="15" birth.rate="0"/>
            <fertility age.limit="20" birth.rate="0.109"/>
            <fertility age.limit="30" birth.rate="0.300"/>
            <fertility age.limit="50" birth.rate="0.119"/>
            <fertility age.limit="90" birth.rate="0.0"/>
        </fertility.table>
        <!-- population size to start simulation with            -->
        <initial.population>
            <age.group age.limit="5" n.males="4" n.females="4"/>
            <age.group age.limit="10" n.males="5" n.females="5"/>
            <age.group age.limit="15" n.males="3" n.females="3"/>
            <age.group age.limit="20" n.males="3" n.females="3"/>
            <age.group age.limit="30" n.males="4" n.females="4"/>
            <age.group age.limit="50" n.males="6" n.females="6"/>
```

```
        <age.group age.limit="90" n.males="4" n.females="4"/>
    </initial.population>
</demography>
```

6.4 <Blindness>

The `<blindness>` element defines the parameters for development of morbidity ("blindness" as originally developed for ONCHOSIM, where we specify a threshold of cumulative exposure to microfilaria) and the effect of morbidity on the remaining life expectancy.

```
<!-- parameters for development of blindness                    -->
<!-- when a person's cumulative exposure to mf exceeds a thres- -->
<!-- hold, a person is considered blind                         -->
<!-- individual variation in susceptibility is modeled by let-  -->
<!-- ting the threshold vary between individuals, assuming a    -->
<!-- Weibull distribution with some mean and shape "p1", trun-  -->
<!-- cated at the specified bounds "min" and "max"              -->
<blindness>
    <treshold dist.nr="3" min="0" max="100000" mean="10000" p1="2"/>
    <!-- upon turning blind, the life-expectancy of a person -->
    <!-- is reduced by a variable fraction: on average 50%,  -->
    <!-- and uniformly distributed between 0% and 100%       -->
    <pct-life-expectancy-reduction dist.nr="1" min="0" max="100" mean="50"/>
</blindness>
```

6.5 <Exposure.and.contribution>

The `<exposure.and.contribution>` element defines the parameters for the exposure of humans to a vector (or infectious reservoir) and the contribution of humans to the vector cloud (or infectious reservoir). See comments below.

```
<!-- parameters for exposure to fly bites                       -->
<!-- N.B. in WORMSIM we only describe fly bites on humans,      -->
<!-- whereas previously, ONCHOSIM also specified the fraction   -->
<!-- of fly bites on animals                                    -->
<exposure.and.contribution>
    <!-- initial force of infection to introduce infection   -->
    <!-- into the simulated population; duration in years    -->
    <initial.foi duration="7.5" foi="4.0"/>
    <!-- parameters for individual exposure to fly bites,    -->
    <!-- depending on gender, age, and personal factors      -->
    <male>
        <!-- age-dependent exposure, relative to mean exp -->
        <!-- of adult males, assuming a linear increase   -->
        <!-- between age 0 and 20, after which exposure   -->
        <!-- is 1.0                                       -->
        <exposure.function fun.nr="1" a="0.05" c="1"/>
        <!-- individual variation in exposure related to  -->
        <!-- e.g. occupation and attractiveness to flies, -->
        <!-- assuming a gamma distribution with mean one  -->
        <!-- and variation 1/p1 (shape and rate p1),      -->
        <!-- truncated by "min" and "max"                 -->
        <exposure.index dist.nr="4" min="0" max="20" p1="3.5"/>
    </male>
    <female>
        <!-- age-dependent exposure, relative to mean exp -->
        <!-- of adult males, assuming a linear increase   -->
        <!-- between age 0 and 20, after which exposure   -->
        <!-- is 1.0                                       -->
        <!-- exposure of females to fly bites is assumed  -->
        <!-- to be 70% of that of males                   -->
        <exposure.function fun.nr="1" a="0.035" c="0.7"/>
        <!-- individual variation in exposure related to  -->
        <!-- e.g. occupation and attractiveness to flies, -->
```

```
        <!-- assuming a gamma distribution with mean one  -->
        <!-- and variation 1/p1 (shape and rate p1),      -->
        <!-- truncated by "min" and "max"                 -->
        <exposure.index dist.nr="4" min="0" max="20" p1="3.5"/>
    </female>
</exposure.and.contribution>
```

6.6 <Immunity>

The <immunity> element defines the (optional) development of host immunity. Immunity is not considered in the current ONCHOSIM model.

```
<!-- parameters related to development of host immunity         -->
<!-- these are currently set such that no immunity develops     -->
<immunity>
    <male alpha="0" beta="1">
        <immunity.function fun.nr="0" a="1"/>
        <immunity.index dist.nr="0" min="0" max="20"/>
    </male>
    <female alpha="0" beta="1">
        <immunity.function fun.nr="0" a="1"/>
        <immunity.index dist.nr="0" min="0" max="20"/>
    </female>
</immunity>
```

6.7 <Worm>

The <worm> element defines parameters for worm lifespan, prepatent period, mating between M and F worms, age-dependent production of microfilaria, mf density per worm and skin dispersal.

```
<!-- parameters for worm survival and mf production             -->
<!-- mf lifespan in months, see note regarding "delay" below    -->
<worm mf-lifespan="9" monthly.event.delay="+1">
    <!-- worm lifespan in years, allowing for variation      -->
    <!-- between worms, assuming a Weibull distribution with -->
    <!-- mean 10 and shape 3.76, bounded by "min" and "max"  -->
    <lifespan dist.nr="3" min="0" max="50" mean="10" p1="3.76"/>
    <!-- pre-patent during which worms do not produce mf and -->
    <!-- are not affected by ivermectin                      -->
    <prepatent.period dist.nr="0" mean="1"/>
    <!-- number of months a female can produce mf with one   -->
    <!-- insemination, and number of females one male worm   -->
    <!-- can inseminate per month                            -->
    <!-- if there are more female worms than the total male  -->
    <!-- potential, every female has a probability of being  -->
    <!-- inseminated equal to N_mw/N_fm*male.potential       -->
    <mating cycle="3" male.potential="100"/>
    <!-- mf production by female worms as function of worm   -->
    <!-- age minus pre-patent period; mf production at un-   -->
    <!-- specified ages is determined by linear interpol.    -->
    <age.dependent.mf-production>
        <mf-production age.limit="0" production="1"/>
        <mf-production age.limit="5" production="1"/>
        <mf-production age.limit="20" production="0"/>
    </age.dependent.mf-production>
    <!-- expected N_mf per worm in skin snip as product of  -->
    <!-- number of mf contributed per fully fecund worm and -->
    <!-- random dispersal factor representing the distance  -->
    <!-- between a worm and site of skin snip, assuming an  -->
    <!-- exponential distribution, truncated by "min" and   -->
    <!-- "max"                                              -->
    <skin.mf-density.per.worm fun.nr="1" a="7.6" c="-1"/>
    <skin.dispersal dist.nr="2" min="0" max="5"/>
    <!-- poisson distribution for observed number of mf in  -->
    <!-- one skin snip                                      -->
    <skin-snip.variability dist.nr="5"/>
```

```
</worm>
```

6.8 <Fly>

The <fly> element defines parameters that determine the successful uptake and development of L1 larvae into infective L3 larvae and also determins the fly biting rate.

```
<!-- probability that an mf taken up by a fly bite develops    -->
<!-- into an L3 and is transmitted to another human (taking    -->
<!-- account of the fly's gonotropic cycle, survival, and      -->
<!-- duration and probability of an ingested mf developing     -->
<!-- into an infective L3 and surviving up to the point of     -->
<!-- transmission                                               -->
<fly transmission.probability="0.07345">
    <!-- functional relation between uptake of mf and mf    -->
    <!-- density in the skin, assuming exponential satu-    -->
    <!-- ration to maximum level a with initial slope 2b    -->
    <!-- nd shape c                                          -->
    <L1-uptake fun.nr="3" a="1.2" b="0.0213" c="0.0861"/>
    <!-- seasonal pattern in monthly biting rates (mbr),    -->
    <!-- as observed in Asubende, Ghana                      -->
    <!-- in the simulation, actual biting rates for an      -->
    <!-- individual are calculated as product of monthly    -->
    <!-- biting rate in Asubende, a factor representing     -->
    <!-- the mean exposure in adult males in the simulated  -->
    <!-- village relative to Asubende ("relative biting     -->
    <!-- rate"), and all other factors related to gender,   -->
    <!-- age, and individual variation in exposure          -->
    <!-- to produce some desired endemicity level in the    -->
    <!-- simulation, adjust the relative biting rate such   -->
    <!-- that mf prevalence or density (distribution) in    -->
    <!-- the population (output at the desired time point)  -->
    <!-- equals the desired value                           -->
    <!-- N.B. individual variation in exposure to fly bites -->
    <!-- also determined mean and distribution of simulated -->
    <!-- infection levels                                   -->
    <!-- rbr = 0.305 for CMFL 5-->
    <!-- rbr = 0.329 for CMFL 10-->
    <!-- rbr = 0.457 for CMFL 30-->
    <!-- rbr = 0.586 for CMFL 55-->
    <!-- rbr = 0.720 for CMFL 80-->
    <monthly.biting.rates relative.biting.rate="0.305">
        <mbr month="1"  rate="2670"/>
        <mbr month="2"  rate="2350"/>
        <mbr month="3"  rate="1500"/>
        <mbr month="4"  rate="1920"/>
        <mbr month="5"  rate="1940"/>
        <mbr month="6"  rate="1690"/>
        <mbr month="7"  rate="2630"/>
        <mbr month="8"  rate="3410"/>
        <mbr month="9"  rate="3010"/>
        <mbr month="10" rate="3290"/>
        <mbr month="11" rate="3750"/>
        <mbr month="12" rate="2690"/>
    </monthly.biting.rates>
</fly>
<!-- parameters for mass treatment                             -->
```

6.9 <Mass.treatment>

The <mass.treatment> element defines parameters for the timing of mass treatment rounds, individual compliance (permanent, temporary and age dependent), and effects of ivermectin on mature worms, mf production by F worms and on mf.

```
<mass.treatment>
    <!-- timing of individual mass treatment rounds (one   -->
    <!-- line per mass treatment round), specifying year,  -->
    <!-- month (0 represents January 1st), and population  -->
    <!-- coverage (fraction of total village population,   -->
```

```
        <!-- including those not eligible for treatment)       -->
        <!-- see note regarding "delay" below                  -->
        <treatment.rounds>
            <treatment.round year="2001" month="6" coverage="0.8" delay="-1"/>
        </treatment.rounds>
        <!-- random fraction of population permanently not      -->
        <!-- eligible for treatment due to chronic illness and  -->
        <!-- random fraction of population in which ivermectin  -->
        <!-- does not work due to diarrhoe (temporary effect)   -->
        <compliance fraction.excluded="0.05" fraction.malabsorption="0.05">
            <!-- weights for age and sex-specific compliance, given -->
            <!-- some expected overall coverage in the eligible     -->
            <!-- population                                         -->
            <!-- weights are constant within age categories         -->
            <age.and.sex.specific.compliance age.limit="5" male.compliance="0" female.compliance="0"/>
            <age.and.sex.specific.compliance age.limit="10" male.compliance="0.75"
female.compliance="0.75"/>
            <age.and.sex.specific.compliance age.limit="15" male.compliance="0.80"
female.compliance="0.70"/>
            <age.and.sex.specific.compliance age.limit="20" male.compliance="0.80"
female.compliance="0.74"/>
            <age.and.sex.specific.compliance age.limit="30" male.compliance="0.70"
female.compliance="0.65"/>
            <age.and.sex.specific.compliance age.limit="50" male.compliance="0.75"
female.compliance="0.70"/>
            <age.and.sex.specific.compliance age.limit="90" male.compliance="0.80"
female.compliance="0.75"/>
        </compliance>
        <!-- ivermectin efficacy, specified as permanent reduction in worm -->
        <!-- capacity to produce mf (cumulative effects allowed), pattern  -->
        <!-- of how mf production recovers over time (to a new, reduced    -->
        <!-- maximum level), and fraction of mf surviving each treatment   -->
        <treatment.effects permanent.reduction.mf-production="0.349" period.of.recovery="0.875"
shape.parameter.recovery.function="1.483" fraction.killed="0">
            <fraction.mf.surviving dist.nr="0" mean="0.0"/>
            <!-- variability in treatment effects (relative to mean,    -->
            <!-- expected effect), assuming a Weibull distribution with -->
            <!-- mean one and shape "p1"                                -->
            <treatment.effect.variability dist.nr="3" mean="1.0" p1="2"/>
        </treatment.effects>
    </mass.treatment>
```

6.10 <Vector.control>

The `<vector.control>` element defines parameters for setting the effectivity of vector control during periods of vector control.

```
    <!-- parameters for vector control                                    -->
    <!-- effectivity is specified as relative reduction in biting rates   -->
    <!-- if no vector control is desired in the simulation, set timing    -->
    <!-- outside the scope of the simulation (after the last survey)      -->
    <!-- vector control is usually assumed to be highly effective         -->
    <vector.control>
        <period start.year="2150" stop.year="2160" effectivity="0.95"/>
        <period start.year="2165" stop.year="2170" effectivity="0.95"/>
    </vector.control>
</wormsim.inputfile>
<!-- In ONCHOSIM, some events may be scheduled at the same time. The attribute     -->
<!-- "delay" specifies at what time an event takes place, relative to other events -->
<!-- planned at the same time. The order of events is currently: human births, the -->
<!-- reaper, survey, mass treatment, worm and mf generation and death.             -->
```

7 Annotated output file

The WORMSIM **standard output** (e.g ***example_oncho.txt***) is a tab delimited text file with the following columns:

year	: time (years)
N	: nr examined
N20	: nr examined > 20 yrs old
mf+	: percentage with positive skin snip
mf+20	: percentage with positive skin snip (> 20 yrs)
mfPr	: age/sex standardized mf prevalence
aNmf	: arithmetic mean nr mf per skin snip
aNmf20	: arithmetic mean nr mf per skin snip (> 20 yrs)
gNmf	: geometric meain nr mf per skin snip
CMFL	: geometric meain nr mf per skin snip (> 20 yrs)
bl	: percentage blind
bl20	: percentage blind (> 20 yrs)
blPr	: age/sex standardized prevalence of blindness
w+	: percentage with at least one adult female worm
w+20	: percentage with at least one adult female worm (> 20 yrs)
aNw	: arithmetic mean nr of adult female worms per person
aNw20	: arithmetic mean nr of adult female worms per person (>20 yrs)
mbr	: monthly fly biting rate in previous month
mtp	: monthly transmission potential in previous month
L1	: mean nr of L1 larvae per 1000 biting flies in previous month
L3	: mean nr of L3 larvae per 1000 biting flies in previous month
foi	: mean force of infection (nr of new adult worms per person) in prev. month

The WORMSIM **log output** (e.g ***example_oncho.log***) is a tab delimited text file with output for each simulation run in the following columns:

seed	: the seed of the random number generator (i.e. run nr) of that specific run
year	: time (years)
mf+	: fraction with positive skin snip
mf5+	: fraction with positive skin snip > 5 yrs
w+	: fraction with at least one adult female worm
N	: nr examined
aNmf	: arithmetic mean nr mf per skin snip
aNmf20	: arithmetic mean nr mf per skin snip (> 20 yrs)
N20	: nr examined > 20 yrs old
CMFL	: geometric meain nr mf per skin snip (> 20 yrs)

The detailed *X.txt output (e.g ***example_onchoX.txt***) contains sex and age specific output with the following columns:

year : time (years)
age : upper limit of age group
M : nr M examined in that age group
F : nr F examined in that age group
Mbl : percentage blind M in that age group
Fbl : percentage blind F in that age group
Mmfpos : percentage M in that age group with positive skin snip
Fmfpos : percentage F in that age group with positive skin snip
MaNmf : arithmetic mean nr mf per skin snip in M of that age group
FaNmf : arithmetic mean nr mf per skin snip in F of that age group
MgNmf : geometric mean nr mf per skin snip in M of that age group
FgNmf : geometric mean nr mf per skin snip in F of that age group
Mwpos : percentage M in that age with at least one adult female worm
Fwpos : percentage F in that age with at least one adult female worm
Mnrw : arithmetic mean nr of adult female worms per M in that age group
Fnrw : arithmetic mean nr of adult female worms per F in that age group

The detailed *Y.txt output (e.g ***example_onchoY.txt***) contains sex and age specific output on skin snips with the first two columns indicating year and age group

year : time (years)
age : upper limit of age group

The remaining columns depend on the skin snip categories defined in the input file.

The default value of the `skin-snip.categories` attribute of the `<surveillance>` element is

`skin-snip.categories="0.5,1,2,4,8,16,32,64,128,256,512,1e6"`

which results in the following output columns:
-1 : percentage of M in that age group with average skin snip count < 0.5
-1 : percentage of M in that age group with 0.5 <= average skin snip count < 1
-2 : percentage of M in that age group with 1 <= average skin snip count < 2
-4 : percentage of M in that age group with 2 <= average skin snip count < 4
-8 : percentage of M in that age group with 4 <= average skin snip count < 8
-16 : percentage of M in that age group with 8 <= average skin snip count < 16
-32 : percentage of M in that age group with 16 <= average skin snip count < 32
-64 : percentage of M in that age group with 32 <= average skin snip count < 64
-128 : percentage of M in that age group with 64 <= average skin snip count < 128
-256 : percentage of M in that age group with 128 <= average skin snip count < 256
-512 : percentage of M in that age group with 256 <= average skin snip count < 512
-1e9 : percentage of M in that age group with 512 <= average skin snip count < 1e9
followed by the same categories for F.

8 References

1. Plaisier, A.P., et al., *ONCHOSIM: a model and computer simulation program for the transmission and control of onchocerciasis.* Comput Methods Programs Biomed, 1990. **31**(1): p. 43-56.
2. Plaisier, A.P., et al., *The reproductive lifespan of Onchocerca volvulus in West African savanna.* Acta Trop, 1991. **48**(4): p. 271-84.
3. Plaisier, A.P., et al., *The risk and dynamics of onchocerciasis recrudescence after cessation of vector control.* Bull World Health Organ, 1991. **69**(2): p. 169-78.
4. Alley, E.S., et al., *Macrofilaricides and onchocerciasis control, mathematical modelling of the prospects for elimination.* BMC Public Health, 2001. **1**(12).
5. Plaisier, A.P., et al., *Irreversible effects of ivermectin on adult parasites in onchocerciasis patients in the Onchocerciasis Control Programme in West Africa.* J Infect Dis, 1995. **172**(1): p. 204-10.
6. Habbema, J.D.F., G.J. Oortmarssen, and A.P. Plaisier, *The ONCHOSIM model and its use in decision support for river blindness control*, in *Models for infectious human diseases - their stucture and relation to data*, V. Isham and G. Medley, Editors. 1996, Cambridge University Press: Cambridge. p. 360–380.
7. Plaisier, A.P., et al., *Required duration of combined annual ivermectin treatment and vector control in the Onchocerciasis Control Programme in west Africa.* Bull World Health Organ, 1997. **75**(3): p. 237-45.
8. Winnen, M., et al., *Can ivermectin mass treatments eliminate onchocerciasis in Africa?* Bull World Health Organ, 2002. **80**(5): p. 384-91.
9. Coffeng, L.E., et al., *African Programme For Onchocerciasis Control 1995-2015: model-estimated health impact and cost.* PLoS Negl Trop Dis, 2013. **7**(1): p. e2032.
10. Coffeng, L.E., et al., *African programme for onchocerciasis control 1995-2015: updated health impact estimates based on new disability weights.* PLoS Negl Trop Dis, 2014. **8**(6): p. e2759.
11. Coffeng, L.E., et al., *Elimination of African onchocerciasis: modeling the impact of increasing the frequency of ivermectin mass treatment.* PLoS One, 2014. **9**(12): p. e115886.
12. Plaisier, A.P., et al., *The LYMFASIM simulation program for modeling lymphatic filariasis and its control.* Methods of Information in Medicine, 1998. **37**: p. 97-108.
13. De Vlas, S.J., et al., *SCHISTOSIM: a microsimulation model for the epidemiology and control of schistosomiasis.* Am J Trop Med Hyg, 1996. **55**(5 Suppl): p. 170-5.
14. Coffeng, L.E., et al., *Feasibility of controlling hookworm infection through preventive chemotherapy: a simulation study using the individual-based WORMSIM modelling framework.* (submitted to Parasites and Vectors).
15. United Nations Department of Economic and Social Affairs Population Division, *World Population Prospects: the 2012 revision, Volume I: Comprehensive Tables.* 2013.
16. Alley, E.S., et al., *The impact of five years of annual ivermectin treatment on skin microfilarial loads in the onchocerciasis focus of Asubende, Ghana.* Trans R Soc Trop Med Hyg, 1994. **88**(5): p. 581-4.
17. Plaisier, A.P., *Modelling onchocerciasis transmission and control*, in *Department of Public Health.* 1996, Erasmus University Rotterdam: Rotterdam, the Netherlands. p. 181.
18. Duke, B.O., *The population dynamics of Onchocerca volvulus in the human host.* Trop Med Parasitol, 1993. **44**(2): p. 61-8.
19. Duke, B.O., *Observations on Onchocerca volvulus in experimentally infected chimpanzees.* Tropenmed Parasitol, 1980. **31**(1): p. 41-54.
20. Prost, A., *[Latency period in onchocerciasis].* Bull World Health Organ, 1980. **58**(6): p. 923-5.
21. Albiez, E.J., *Calcification in adult Onchocerca volvulus.* Trop Med Parasitol, 1985. **36**(3): p. 180-1.
22. Karam, M., H. Schulz-Key, and J. Remme, *Population dynamics of Onchocerca volvulus after 7 to 8 years of vector control in West Africa.* Acta Trop, 1987. **44**(4): p. 445-57.
23. Schulz-Key, H. and M. Karam, *Periodic reproduction of Onchocerca volvulus.* Parasitol Today, 1986. **2**(10): p. 284-6.
24. Schulz-Key, H., *Observations on the reproductive biology of Onchocerca volvulus.* Acta Leiden, 1990. **59**(1-2): p. 27-44.
25. Dadzie, K.Y., et al., *The effect of 7-8 years of vector control on the evolution of ocular onchocerciasis in West African savanna.* Trop Med Parasitol, 1986. **37**: p. 263-70.
26. Prost, A. and J. Vaugelade, *[Excess mortality among blind persons in the West African savannah zone].* Bull World Health Organ, 1981. **59**(5): p. 773-6.
27. Kirkwood, B., et al., *Relationships between mortality, visual acuity and microfilarial load in the area of the Onchocerciasis Control Programme.* Trans R Soc Trop Med Hyg, 1983. **77**(6): p. 862-8.

28. World Health Organization, *Onchocerciasis Control Programme in West Africa: report of the annual OCP research meeting, 20-24 March 1989*. (unpublished), 1989.
29. Phillipon, B., *Étude de la transmission d'Onchocerca volvulus (Leuckart, 1893) (Nematoda, Onchocercidae) par Simulium damnosum Theobald, 1903 (Diptera, Simuliidae) en Afrique tropicale.* . 1977, O.R.S.T.O.M.: Paris.

Appendix I Probability distributions for stochastic processes available in WORMSIM

Reference numbers (rightmost column) are used in the WORMSIM input file to define probability distributions for stochastic processes. Within WORMSIM, stochastic processes are pre-defined to follow either a continuous or discrete distribution, so each type has its own list of reference numbers.

Probability distribution	Parameters in input file	Domain	Probability density function	Reference number
Continuous distributions				
Constant (real)	μ	μ	$f(x) = 1$	0
Uniform	μ, p_1	$(p_1, 2\mu - p_1)$	$f(x) = (2\mu)^{-1}$	1
Exponential	μ	$\mathbb{R}^+$	$f(x) = \mu^{-1}e^{-x/\mu}$	2
Weibull	μ, p_1	$\mathbb{R}^+$	$f(x) = \alpha\beta^{-\alpha}x^{\alpha-1}e^{-(x/\beta)^{\alpha}}$, where $\alpha = p_1$ and $\beta = \mu/\Gamma(1 + 1/\alpha)$	3
Gamma	μ, p_1	$\mathbb{R}^+$	$f(x) = \frac{x^{k-1}e^{-x/\theta}}{\Gamma(k)\theta^k}$, where $k = p_1$ and $\theta = \mu/p_1$	4
Beta (optionally scaled)	μ, p_1, p_2	$(0, p_2)$	$f(x_1) = \frac{\Gamma(\alpha+\beta)}{\Gamma(\alpha)\Gamma(\beta)} x_{trans}^{\alpha-1}(1 - x_{trans})^{\beta-1}$, where $x_{trans} = x/p_2$, $\alpha = p_1$, and $\beta = p_1\left(\frac{p_2}{\mu} - 1\right)$	5
Normal	μ, p_1	$\mathbb{R}$	$f(x) = \frac{1}{\sqrt{2\pi\sigma^2}} e^{-(x-\mu)^2/2\sigma^2}$, where $\sigma = p_1$	6
Log-normal	μ, p_1	$\mathbb{R}^+$	$f(x) = \frac{1}{x\sqrt{2\pi\sigma_{ln}^2}} e^{-(\ln(x)-\mu_{ln})^2/2\sigma_{ln}^2}$, where $\mu_{ln} = \ln\left(\frac{\mu^2}{\sqrt{p_1^2+\mu^2}}\right)$, and $\sigma_{ln}^2 = \ln\left(\frac{p_1^2+\mu^2}{\mu^2}\right)$; i.e. μ and p_1 are defined on the positive real plane $\mathbb{R}^+$.	7
Discrete distributions				
Constant (integer)	μ	$\|\mu\|$	$f(x) = 1$	0
Bernoulli*	μ	$\mathbb{Z}_1$	$f(x) = \mu^x(1 - \mu)^{1-x}$	1
Uniform*	μ	$\mathbb{Z}_{j+1}$	$f(x) = \frac{1}{j+1}$, where $j = \lfloor(2m + 0.5)\rfloor$ and $\lfloor y \rfloor$ is the largest integer not larger than y	2
Binomial*	μ, p_1	$\mathbb{Z}_{p_1}$	$f(x) = \binom{n}{x}\mu^x(1 - \mu)^{n-x}$, where $n = p_1$	3
Geometric*	μ	$\mathbb{Z}^*$	$f(x) = (1 - p)^x p$, where $p = \frac{1}{\mu+1}$	4
Poisson	μ	$\mathbb{Z}^*$	$f(x) = \frac{e^{-\mu}\mu^x}{x!}$	5

**Probability distribution**	**Parameters in input file**	**Domain**	**Probability density function**	**Reference number**
Negative binomial	μ, p_1	$\mathbb{Z}^*$	$f(x) = \left(\frac{k}{k+\mu}\right)^k \frac{\Gamma(k+x)}{x!\Gamma(k)} \left(\frac{\mu}{k+\mu}\right)^x$, where $k = p_1$	6

* This distribution was available in the original ONCHOSIM model, but is still due to be implemented in WORMSIM. The Bernoulli distribution is used in parts of the model, but is hardcoded in these cases. The geometric distribution can be simulated by means of a negative binomial distribution with $p_1 = 1$. In a future update of WORMSIM, all listed distributions will be (re-)implemented using the Apache Commons Math library.

Appendix II Functional relationships available in WORMSIM

Reference numbers (rightmost column) are used in the WORMSIM input file to define functional relationships for deterministic processes.

**Functional relationship**	**Parameters**	**Formula**	**Parameter constraints and function characteristics**	**Reference number**
Constant	a	$f(x) = a$	$a \geq 0$	0
Linear	a, b, c	$f(x) = ax + b$ if $ax + b < c$,<br>$f(x) = c$ otherwise	$a \geq 0$; $b \geq 0$; if $c < 0$, no maximum is considered	1
Hyperbolic saturating	a, b, c	$f(x) = c + \frac{ax}{1 + ax/(b - c)}$	$b \geq 0$; $c \geq 0$; $(b - c)a > 0$;<br>$f(0) = 0$; $\lim_{x\to\infty} f(x) = b$;<br>$f'(0) = a$	2
Exponential saturating	a, b, c	$f(x) = a(1 - e^{-bx})(1 + e^{-cx})$	$a \geq 0$; $b > 0$; $c \geq 0$; $f(0) = 0$;<br>$\lim_{x\to\infty} f(x) = a$; $f'(0) = 2ab$	3
Sigmoidal saturating	a, b, c	$f(x) = a\left(1 - e^{-(bx)^c}\right)$	$a \geq 0$; $b > 0$; $0.1 < c < 10$;<br>$f(0) = 0$; $\lim_{x\to\infty} f(x) = a$;<br>$f'(0) = 0$ for $c > 1$;<br>$f'(0) = ab$ for $c = 1$;<br>$f'(0) = \infty$ for $c < 1$	4
Power function	a, b	$f(x) = ax^b$	$a \geq 0$; $b \geq 0$; $f(0) = 0$;<br>$\lim_{x\to\infty} f(x) = \infty$;<br>$f'(0) = 0$ for $b > 1$;<br>$f'(0) = a$ for $b = 1$;<br>$f'(0) = \infty$ for $b < 1$	5
