# Supplementary material for: Required duration of mass ivermectin treatment for onchocerciasis elimination in Africa: a comparative modelling analysis
Source: Parasit Vectors. 2015 Oct 22;8:552. doi: 10.1186/s13071-015-1159-9 (PMC4618738; doi:10.1186/s13071-015-1159-9)
Supplement: Additional file 2: — A zip-file, which includes the computer simulation program itself (with the JAVA program code embedded in it), batch files used to run the model, PDF documentation of the XML input, and example input and output files. Instructions on how to run the model are provided in Additional file 1 (ONCHOSIM simulation program.zip). (DOCX 15 kb) [file 13071_2015_1159_MOESM2_ESM.zip › wormsim-schema-documentation.pdf]

Schema documentation for wormsim.xsd

august 19, 2015

Table of Contents

Namespace: "" ... 4
Schema(s) ... 4
Main schema `wormsim.xsd` ... 4
Element(s) ... 4
Element `age.table / age.class` ... 4
Element `population.table / age.group` ... 4
Element `MM.kinetics / a` ... 4
Element `MM.kinetics / b` ... 5
Element `MM.kinetics / c` ... 6
Element `wormsim.inputfile` ... 7
Element `simulation` ... 8
Element `surveillance` ... 9
Element `periodic.surveys` ... 10
Element `periodic.surveys / start` ... 10
Element `periodic.surveys / stop` ... 11
Element `periodic.surveys / interval` ... 11
Element `extra.surveys` ... 11
Element `extra.surveys / survey` ... 12
Element `surveillance / age.classes` ... 12
Element `simulation / standard.population` ... 13
Element `demography` ... 13
Element `the.reaper` ... 13
Element `life.table` ... 14
Element `survival` ... 14
Element `fertility.table` ... 15
Element `fertility` ... 15
Element `demography / initial.population` ... 16
Element `blindness` ... 16
Element `treshold` ... 16
Element `pct-life-expectancy-reduction` ... 17
Element `exposure.and.contribution` ... 18
Element `exposure.and.contribution / environment` ... 19
Element `initial.foi` ... 19
Element `male` ... 19
Element `male / exposure.function` ... 20
Element `male / age.dependent.exposure` ... 21
Element `age.dependency.table / age.dependency` ... 21
Element `male / exposure.index` ... 21
Element `male / contribution.function` ... 22
Element `male / age.dependent.contribution` ... 23
Element `male / contribution.index` ... 23
Element `female` ... 24
Element `female / exposure.function` ... 25
Element `female / age.dependent.exposure` ... 25
Element `female / exposure.index` ... 25
Element `female / contribution.function` ... 26
Element `female / age.dependent.contribution` ... 27
Element `female / contribution.index` ... 27
Element `immunity` ... 28
Element `immunity / male` ... 28
Element `immunity.function` ... 29
Element `immunity.index` ... 30
Element `immunity / female` ... 30
Element `worm` ... 31
Element `lifespan` ... 32
Element `prepatent.period` ... 33
Element `mating` ... 33
Element `age.dependent.mf-production` ... 34
Element `mf-production` ... 34
Element `skin.mf-density.per.worm` ... 35
Element `alt.skin.mf-density.per.worm` ... 35
Element `skin.dispersal` ... 36
Element `skin-snip.variability` ... 37

Element fly 37
Element L1-uptake 38
Element monthly.biting.rates 38
Element mbr 39
Element snails 39
Element egg-uptake 40
Element monthly.birth.rates 40
Element snail-population 41
Element snail-population / Y0 41
Element snail-population / Y1 42
Element snail-population / Y2 42
Element mass.treatment 42
Element treatment.rounds 43
Element treatment.round 43
Element compliance 44
Element age.and.sex.specific.compliance 44
Element treatment.effects 45
Element treatment.effects / fraction.mf.surviving 46
Element treatment.effects / treatment.effect.variability 46
Element vector.control 47
Element period 48
Complex Type(s) 48
Complex Type age.table 48
Complex Type age.class 48
Complex Type population.table 49
Complex Type age.group 49
Complex Type functional.relationship 49
Complex Type MM.kinetics 50
Complex Type continuous.distribution 50
Complex Type discrete.distribution 51
Complex Type ym 51
Complex Type intervalym 52
Complex Type environment.definition 52
Complex Type age.dependency.table 53
Simple Type(s) 53
Simple Type nonnegint200 53
Simple Type nonnegativeinteger 53
Simple Type function.nrs 54
Simple Type continuous.distribution.nrs 54
Simple Type discrete.distribution.nrs 54
Simple Type deltahours 54
Simple Type monthnr 55
Simple Type yearnr 55
Simple Type modeltype 55
Simple Type nonnegdouble1 56
Simple Type nonnegdouble 56
Simple Type covmodel 56
Attribute(s) 57
Attribute age.class / @age.limit 57
Attribute age.group / @age.limit 57
Attribute age.group / @n.males 57
Attribute age.group / @n.females 57
Attribute functional.relationship / @fun.nr 58
Attribute functional.relationship / @a 58
Attribute functional.relationship / @b 58
Attribute functional.relationship / @c 58
Attribute continuous.distribution / @dist.nr 58
Attribute continuous.distribution / @offset 58
Attribute continuous.distribution / @min 59
Attribute continuous.distribution / @max 59
Attribute continuous.distribution / @mean 59
Attribute continuous.distribution / @p1 59
Attribute continuous.distribution / @p2 59
Attribute discrete.distribution / @dist.nr 60
Attribute discrete.distribution / @mean 60
Attribute discrete.distribution / @p1 60
Attribute discrete.distribution / @p2 60
Attribute ym / @year 60
Attribute ym / @month 60
Attribute ym / @delay 61
Attribute intervalym / @years 61
Attribute intervalym / @months 61

Attribute surveillance / @nr.skin-snips 61
Attribute surveillance / @skin-snip.categories 62
Attribute simulation / @start.year 62
Attribute the.reaper / @max.population.size 62
Attribute the.reaper / @reap 62
Attribute the.reaper / @delay 62
Attribute survival / @age.limit 63
Attribute survival / @male.survival 63
Attribute survival / @female.survival 63
Attribute fertility / @age.limit 63
Attribute fertility / @birth.rate 63
Attribute fertility.table / @fraction.male.newborns 64
Attribute fertility.table / @delay 64
Attribute environment.definition / @zeta 64
Attribute environment.definition / @psi 64
Attribute initial.foi / @duration 64
Attribute initial.foi / @foi 65
Attribute age.dependency.table / age.dependency / @age 65
Attribute age.dependency.table / age.dependency / @x 65
Attribute immunity / male / @alpha 65
Attribute immunity / male / @beta 65
Attribute immunity / female / @alpha 66
Attribute immunity / female / @beta 66
Attribute mating / @sex.ratio 66
Attribute mating / @cycle 66
Attribute mating / @male.potential 66
Attribute mf-production / @age.limit 67
Attribute mf-production / @production 67
Attribute age.dependent.mf-production / @labda 67
Attribute worm / @mf-lifespan 67
Attribute worm / @monthly.event.delay 67
Attribute mbr / @month 68
Attribute mbr / @rate 68
Attribute monthly.biting.rates / @relative.biting.rate 68
Attribute fly / @transmission.probability 68
Attribute monthly.birth.rates / @relative.birth.rate 68
Attribute snail-population / Y0 / @initial 69
Attribute snail-population / Y0 / @mu 69
Attribute snail-population / Y1 / @initial 69
Attribute snail-population / Y1 / @mu 69
Attribute snail-population / Y1 / @sigma 69
Attribute snail-population / Y2 / @initial 70
Attribute snail-population / Y2 / @mu 70
Attribute snails / @transmission.probability 70
Attribute treatment.round / @year 70
Attribute treatment.round / @month 70
Attribute treatment.round / @delay 71
Attribute treatment.round / @coverage 71
Attribute age.and.sex.specific.compliance / @age.limit 71
Attribute age.and.sex.specific.compliance / @male.compliance 71
Attribute age.and.sex.specific.compliance / @female.compliance 71
Attribute compliance / @fraction.excluded 72
Attribute compliance / @fraction.malabsorption 72
Attribute compliance / @compliance.model 72
Attribute compliance / @test.first 72
Attribute treatment.effects / @permanent.reduction.mf-production 72
Attribute treatment.effects / @period.of.recovery 73
Attribute treatment.effects / @shape.parameter.recovery.function 73
Attribute treatment.effects / @fraction.killed 73
Attribute period / @start.year 73
Attribute period / @start.month 73
Attribute period / @stop.year 74
Attribute period / @stop.month 74
Attribute period / @effectivity 74
Attribute wormsim.inputfile / @model 74

Namespace: ""

Schema(s)

Main schema wormsim.xsd

Namespace	No namespace
Properties	attribute form default: unqualified<br>element form default: qualified

Element(s)

Element age.table / age.class

Namespace	No namespace
Diagram	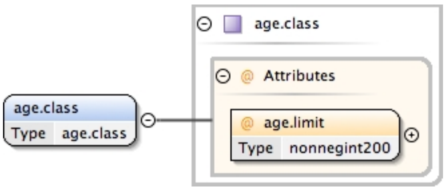
Type	age.class
Properties	content: complex<br>maxOccurs: unbounded
Attributes	**QName** / **Type** / **Use**<br>**age.limit** / nonnegint200 / required
Source	<xs:element maxOccurs="unbounded" name="age.class" type="age.class"/>

Element population.table / age.group

Namespace	No namespace
Diagram	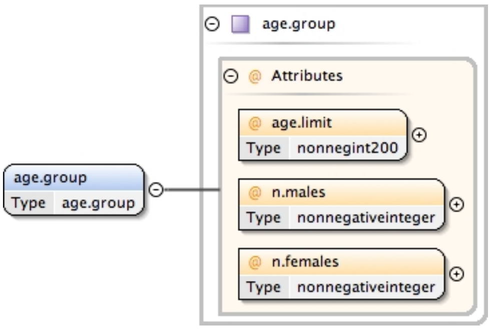
Type	age.group
Properties	content: complex<br>maxOccurs: unbounded
Attributes	**QName** / **Type** / **Use**<br>**age.limit** / nonnegint200 / required<br>**n.females** / nonnegativeinteger / required<br>**n.males** / nonnegativeinteger / required
Source	<xs:element maxOccurs="unbounded" name="age.group" type="age.group"/>

Element MM.kinetics / a

Namespace	No namespace

Diagram	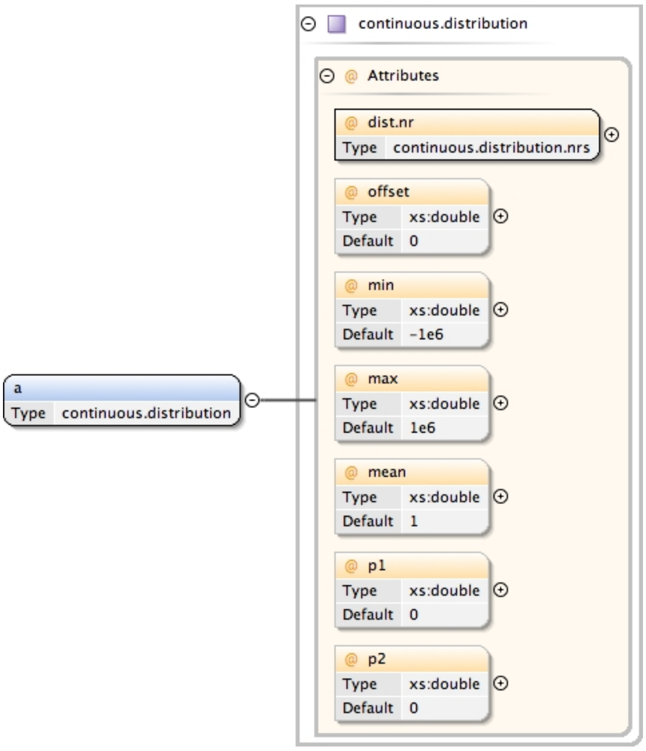
Type	continuous.distribution
Properties	content: `complex`
Attributes	**QName** / **Type** / **Default** / **Use** (see below)
Source	`<xs:element name="a" type="continuous.distribution"/>`

QName	Type	Default	Use	
**dist.nr**	continuous.distribution.nrs		required	
**max**	xs:double	1e6	optional	
**mean**	xs:double	1	optional	
**min**	xs:double	-1e6	optional	
**offset**	xs:double	0	optional	
**p1**	xs:double	0	optional	
**p2**	xs:double	0	optional	

Element MM.kinetics / b

Namespace	No namespace

Diagram

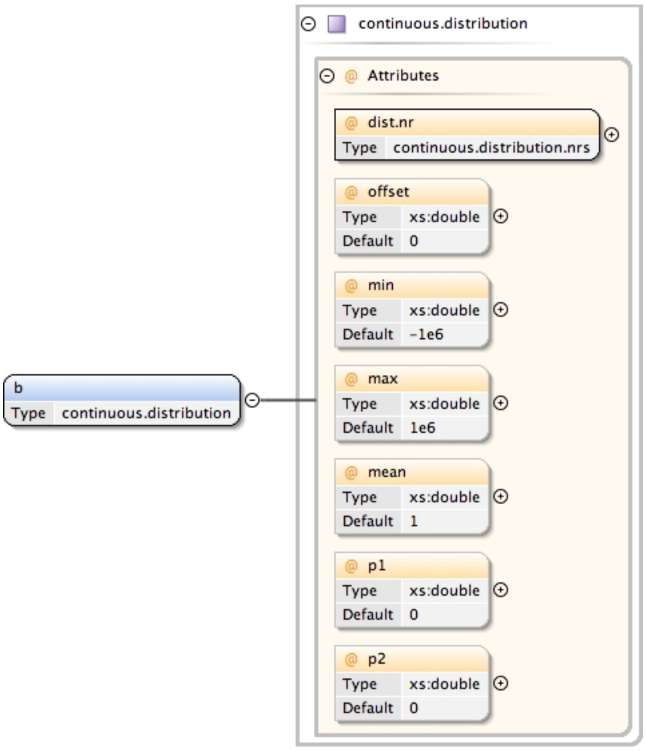

Type: continuous.distribution

Properties:

content:	`complex`

Attributes

QName	Type	Default	Use	
**dist.nr**	continuous.distribution.nrs		required	
**max**	xs:double	1e6	optional	
**mean**	xs:double	1	optional	
**min**	xs:double	-1e6	optional	
**offset**	xs:double	0	optional	
**p1**	xs:double	0	optional	
**p2**	xs:double	0	optional	

Source

```
<xs:element name="b" type="continuous.distribution"/>
```

Element `MM.kinetics / c`

Namespace	No namespace

<table>
<tr><td>Diagram</td><td>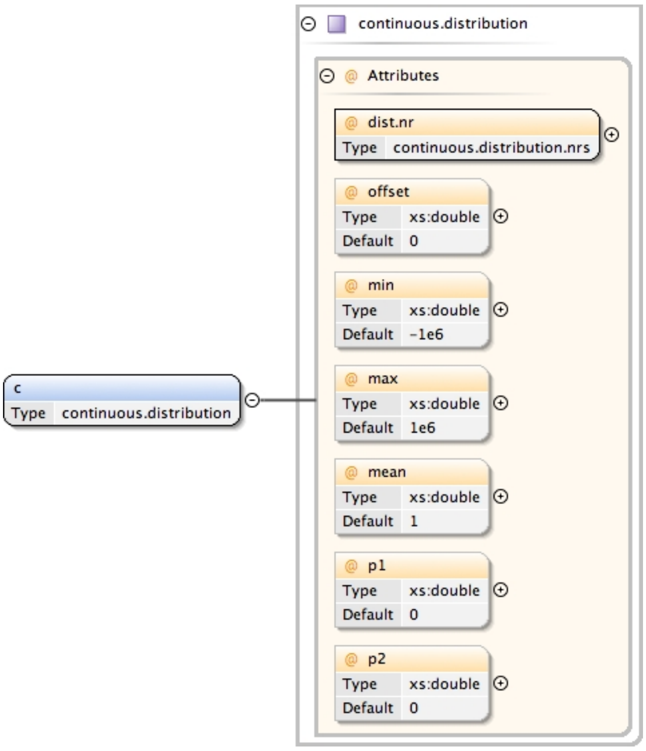
</td></tr>
<tr><td>Type</td><td>continuous.distribution</td></tr>
<tr><td>Properties</td><td>content: <code>complex</code></td></tr>
<tr><td>Attributes</td><td>
<table>
<tr><th>QName</th><th>Type</th><th>Default</th><th>Use</th><th></th></tr>
<tr><td>dist.nr</td><td>continuous.distribution.nrs</td><td></td><td>required</td><td></td></tr>
<tr><td>max</td><td>xs:double</td><td>1e6</td><td>optional</td><td></td></tr>
<tr><td>mean</td><td>xs:double</td><td>1</td><td>optional</td><td></td></tr>
<tr><td>min</td><td>xs:double</td><td>-1e6</td><td>optional</td><td></td></tr>
<tr><td>offset</td><td>xs:double</td><td>0</td><td>optional</td><td></td></tr>
<tr><td>p1</td><td>xs:double</td><td>0</td><td>optional</td><td></td></tr>
<tr><td>p2</td><td>xs:double</td><td>0</td><td>optional</td><td></td></tr>
</table>
</td></tr>
<tr><td>Source</td><td><code><xs:element name="c" type="continuous.distribution"/></code></td></tr>
</table>

Element `wormsim.inputfile`

Namespace	No namespace

<table>
<tr><td>Diagram</td><td>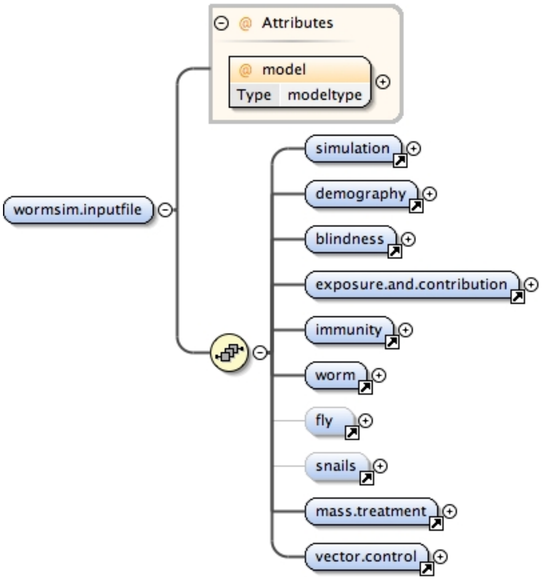
</td></tr>
<tr><td>Properties</td><td>content: complex</td></tr>
<tr><td>Model</td><td>simulation , demography , blindness , exposure.and.contribution , immunity , worm , fly{0,1} , snails{0,1} , mass.treatment , vector.control</td></tr>
<tr><td>Children</td><td>blindness, demography, exposure.and.contribution, fly, immunity, mass.treatment, simulation, snails, vector.control, worm</td></tr>
<tr><td>Instance</td><td><wormsim.inputfile model="">
  <simulation start.year="1875">{1,1}</simulation>
  <demography>{1,1}</demography>
  <blindness>{1,1}</blindness>
  <exposure.and.contribution>{1,1}</exposure.and.contribution>
  <immunity>{1,1}</immunity>
  <worm mf-lifespan="9" monthly.event.delay="-1">{1,1}</worm>
  <fly transmission.probability="">{0,1}</fly>
  <snails transmission.probability="">{0,1}</snails>
  <mass.treatment>{1,1}</mass.treatment>
  <vector.control>{1,1}</vector.control>
</wormsim.inputfile></td></tr>
<tr><td>Attributes</td><td><table><tr><th>QName</th><th>Type</th><th>Use</th><th></th></tr><tr><td>model</td><td>modeltype</td><td>required</td><td></td></tr></table></td></tr>
<tr><td>Source</td><td><xs:element name="wormsim.inputfile">
  <xs:complexType>
    <xs:sequence>
      <xs:element ref="simulation"/>
      <xs:element ref="demography"/>
      <xs:element ref="blindness"/>
      <xs:element ref="exposure.and.contribution"/>
      <xs:element ref="immunity"/>
      <xs:element ref="worm"/>
      <xs:element ref="fly" minOccurs="0" maxOccurs="1"/>
      <xs:element ref="snails" minOccurs="0" maxOccurs="1"/>
      <xs:element ref="mass.treatment"/>
      <xs:element ref="vector.control"/>
    </xs:sequence>
    <xs:attribute name="model" type="modeltype" use="required"/>
  </xs:complexType>
</xs:element></td></tr>
</table>

Element `simulation`

Namespace	No namespace

Diagram

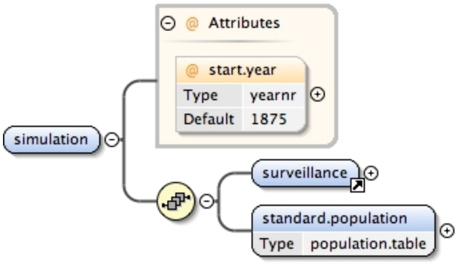

Properties	content: complex
Used by	Element wormsim.inputfile
Model	surveillance , standard.population
Children	standard.population, surveillance

Instance

```
<simulation start.year="1875">
  <surveillance nr.skin-snips="" skin-snip.categories="0.5,1,2,4,8,16,32,64,128,256,512,1e6">{1,1}</
surveillance>
  <standard.population>{1,1}</standard.population>
</simulation>
```

Attributes

QName	Type	Default	Use
**start.year**	yearnr	1875	optional

Source

```
<xs:element name="simulation">
  <xs:complexType>
    <xs:sequence>
      <xs:element ref="surveillance"/>
      <xs:element name="standard.population" type="population.table"/>
    </xs:sequence>
    <xs:attribute name="start.year" default="1875" type="yearnr"/>
  </xs:complexType>
</xs:element>
```

Element surveillance

Namespace	No namespace

Diagram

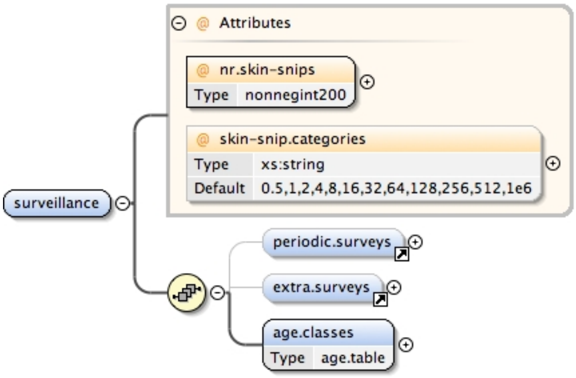

Properties	content: complex
Used by	Element simulation
Model	periodic.surveys{0,1} , extra.surveys{0,1} , age.classes
Children	age.classes, extra.surveys, periodic.surveys

Instance

```
<surveillance nr.skin-snips="" skin-snip.categories="0.5,1,2,4,8,16,32,64,128,256,512,1e6">
  <periodic.surveys>{0,1}</periodic.surveys>
  <extra.surveys>{0,1}</extra.surveys>
  <age.classes>{1,1}</age.classes>
</surveillance>
```

Attributes

QName	Type	Default	Use
**nr.skin-snips**	nonnegint200		required
**skin-snip.categories**	xs:string	0.5,1,2,4,8,16,32,64,128,256,512,1e6	optional

Source

```
<xs:element name="surveillance">
  <xs:complexType>
```

```
    <xs:sequence>
      <xs:element ref="periodic.surveys" minOccurs="0" maxOccurs="1"/>
      <xs:element ref="extra.surveys" minOccurs="0" maxOccurs="1"/>
      <xs:element name="age.classes" type="age.table"/>
    </xs:sequence>
    <xs:attribute name="nr.skin-snips" use="required" type="nonnegint200"/>
    <xs:attribute name="skin-snip.categories" default="0.5,1,2,4,8,16,32,64,128,256,512,1e6"
 type="xs:string"/>
  </xs:complexType>
</xs:element>
```

Element `periodic.surveys`

Namespace	No namespace
Diagram	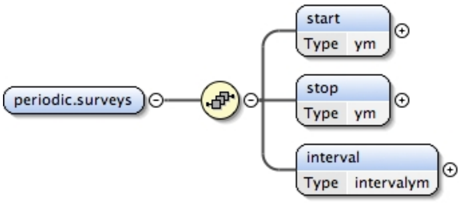
Properties	content: `complex`
Used by	Element surveillance
Model	start , stop , interval
Children	interval, start, stop

Instance

```
<periodic.surveys>
  <start delay="-5" month="0" year="">{1,1}</start>
  <stop delay="-5" month="0" year="">{1,1}</stop>
  <interval months="0" years="0">{1,1}</interval>
</periodic.surveys>
```

Source

```
<xs:element name="periodic.surveys">
  <xs:complexType>
    <xs:sequence>
      <xs:element name="start" minOccurs="1" maxOccurs="1" type="ym"/>
      <xs:element name="stop" minOccurs="1" maxOccurs="1" type="ym"/>
      <xs:element name="interval" minOccurs="1" maxOccurs="1" type="intervalym"/>
    </xs:sequence>
  </xs:complexType>
</xs:element>
```

Element `periodic.surveys / start`

Namespace	No namespace
Diagram	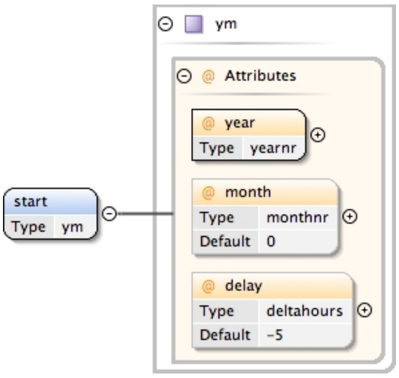
Type	ym
Properties	content: `complex`<br>minOccurs: `1`<br>maxOccurs: `1`

Attributes

QName	Type	Default	Use	
**delay**	deltahours	-5	optional	
**month**	monthnr	0	optional	

	QName	Type	Default	Use
	**year**	yearnr		required
Source	<xs:element name="start" minOccurs="1" maxOccurs="1" type="ym"/>			

Element **periodic.surveys / stop**

Namespace	No namespace
Diagram	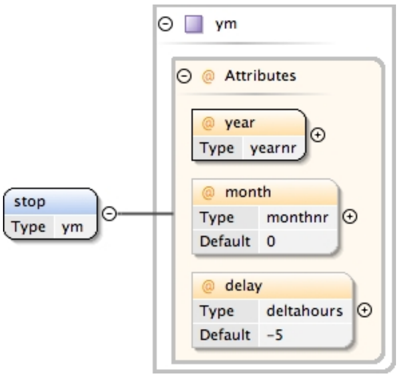
Type	ym
Properties	content: complex<br>minOccurs: 1<br>maxOccurs: 1

Attributes	QName	Type	Default	Use
	**delay**	deltahours	-5	optional
	**month**	monthnr	0	optional
	**year**	yearnr		required
Source	<xs:element name="stop" minOccurs="1" maxOccurs="1" type="ym"/>			

Element **periodic.surveys / interval**

Namespace	No namespace
Diagram	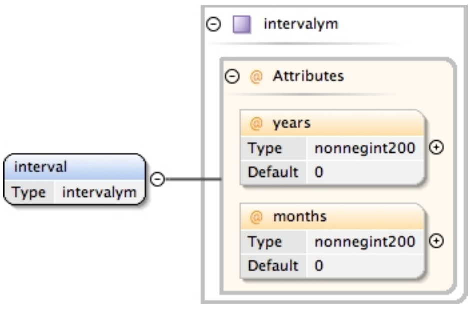
Type	intervalym
Properties	content: complex<br>minOccurs: 1<br>maxOccurs: 1

Attributes	QName	Type	Default	Use
	**months**	nonnegint200	0	optional
	**years**	nonnegint200	0	optional
Source	<xs:element name="interval" minOccurs="1" maxOccurs="1" type="intervalym"/>			

Element **extra.surveys**

Namespace	No namespace

Diagram	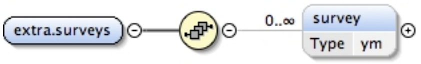
Properties	content: complex
Used by	Element surveillance
Model	survey*
Children	survey
Instance	<extra.surveys><br><survey delay="-5" month="0" year="">{0,unbounded}</survey><br></extra.surveys>
Source	<xs:element name="extra.surveys"><br><xs:complexType><br><xs:sequence><br><xs:element name="survey" minOccurs="0" maxOccurs="unbounded" type="ym"/><br></xs:sequence><br></xs:complexType><br></xs:element>

Element extra.surveys / survey

Namespace	No namespace
Diagram	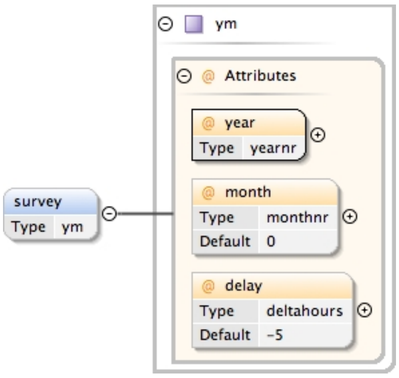
Type	ym
Properties	content: complex<br>minOccurs: 0<br>maxOccurs: unbounded
Attributes	see below
Source	<xs:element name="survey" minOccurs="0" maxOccurs="unbounded" type="ym"/>

QName	Type	Default	Use	
**delay**	deltahours	-5	optional	
**month**	monthnr	0	optional	
**year**	yearnr		required	

Element surveillance / age.classes

Namespace	No namespace
Diagram	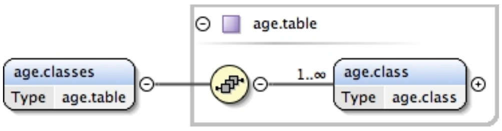
Type	age.table
Properties	content: complex
Model	age.class+
Children	age.class
Instance	<age.classes>

	<age.class age.limit="">{1,unbounded}</age.class><br></age.classes>
Source	<xs:element name="age.classes" type="age.table"/>

Element simulation / standard.population

Namespace	No namespace
Diagram	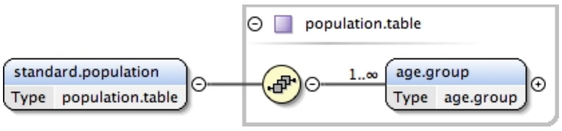
Type	population.table
Properties	content: complex
Model	age.group+
Children	age.group
Instance	<standard.population><br><age.group age.limit="" n.females="" n.males="">{1,unbounded}</age.group><br></standard.population>
Source	<xs:element name="standard.population" type="population.table"/>

Element demography

Namespace	No namespace
Diagram	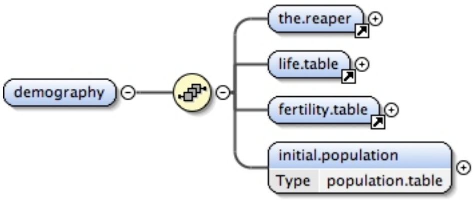
Properties	content: complex
Used by	Element wormsim.inputfile
Model	the.reaper , life.table , fertility.table , initial.population
Children	fertility.table, initial.population, life.table, the.reaper
Instance	<demography><br><the.reaper delay="-2" max.population.size="440" reap="0.1 ">{1,1}</the.reaper><br><life.table>{1,1}</life.table><br><fertility.table delay="-3" fraction.male.newborns="0.50">{1,1}</fertility.table><br><initial.population>{1,1}</initial.population><br></demography>
Source	<xs:element name="demography"><br><xs:complexType><br><xs:sequence><br><xs:element ref="the.reaper"/><br><xs:element ref="life.table"/><br><xs:element ref="fertility.table"/><br><xs:element name="initial.population" type="population.table"/><br></xs:sequence><br></xs:complexType><br></xs:element>

Element the.reaper

Namespace	No namespace

<table>
<tr><td>Diagram</td><td>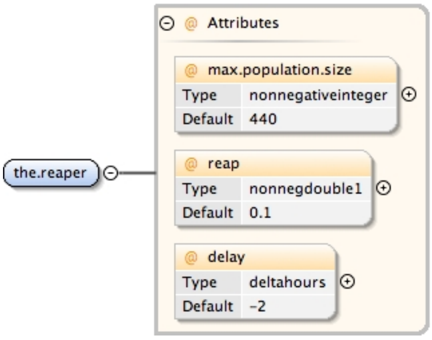
</td></tr>
<tr><td>Properties</td><td>content: complex</td></tr>
<tr><td>Used by</td><td>Element demography</td></tr>
<tr><td>Attributes</td><td><table>
<tr><th>QName</th><th>Type</th><th>Default</th><th>Use</th><th></th></tr>
<tr><td>delay</td><td>deltahours</td><td>-2</td><td>optional</td><td></td></tr>
<tr><td>max.population.size</td><td>nonnegativeinteger</td><td>440</td><td>optional</td><td></td></tr>
<tr><td>reap</td><td>nonnegdouble1</td><td>0.1</td><td>optional</td><td></td></tr>
</table></td></tr>
<tr><td>Source</td><td><pre><xs:element name="the.reaper">
  <xs:complexType>
    <xs:attribute name="max.population.size" use="optional" type="nonnegativeinteger" default="440"/
>
    <xs:attribute name="reap" use="optional" type="nonnegdouble1" default="0.1 "/>
    <xs:attribute name="delay" use="optional" type="deltahours" default="-2"/>
  </xs:complexType>
</xs:element></pre></td></tr>
</table>

Element `life.table`

<table>
<tr><td>Namespace</td><td>No namespace</td></tr>
<tr><td>Diagram</td><td>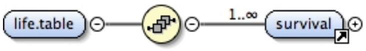
</td></tr>
<tr><td>Properties</td><td>content: complex</td></tr>
<tr><td>Used by</td><td>Element demography</td></tr>
<tr><td>Model</td><td>survival+</td></tr>
<tr><td>Children</td><td>survival</td></tr>
<tr><td>Instance</td><td><pre><life.table>
  <survival age.limit="" female.survival="" male.survival="">{1,unbounded}</survival>
</life.table></pre></td></tr>
<tr><td>Source</td><td><pre><xs:element name="life.table">
  <xs:complexType>
    <xs:sequence>
      <xs:element maxOccurs="unbounded" ref="survival"/>
    </xs:sequence>
  </xs:complexType>
</xs:element></pre></td></tr>
</table>

Element `survival`

<table>
<tr><td>Namespace</td><td>No namespace</td></tr>
<tr><td>Diagram</td><td>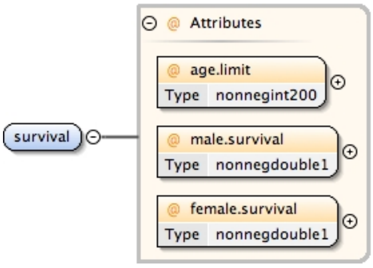
</td></tr>
<tr><td>Properties</td><td>content: complex</td></tr>
</table>

Used by	Element	life.table

Attributes

QName	Type	Use
**age.limit**	nonnegint200	required
**female.survival**	nonnegdouble1	required
**male.survival**	nonnegdouble1	required

Source

```
<xs:element name="survival">
  <xs:complexType>
    <xs:attribute name="age.limit" use="required" type="nonnegint200"/>
    <xs:attribute name="male.survival" use="required" type="nonnegdouble1"/>
    <xs:attribute name="female.survival" use="required" type="nonnegdouble1"/>
  </xs:complexType>
</xs:element>
```

Element `fertility.table`

Namespace	No namespace	
Diagram	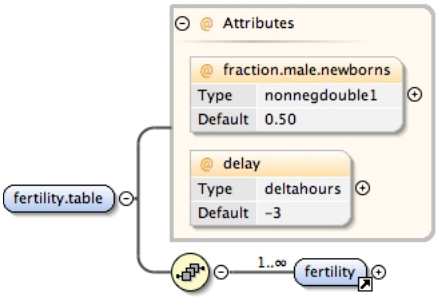	
Properties	content:	`complex`
Used by	Element	demography
Model	fertility+	
Children	fertility	

Instance

```
<fertility.table delay="-3" fraction.male.newborns="0.50">
  <fertility age.limit="" birth.rate="">{1,unbounded}</fertility>
</fertility.table>
```

Attributes

QName	Type	Default	Use
**delay**	deltahours	-3	optional
**fraction.male.newborns**	nonnegdouble1	0.50	optional

Source

```
<xs:element name="fertility.table">
  <xs:complexType>
    <xs:sequence>
      <xs:element maxOccurs="unbounded" ref="fertility"/>
    </xs:sequence>
    <xs:attribute name="fraction.male.newborns" type="nonnegdouble1" default="0.50"/>
    <xs:attribute name="delay" type="deltahours" default="-3"/>
  </xs:complexType>
</xs:element>
```

Element `fertility`

Namespace	No namespace	
Diagram	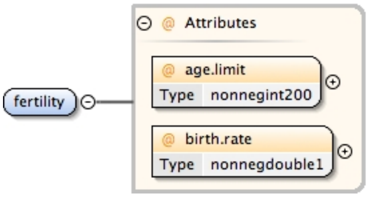	
Properties	content:	`complex`
Used by	Element	fertility.table

Attributes	**QName** / **Type** / **Use**<br>**age.limit** — nonnegint200 — required<br>**birth.rate** — nonnegdouble1 — required
Source	<xs:element name="fertility"><br><xs:complexType><br><xs:attribute name="age.limit" use="required" type="nonnegint200"/><br><xs:attribute name="birth.rate" use="required" type="nonnegdouble1"/><br></xs:complexType><br></xs:element>

Element demography / initial.population

Namespace	No namespace
Diagram	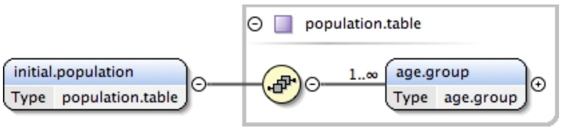
Type	population.table
Properties	content: complex
Model	age.group+
Children	age.group
Instance	<initial.population><br><age.group age.limit="" n.females="" n.males="">{1,unbounded}</age.group><br></initial.population>
Source	<xs:element name="initial.population" type="population.table"/>

Element blindness

Namespace	No namespace
Diagram	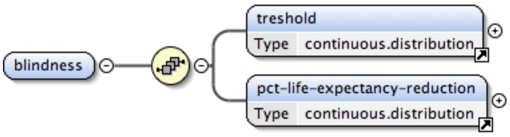
Properties	content: complex
Used by	Element wormsim.inputfile
Model	treshold , pct-life-expectancy-reduction
Children	pct-life-expectancy-reduction, treshold
Instance	<blindness><br><treshold dist.nr="" max="1e6" mean="1" min="-1e6" offset="0" p1="0" p2="0">{1,1}</treshold><br><pct-life-expectancy-<br>reduction dist.nr="" max="1e6" mean="1" min="-1e6" offset="0" p1="0" p2="0">{1,1}</pct-life-<br>expectancy-reduction><br></blindness>
Source	<xs:element name="blindness"><br><xs:complexType><br><xs:sequence><br><xs:element ref="treshold"/><br><xs:element ref="pct-life-expectancy-reduction"/><br></xs:sequence><br></xs:complexType><br></xs:element>

Element treshold

Namespace	No namespace

Diagram	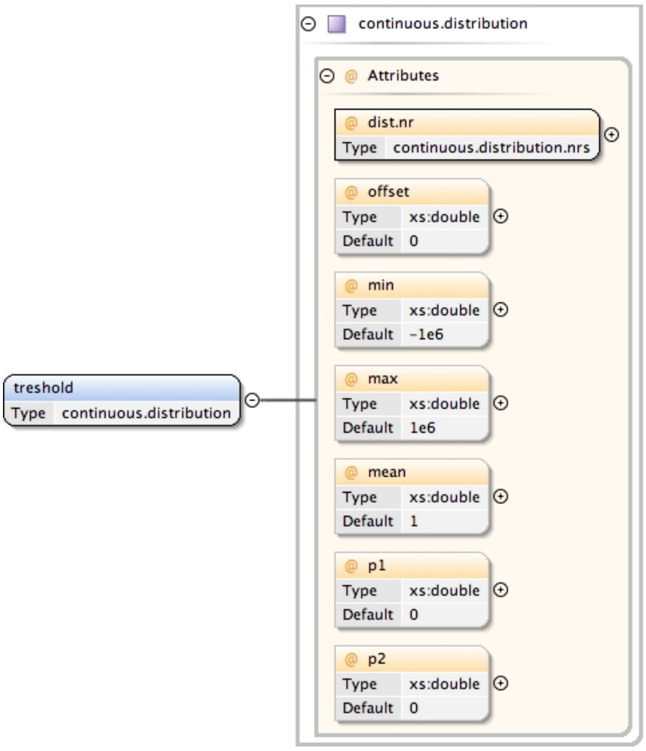
Type	continuous.distribution
Properties	content: complex
Used by	Element blindness
Attributes	

QName	Type	Default	Use	
**dist.nr**	continuous.distribution.nrs		required	
**max**	xs:double	1e6	optional	
**mean**	xs:double	1	optional	
**min**	xs:double	-1e6	optional	
**offset**	xs:double	0	optional	
**p1**	xs:double	0	optional	
**p2**	xs:double	0	optional	

Source:
```
<xs:element name="treshold" type="continuous.distribution"/>
```

Element `pct-life-expectancy-reduction`

Namespace	No namespace

Diagram

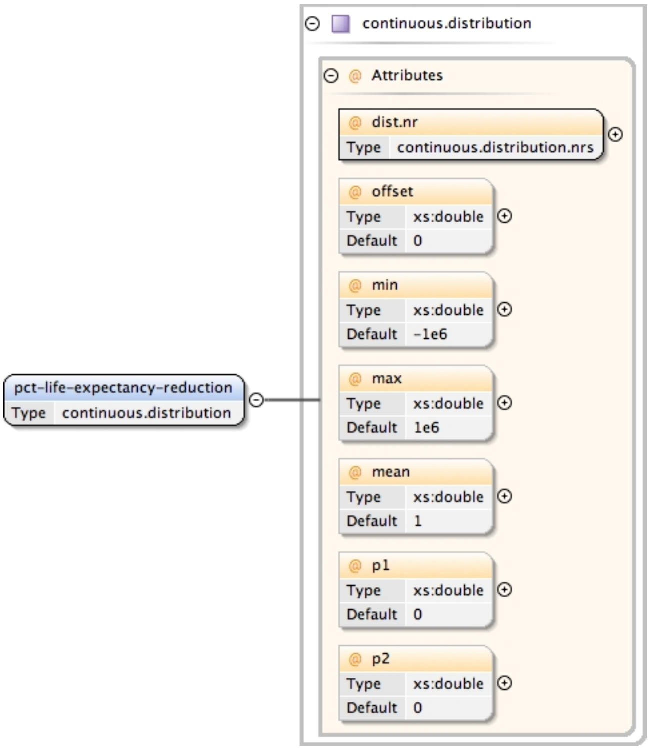

Type	continuous.distribution
Properties	content: `complex`
Used by	Element blindness

Attributes

QName	Type	Default	Use	
**dist.nr**	continuous.distribution.nrs		required	
**max**	xs:double	1e6	optional	
**mean**	xs:double	1	optional	
**min**	xs:double	-1e6	optional	
**offset**	xs:double	0	optional	
**p1**	xs:double	0	optional	
**p2**	xs:double	0	optional	

Source

```
<xs:element name="pct-life-expectancy-reduction" type="continuous.distribution"/>
```

Element `exposure.and.contribution`

Namespace	No namespace

Diagram

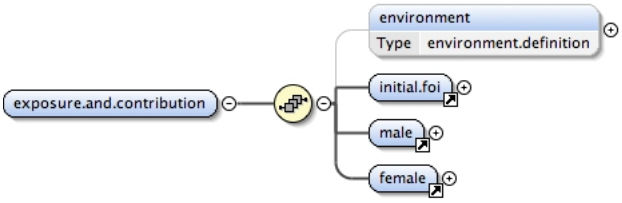

Properties	content: `complex`
Used by	Element wormsim.inputfile
Model	environment{0,1} , initial.foi , male , female
Children	environment, female, initial.foi, male

Instance

```
<exposure.and.contribution>
  <environment psi="" zeta="">{0,1}</environment>
  <initial.foi duration="" foi="">{1,1}</initial.foi>
  <male>{1,1}</male>
```

```
  <female>{1,1}</female>
</exposure.and.contribution>
```

Source

```
<xs:element name="exposure.and.contribution">
  <xs:complexType>
    <xs:sequence>
      <xs:element name="environment" type="environment.definition" minOccurs="0" maxOccurs="1"/>
      <xs:element ref="initial.foi"/>
      <xs:element ref="male"/>
      <xs:element ref="female"/>
    </xs:sequence>
  </xs:complexType>
</xs:element>
```

Element exposure.and.contribution / environment

Namespace	No namespace
Diagram	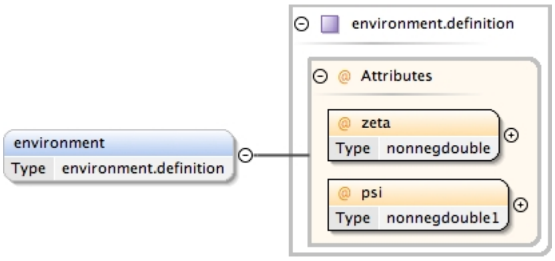
Type	environment.definition

Properties

content:	complex
minOccurs:	0
maxOccurs:	1

Attributes

QName	Type	Use	
**psi**	nonnegdouble1	required	
**zeta**	nonnegdouble	required	

Source

```
<xs:element name="environment" type="environment.definition" minOccurs="0" maxOccurs="1"/>
```

Element initial.foi

Namespace	No namespace
Diagram	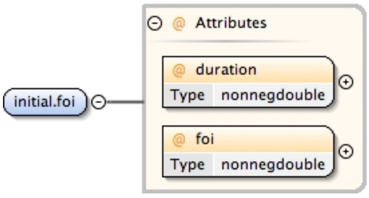

Properties

content:	complex

Used by

Element	exposure.and.contribution

Attributes

QName	Type	Use	
**duration**	nonnegdouble	required	
**foi**	nonnegdouble	required	

Source

```
<xs:element name="initial.foi">
  <xs:complexType>
    <xs:attribute name="duration" use="required" type="nonnegdouble"/>
    <xs:attribute name="foi" use="required" type="nonnegdouble"/>
  </xs:complexType>
</xs:element>
```

Element male

Namespace	No namespace

Diagram	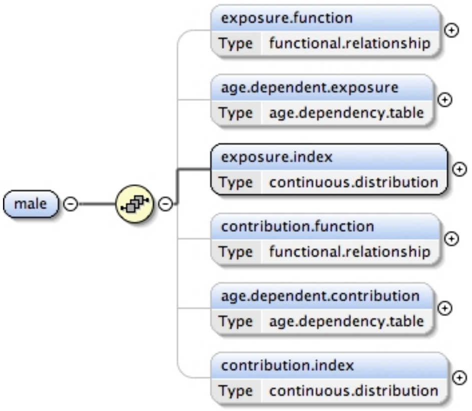
Properties	content: complex
Used by	Element exposure.and.contribution
Model	exposure.function{0,1} , age.dependent.exposure{0,1} , exposure.index , contribution.function{0,1} , age.dependent.contribution{0,1} , contribution.index{0,1}
Children	age.dependent.contribution, age.dependent.exposure, contribution.function, contribution.index, exposure.function, exposure.index

Instance

```xml
<male>
  <exposure.function a="" b="0" c="0" fun.nr="">{0,1}</exposure.function>
  <age.dependent.exposure>{0,1}</age.dependent.exposure>
  <exposure.index dist.nr="" max="1e6" mean="1" min="-1e6" offset="0" p1="0" p2="0">{1,1}</exposure.index>
  <contribution.function a="" b="0" c="0" fun.nr="">{0,1}</contribution.function>
  <age.dependent.contribution>{0,1}</age.dependent.contribution>
  <contribution.index dist.nr="" max="1e6" mean="1" min="-1e6" offset="0" p1="0" p2="0">{0,1}</contribution.index>
</male>
```

Source

```xml
<xs:element name="male">
  <xs:complexType>
    <xs:sequence>
      <xs:element name="exposure.function" type="functional.relationship" minOccurs="0" maxOccurs="1"/>
      <xs:element name="age.dependent.exposure" type="age.dependency.table" minOccurs="0" maxOccurs="1"/>
      <xs:element name="exposure.index" type="continuous.distribution"/>
      <xs:element name="contribution.function" type="functional.relationship" minOccurs="0" maxOccurs="1"/>
      <xs:element name="age.dependent.contribution" type="age.dependency.table" minOccurs="0" maxOccurs="1"/>
      <xs:element name="contribution.index" type="continuous.distribution" minOccurs="0" maxOccurs="1"/>
    </xs:sequence>
  </xs:complexType>
</xs:element>
```

Element male / exposure.function

Namespace	No namespace
Diagram	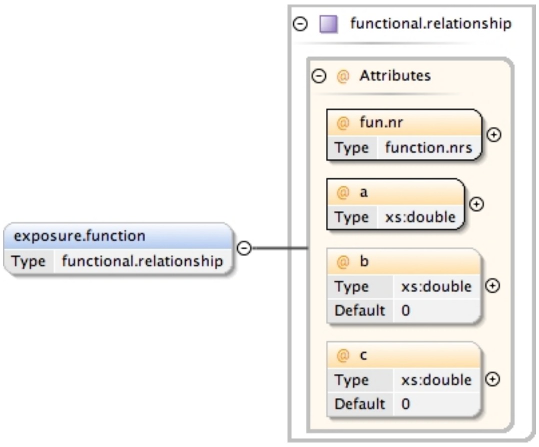
Type	functional.relationship

Properties	content: complex<br>minOccurs: 0<br>maxOccurs: 1
Attributes	**QName** / **Type** / **Default** / **Use**<br>**a** / xs:double / / required<br>**b** / xs:double / 0 / optional<br>**c** / xs:double / 0 / optional<br>**fun.nr** / function.nrs / / required
Source	`<xs:element name="exposure.function" type="functional.relationship" minOccurs="0" maxOccurs="1"/>`

Element male / age.dependent.exposure

Namespace	No namespace
Diagram	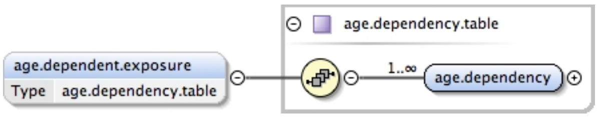
Type	age.dependency.table
Properties	content: complex<br>minOccurs: 0<br>maxOccurs: 1
Model	age.dependency+
Children	age.dependency
Instance	`<age.dependent.exposure>`<br>`<age.dependency age="" x="">{1,unbounded}</age.dependency>`<br>`</age.dependent.exposure>`
Source	`<xs:element name="age.dependent.exposure" type="age.dependency.table" minOccurs="0" maxOccurs="1"/>`

Element age.dependency.table / age.dependency

Namespace	No namespace
Diagram	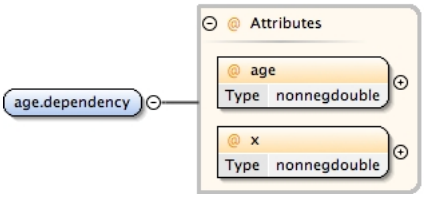
Properties	content: complex<br>maxOccurs: unbounded
Attributes	**QName** / **Type** / **Use**<br>**age** / nonnegdouble / required<br>**x** / nonnegdouble / required

Source:

```
<xs:element name="age.dependency" maxOccurs="unbounded">
  <xs:complexType>
    <xs:attribute name="age" use="required" type="nonnegdouble"/>
    <xs:attribute name="x" use="required" type="nonnegdouble"/>
  </xs:complexType>
</xs:element>
```

Element male / exposure.index

Namespace	No namespace

<table>
<tr><td>Diagram</td><td>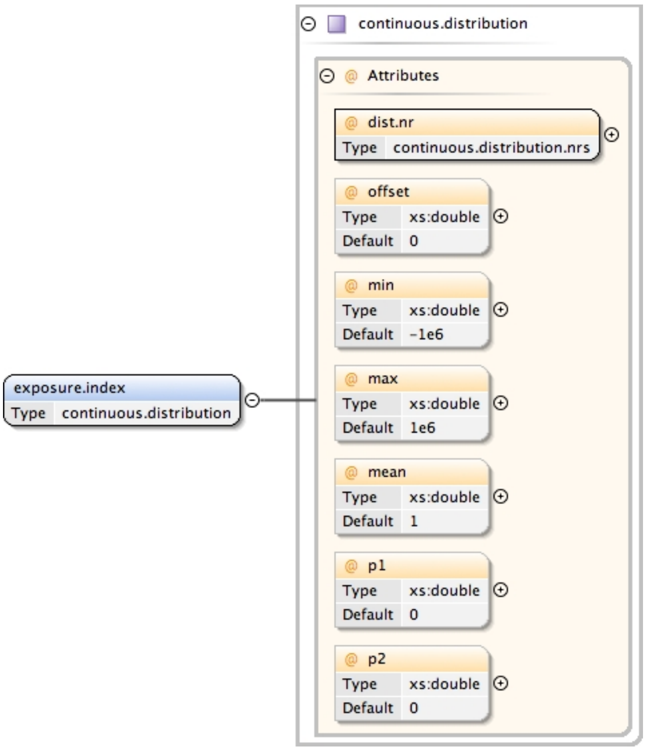
</td></tr>
<tr><td>Type</td><td>continuous.distribution</td></tr>
<tr><td>Properties</td><td>content: <code>complex</code></td></tr>
<tr><td>Attributes</td><td><table>
<tr><th>QName</th><th>Type</th><th>Default</th><th>Use</th><th></th></tr>
<tr><td>dist.nr</td><td>continuous.distribution.nrs</td><td></td><td>required</td><td></td></tr>
<tr><td>max</td><td>xs:double</td><td>1e6</td><td>optional</td><td></td></tr>
<tr><td>mean</td><td>xs:double</td><td>1</td><td>optional</td><td></td></tr>
<tr><td>min</td><td>xs:double</td><td>-1e6</td><td>optional</td><td></td></tr>
<tr><td>offset</td><td>xs:double</td><td>0</td><td>optional</td><td></td></tr>
<tr><td>p1</td><td>xs:double</td><td>0</td><td>optional</td><td></td></tr>
<tr><td>p2</td><td>xs:double</td><td>0</td><td>optional</td><td></td></tr>
</table></td></tr>
<tr><td>Source</td><td><code><xs:element name="exposure.index" type="continuous.distribution"/></code></td></tr>
</table>

Element `male / contribution.function`

<table>
<tr><td>Namespace</td><td>No namespace</td></tr>
<tr><td>Diagram</td><td>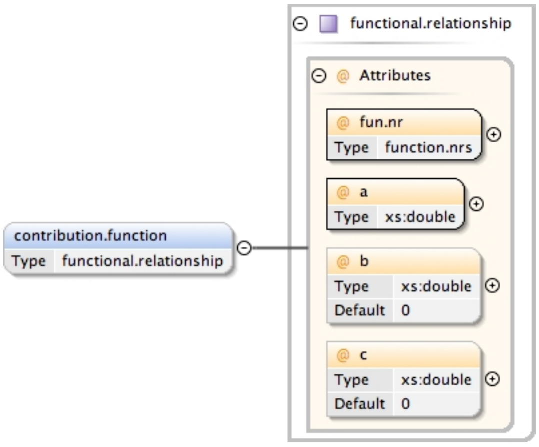
</td></tr>
<tr><td>Type</td><td>functional.relationship</td></tr>
<tr><td>Properties</td><td>content: <code>complex</code></td></tr>
</table>

	minOccurs:	0
	maxOccurs:	1
Attributes		

QName	Type	Default	Use	
**a**	xs:double		required	
**b**	xs:double	0	optional	
**c**	xs:double	0	optional	
**fun.nr**	function.nrs		required	

Source

```
<xs:element name="contribution.function" type="functional.relationship" minOccurs="0" maxOccurs="1"/
>
```

Element male / age.dependent.contribution

Namespace	No namespace
Diagram	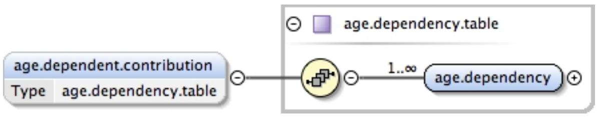
Type	age.dependency.table
Properties	content: complex<br>minOccurs: 0<br>maxOccurs: 1
Model	age.dependency+
Children	age.dependency
Instance	`<age.dependent.contribution>`<br>`<age.dependency age="" x="">{1,unbounded}</age.dependency>`<br>`</age.dependent.contribution>`
Source	`<xs:element name="age.dependent.contribution" type="age.dependency.table" minOccurs="0" maxOccurs="1"/>`

Element male / contribution.index

Namespace	No namespace
Diagram	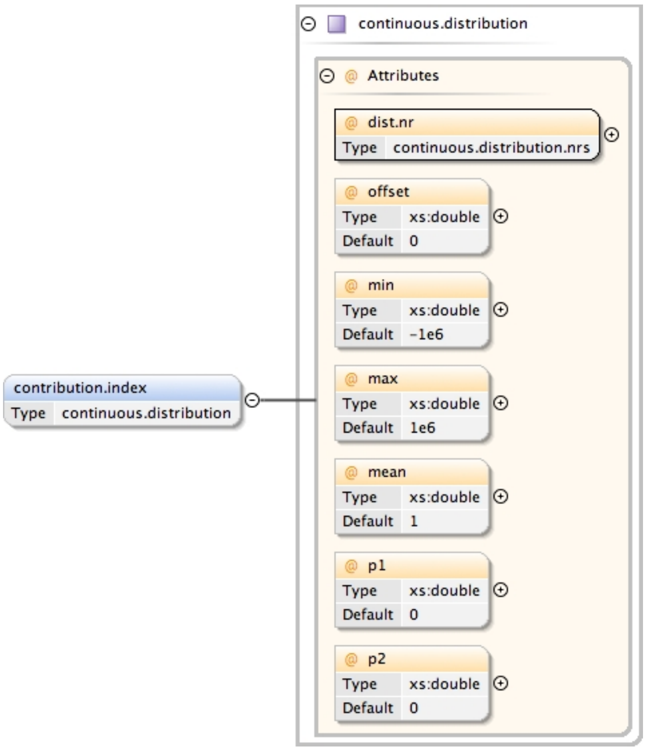
Type	continuous.distribution

Properties	content: complex<br>minOccurs: 0<br>maxOccurs: 1
Attributes	see below
Source	`<xs:element name="contribution.index" type="continuous.distribution" minOccurs="0" maxOccurs="1"/>`

QName	Type	Default	Use	
**dist.nr**	continuous.distribution.nrs		required	
**max**	xs:double	1e6	optional	
**mean**	xs:double	1	optional	
**min**	xs:double	-1e6	optional	
**offset**	xs:double	0	optional	
**p1**	xs:double	0	optional	
**p2**	xs:double	0	optional	

Element female

Namespace	No namespace
Diagram	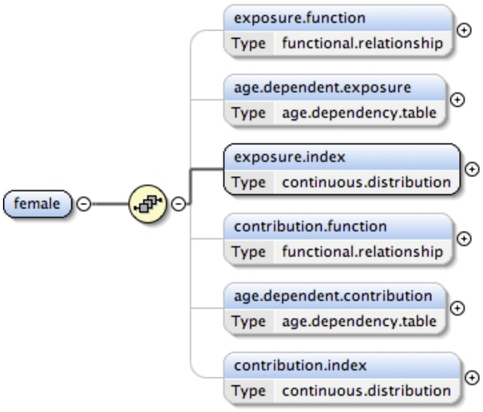
Properties	content: complex
Used by	Element exposure.and.contribution
Model	exposure.function{0,1} , age.dependent.exposure{0,1} , exposure.index , contribution.function{0,1} , age.dependent.contribution{0,1} , contribution.index{0,1}
Children	age.dependent.contribution, age.dependent.exposure, contribution.function, contribution.index, exposure.function, exposure.index

Instance

```
<female>
  <exposure.function a="" b="0" c="0" fun.nr="">{0,1}</exposure.function>
  <age.dependent.exposure>{0,1}</age.dependent.exposure>
  <exposure.index dist.nr="" max="1e6" mean="1" min="-1e6" offset="0" p1="0" p2="0">{1,1}</
exposure.index>
  <contribution.function a="" b="0" c="0" fun.nr="">{0,1}</contribution.function>
  <age.dependent.contribution>{0,1}</age.dependent.contribution>
  <contribution.index dist.nr="" max="1e6" mean="1" min="-1e6" offset="0" p1="0" p2="0">{0,1}</
contribution.index>
</female>
```

Source

```
<xs:element name="female">
  <xs:complexType>
    <xs:sequence>
      <xs:element name="exposure.function" type="functional.relationship" minOccurs="0"
 maxOccurs="1"/>
      <xs:element name="age.dependent.exposure" type="age.dependency.table" minOccurs="0"
 maxOccurs="1"/>
      <xs:element name="exposure.index" type="continuous.distribution"/>
      <xs:element name="contribution.function" type="functional.relationship" minOccurs="0"
 maxOccurs="1"/>
      <xs:element name="age.dependent.contribution" type="age.dependency.table" minOccurs="0"
 maxOccurs="1"/>
      <xs:element name="contribution.index" type="continuous.distribution" minOccurs="0"
 maxOccurs="1"/>
    </xs:sequence>
  </xs:complexType>
</xs:element>
```

Element female / exposure.function

<table>
<tr><td>Namespace</td><td>No namespace</td></tr>
<tr><td>Diagram</td><td>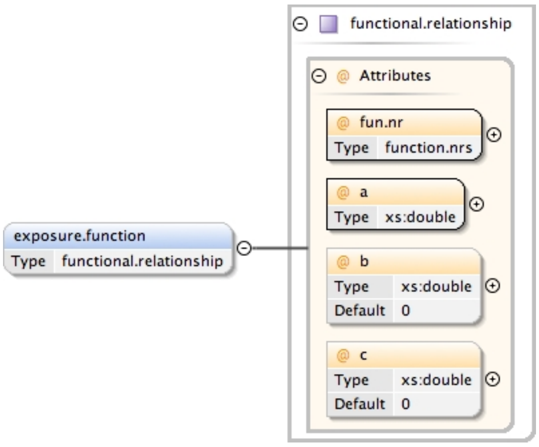
</td></tr>
<tr><td>Type</td><td>functional.relationship</td></tr>
<tr><td>Properties</td><td><table><tr><td>content:</td><td>complex</td></tr><tr><td>minOccurs:</td><td>0</td></tr><tr><td>maxOccurs:</td><td>1</td></tr></table></td></tr>
<tr><td>Attributes</td><td><table><tr><th>QName</th><th>Type</th><th>Default</th><th>Use</th><th></th></tr><tr><td>a</td><td>xs:double</td><td></td><td>required</td><td></td></tr><tr><td>b</td><td>xs:double</td><td>0</td><td>optional</td><td></td></tr><tr><td>c</td><td>xs:double</td><td>0</td><td>optional</td><td></td></tr><tr><td>fun.nr</td><td>function.nrs</td><td></td><td>required</td><td></td></tr></table></td></tr>
<tr><td>Source</td><td><xs:element name="exposure.function" type="functional.relationship" minOccurs="0" maxOccurs="1"/></td></tr>
</table>

Element female / age.dependent.exposure

<table>
<tr><td>Namespace</td><td>No namespace</td></tr>
<tr><td>Diagram</td><td>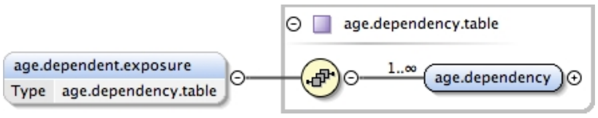
</td></tr>
<tr><td>Type</td><td>age.dependency.table</td></tr>
<tr><td>Properties</td><td><table><tr><td>content:</td><td>complex</td></tr><tr><td>minOccurs:</td><td>0</td></tr><tr><td>maxOccurs:</td><td>1</td></tr></table></td></tr>
<tr><td>Model</td><td>age.dependency+</td></tr>
<tr><td>Children</td><td>age.dependency</td></tr>
<tr><td>Instance</td><td><age.dependent.exposure>
  <age.dependency age="" x="">{1,unbounded}</age.dependency>
</age.dependent.exposure></td></tr>
<tr><td>Source</td><td><xs:element name="age.dependent.exposure" type="age.dependency.table" minOccurs="0" maxOccurs="1"/></td></tr>
</table>

Element female / exposure.index

Namespace	No namespace

Diagram	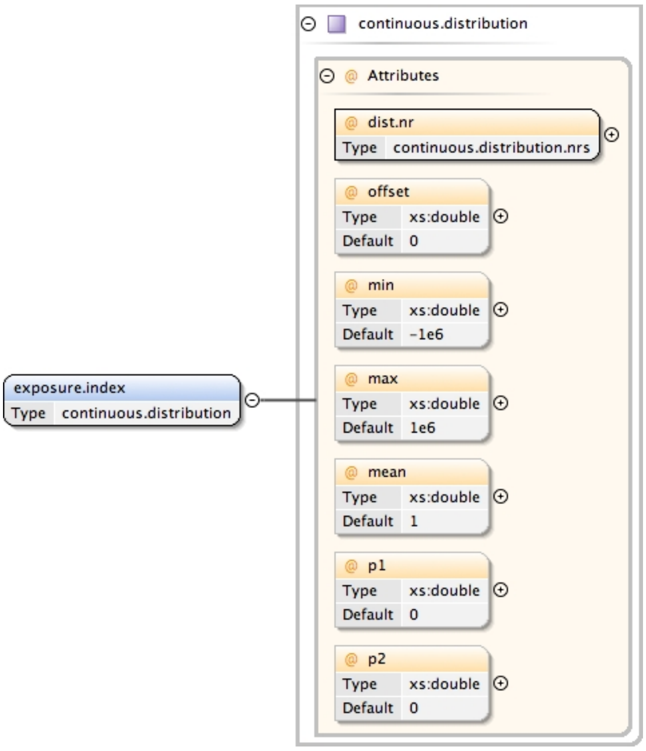
Type	continuous.distribution
Properties	content: complex
Attributes	see table below
Source	`<xs:element name="exposure.index" type="continuous.distribution"/>`

QName	Type	Default	Use
**dist.nr**	continuous.distribution.nrs		required
**max**	xs:double	1e6	optional
**mean**	xs:double	1	optional
**min**	xs:double	-1e6	optional
**offset**	xs:double	0	optional
**p1**	xs:double	0	optional
**p2**	xs:double	0	optional

Element female / contribution.function

Namespace	No namespace
Diagram	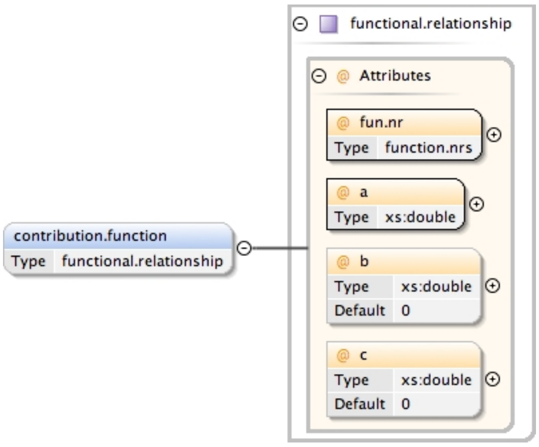
Type	functional.relationship
Properties	content: complex

	minOccurs:	0
	maxOccurs:	1

Attributes

QName	Type	Default	Use	
**a**	xs:double		required	
**b**	xs:double	0	optional	
**c**	xs:double	0	optional	
**fun.nr**	function.nrs		required	

Source

```
<xs:element name="contribution.function" type="functional.relationship" minOccurs="0" maxOccurs="1"/
>
```

Element female / age.dependent.contribution

Namespace	No namespace
Diagram	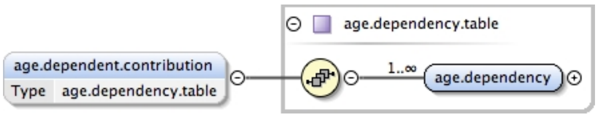
Type	age.dependency.table
Properties	content: complex; minOccurs: 0; maxOccurs: 1
Model	age.dependency+
Children	age.dependency

Instance

```
<age.dependent.contribution>
  <age.dependency age="" x="">{1,unbounded}</age.dependency>
</age.dependent.contribution>
```

Source

```
<xs:element name="age.dependent.contribution" type="age.dependency.table" minOccurs="0"
 maxOccurs="1"/>
```

Element female / contribution.index

Namespace	No namespace
Diagram	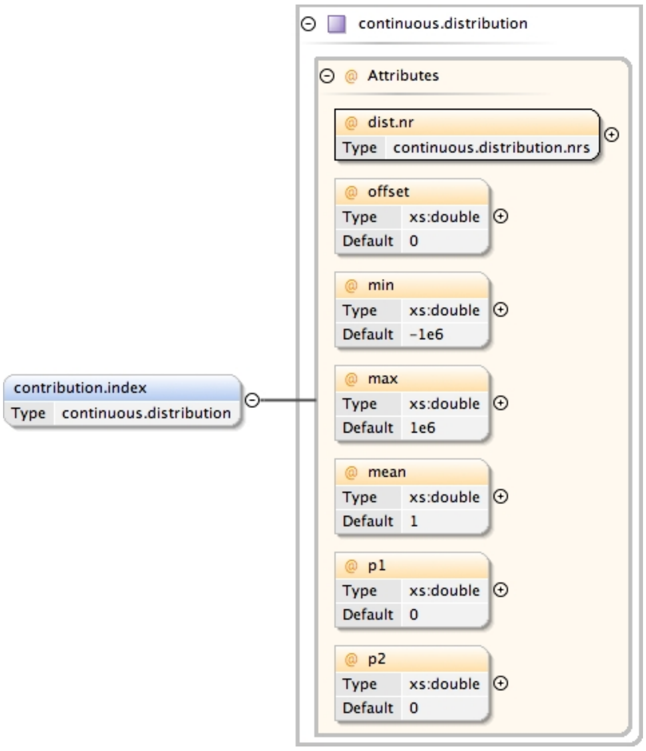

Type	continuous.distribution
Properties	content: `complex`<br>minOccurs: `0`<br>maxOccurs: `1`

Attributes

QName	Type	Default	Use	
**dist.nr**	continuous.distribution.nrs		required	
**max**	xs:double	1e6	optional	
**mean**	xs:double	1	optional	
**min**	xs:double	-1e6	optional	
**offset**	xs:double	0	optional	
**p1**	xs:double	0	optional	
**p2**	xs:double	0	optional	

Source

```
<xs:element name="contribution.index" type="continuous.distribution" minOccurs="0" maxOccurs="1"/>
```

Element `immunity`

Namespace	No namespace
Diagram	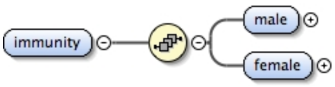
Properties	content: `complex`
Used by	Element wormsim.inputfile
Model	male , female
Children	female, male

Instance

```
<immunity>
  <male alpha="" beta="">{1,1}</male>
  <female alpha="" beta="">{1,1}</female>
</immunity>
```

Source

```
<xs:element name="immunity">
  <xs:complexType>
    <xs:sequence>
      <xs:element name="male">
        <xs:complexType>
          <xs:sequence>
            <xs:element ref="immunity.function"/>
            <xs:element ref="immunity.index"/>
          </xs:sequence>
          <xs:attribute name="alpha" use="required" type="nonnegdouble"/>
          <xs:attribute name="beta" use="required" type="nonnegdouble"/>
        </xs:complexType>
      </xs:element>
      <xs:element name="female">
        <xs:complexType>
          <xs:sequence>
            <xs:element ref="immunity.function"/>
            <xs:element ref="immunity.index"/>
          </xs:sequence>
          <xs:attribute name="alpha" use="required" type="nonnegdouble"/>
          <xs:attribute name="beta" use="required" type="nonnegdouble"/>
        </xs:complexType>
      </xs:element>
    </xs:sequence>
  </xs:complexType>
</xs:element>
```

Element `immunity / male`

Namespace	No namespace

<table>
<tr><td>Diagram</td><td>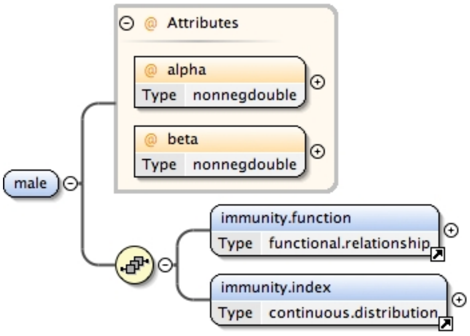
</td></tr>
<tr><td>Properties</td><td>content: complex</td></tr>
<tr><td>Model</td><td>immunity.function , immunity.index</td></tr>
<tr><td>Children</td><td>immunity.function, immunity.index</td></tr>
<tr><td>Instance</td><td>

```
<male alpha="" beta="">
  <immunity.function a="" b="0" c="0" fun.nr="">{1,1}</immunity.function>
  <immunity.index dist.nr="" max="1e6" mean="1" min="-1e6" offset="0" p1="0" p2="0">{1,1}</
immunity.index>
</male>
```

</td></tr>
<tr><td>Attributes</td><td>

QName	Type	Use	
**alpha**	nonnegdouble	required	
**beta**	nonnegdouble	required	

</td></tr>
<tr><td>Source</td><td>

```
<xs:element name="male">
  <xs:complexType>
    <xs:sequence>
      <xs:element ref="immunity.function"/>
      <xs:element ref="immunity.index"/>
    </xs:sequence>
    <xs:attribute name="alpha" use="required" type="nonnegdouble"/>
    <xs:attribute name="beta" use="required" type="nonnegdouble"/>
  </xs:complexType>
</xs:element>
```

</td></tr>
</table>

Element `immunity.function`

<table>
<tr><td>Namespace</td><td>No namespace</td></tr>
<tr><td>Diagram</td><td>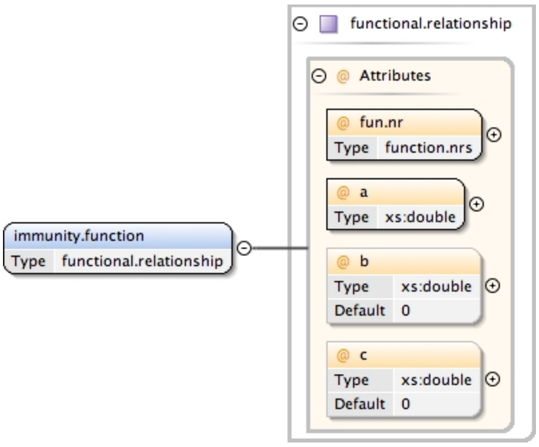
</td></tr>
<tr><td>Type</td><td>functional.relationship</td></tr>
<tr><td>Properties</td><td>content: complex</td></tr>
<tr><td>Used by</td><td>Elements immunity/female, immunity/male</td></tr>
<tr><td>Attributes</td><td>

QName	Type	Default	Use	
**a**	xs:double		required	
**b**	xs:double	0	optional	
**c**	xs:double	0	optional	
**fun.nr**	function.nrs		required	

</td></tr>
</table>

Source	`<xs:element name="immunity.function" type="functional.relationship"/>`

Element `immunity.index`

Namespace	No namespace
Diagram	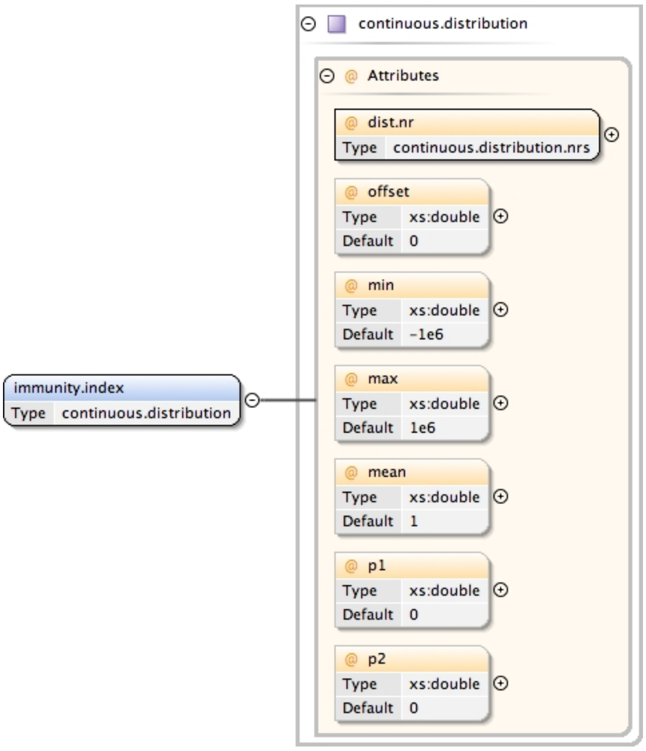
Type	continuous.distribution
Properties	content: `complex`
Used by	Elements immunity/female, immunity/male

Attributes

QName	Type	Default	Use	
**dist.nr**	continuous.distribution.nrs		required	
**max**	xs:double	1e6	optional	
**mean**	xs:double	1	optional	
**min**	xs:double	-1e6	optional	
**offset**	xs:double	0	optional	
**p1**	xs:double	0	optional	
**p2**	xs:double	0	optional	

Source	`<xs:element name="immunity.index" type="continuous.distribution"/>`

Element `immunity / female`

Namespace	No namespace
Diagram	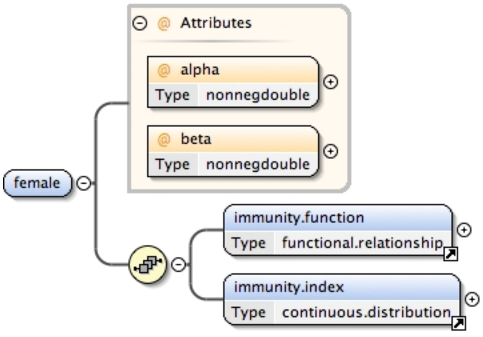

Properties	content: `complex`
Model	immunity.function , immunity.index
Children	immunity.function, immunity.index
Instance	`<female alpha="" beta="">`<br>`  <immunity.function a="" b="0" c="0" fun.nr="">{1,1}</immunity.function>`<br>`  <immunity.index dist.nr="" max="1e6" mean="1" min="-1e6" offset="0" p1="0" p2="0">{1,1}</`<br>`immunity.index>`<br>`</female>`
Attributes	**QName** / **Type** / **Use**<br>**alpha** / nonnegdouble / required<br>**beta** / nonnegdouble / required
Source	`<xs:element name="female">`<br>`  <xs:complexType>`<br>`    <xs:sequence>`<br>`      <xs:element ref="immunity.function"/>`<br>`      <xs:element ref="immunity.index"/>`<br>`    </xs:sequence>`<br>`    <xs:attribute name="alpha" use="required" type="nonnegdouble"/>`<br>`    <xs:attribute name="beta" use="required" type="nonnegdouble"/>`<br>`  </xs:complexType>`<br>`</xs:element>`

Element worm

Namespace	No namespace
Diagram	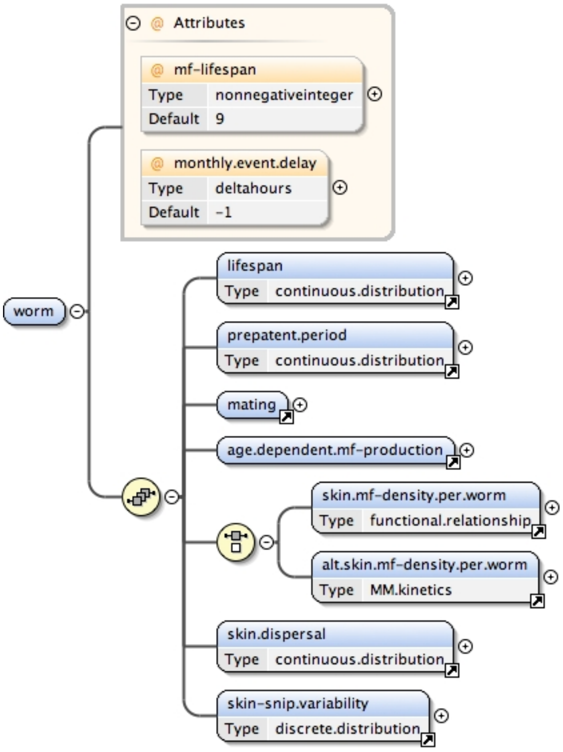
Properties	content: `complex`
Used by	Element wormsim.inputfile
Model	lifespan , prepatent.period , mating , age.dependent.mf-production , (skin.mf-density.per.worm | alt.skin.mf-density.per.worm) , skin.dispersal , skin-snip.variability
Children	age.dependent.mf-production, alt.skin.mf-density.per.worm, lifespan, mating, prepatent.period, skin-snip.variability, skin.dispersal, skin.mf-density.per.worm
Instance	`<worm mf-lifespan="9" monthly.event.delay="-1">`<br>`  <lifespan dist.nr="" max="1e6" mean="1" min="-1e6" offset="0" p1="0" p2="0">{1,1}</lifespan>`<br>`  <prepatent.period dist.nr="" max="1e6" mean="1" min="-1e6" offset="0" p1="0" p2="0">{1,1}</`<br>`prepatent.period>`<br>`  <mating cycle="" male.potential="" sex.ratio="1">{1,1}</mating>`<br>`  <age.dependent.mf-production labda="0.0">{1,1}</age.dependent.mf-production>`

	```<br><skin.mf-density.per.worm a="" b="0" c="0" fun.nr="">{1,1}</skin.mf-density.per.worm><br><alt.skin.mf-density.per.worm>{1,1}</alt.skin.mf-density.per.worm><br><skin.dispersal dist.nr="" max="1e6" mean="1" min="-1e6" offset="0" p1="0" p2="0">{1,1}</<br>skin.dispersal><br><skin-snip.variability dist.nr="" mean="1" p1="0" p2="0">{1,1}</skin-snip.variability><br></worm><br>```

Attributes	QName	Type	Default	Use
	**mf-lifespan**	nonnegativeinteger	9	optional
	**monthly.event.delay**	deltahours	-1	optional

Source

```
<xs:element name="worm">
  <xs:complexType>
    <xs:sequence>
      <xs:element ref="lifespan"/>
      <xs:element ref="prepatent.period"/>
      <xs:element ref="mating"/>
      <xs:element ref="age.dependent.mf-production"/>
      <xs:choice>
        <xs:element ref="skin.mf-density.per.worm"/>
        <xs:element ref="alt.skin.mf-density.per.worm"/>
      </xs:choice>
      <xs:element ref="skin.dispersal"/>
      <xs:element ref="skin-snip.variability"/>
    </xs:sequence>
    <xs:attribute name="mf-lifespan" use="optional" type="nonnegativeinteger" default="9"/>
    <xs:attribute name="monthly.event.delay" use="optional" type="deltahours" default="-1"/>
  </xs:complexType>
</xs:element>
```

Element `lifespan`

Namespace	No namespace
Diagram	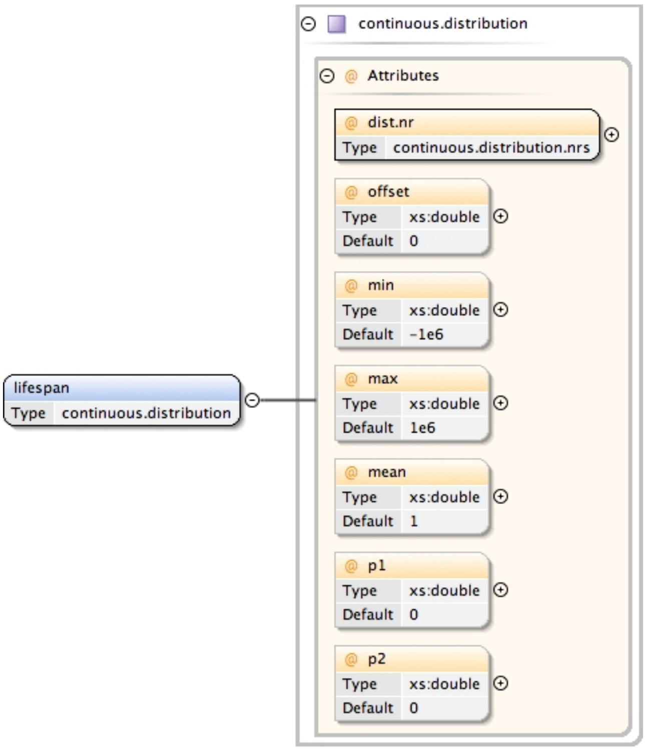
Type	continuous.distribution
Properties	content: complex
Used by	Element worm

Attributes	QName	Type	Default	Use
	**dist.nr**	continuous.distribution.nrs		required
	**max**	xs:double	1e6	optional
	**mean**	xs:double	1	optional

	QName	Type	Default	Use
	**min**	xs:double	-1e6	optional
	**offset**	xs:double	0	optional
	**p1**	xs:double	0	optional
	**p2**	xs:double	0	optional
Source	`<xs:element name="lifespan" type="continuous.distribution"/>`			

Element `prepatent.period`

Namespace	No namespace
Diagram	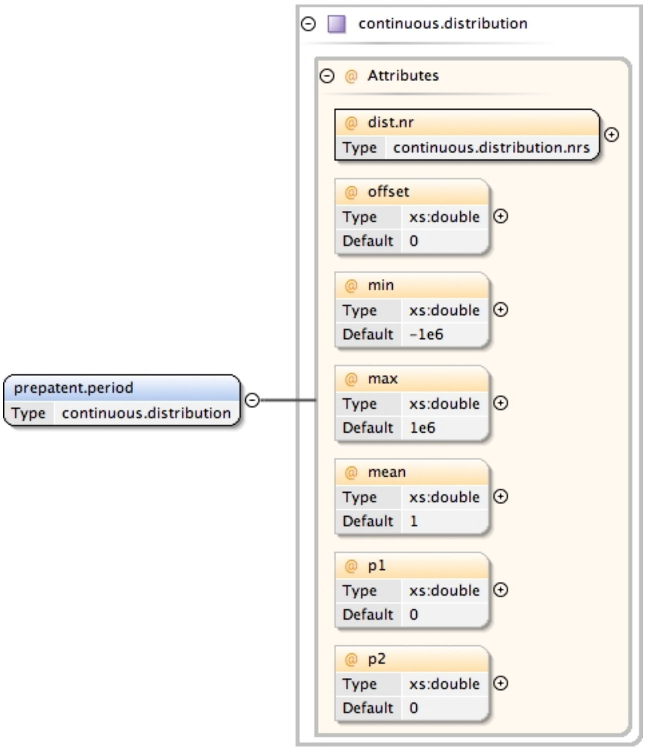
Type	continuous.distribution
Properties	content: `complex`
Used by	Element worm

Attributes

QName	Type	Default	Use
**dist.nr**	continuous.distribution.nrs		required
**max**	xs:double	1e6	optional
**mean**	xs:double	1	optional
**min**	xs:double	-1e6	optional
**offset**	xs:double	0	optional
**p1**	xs:double	0	optional
**p2**	xs:double	0	optional

Source: `<xs:element name="prepatent.period" type="continuous.distribution"/>`

Element `mating`

Namespace	No namespace

Diagram

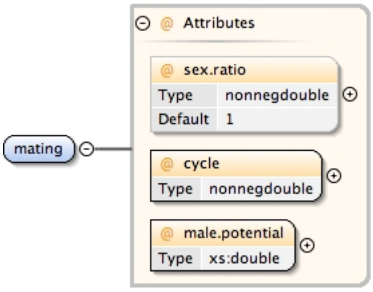

Properties	content:	complex
Used by	Element	worm

Attributes

QName	Type	Default	Use	
**cycle**	nonnegdouble		required	
**male.potential**	xs:double		required	
**sex.ratio**	nonnegdouble	1	optional	

Source

```
<xs:element name="mating">
  <xs:complexType>
    <xs:attribute name="sex.ratio" use="optional" type="nonnegdouble" default="1"/>
    <xs:attribute name="cycle" use="required" type="nonnegdouble"/>
    <xs:attribute name="male.potential" use="required" type="xs:double"/>
  </xs:complexType>
</xs:element>
```

Element `age.dependent.mf-production`

Namespace	No namespace

Diagram

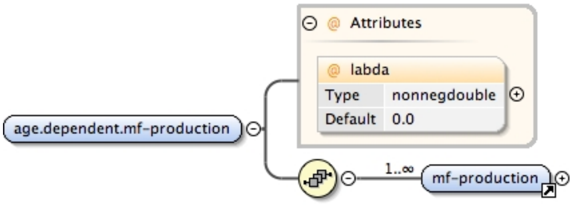

Properties	content:	complex
Used by	Element	worm
Model	mf-production+	
Children	mf-production	

Instance

```
<age.dependent.mf-production labda="0.0">
  <mf-production age.limit="" production="">{1,unbounded}</mf-production>
</age.dependent.mf-production>
```

Attributes

QName	Type	Default	Use	
**labda**	nonnegdouble	0.0	optional	

Source

```
<xs:element name="age.dependent.mf-production">
  <xs:complexType>
    <xs:sequence>
      <xs:element maxOccurs="unbounded" ref="mf-production"/>
    </xs:sequence>
    <xs:attribute name="labda" default="0.0" type="nonnegdouble"/>
  </xs:complexType>
</xs:element>
```

Element `mf-production`

Namespace	No namespace

Diagram

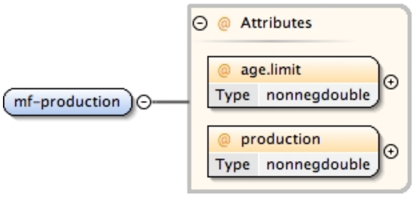

Properties	content:	`complex`
Used by	Element	age.dependent.mf-production

Attributes

QName	Type	Use	
**age.limit**	nonnegdouble	required	
**production**	nonnegdouble	required	

Source

```
<xs:element name="mf-production">
  <xs:complexType>
    <xs:attribute name="age.limit" use="required" type="nonnegdouble"/>
    <xs:attribute name="production" use="required" type="nonnegdouble"/>
  </xs:complexType>
</xs:element>
```

Element `skin.mf-density.per.worm`

Namespace	No namespace

Diagram

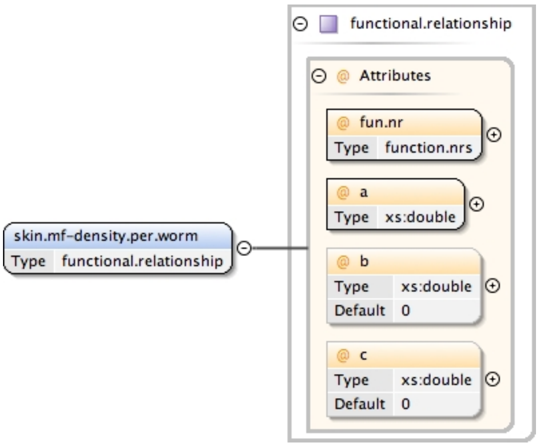

Type	functional.relationship	
Properties	content:	`complex`
Used by	Element	worm

Attributes

QName	Type	Default	Use	
**a**	xs:double		required	
**b**	xs:double	0	optional	
**c**	xs:double	0	optional	
**fun.nr**	function.nrs		required	

Source

```
<xs:element name="skin.mf-density.per.worm" type="functional.relationship"/>
```

Element `alt.skin.mf-density.per.worm`

Namespace	No namespace

Diagram	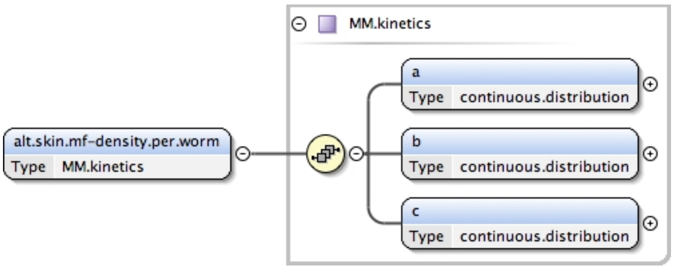
Type	MM.kinetics
Properties	content: complex
Used by	Element worm
Model	a , b , c
Children	a, b, c
Instance	`<alt.skin.mf-density.per.worm>`<br>`<a dist.nr="" max="1e6" mean="1" min="-1e6" offset="0" p1="0" p2="0">{1,1}</a>`<br>`<b dist.nr="" max="1e6" mean="1" min="-1e6" offset="0" p1="0" p2="0">{1,1}</b>`<br>`<c dist.nr="" max="1e6" mean="1" min="-1e6" offset="0" p1="0" p2="0">{1,1}</c>`<br>`</alt.skin.mf-density.per.worm>`
Source	`<xs:element name="alt.skin.mf-density.per.worm" type="MM.kinetics"/>`

Element `skin.dispersal`

Namespace	No namespace
Diagram	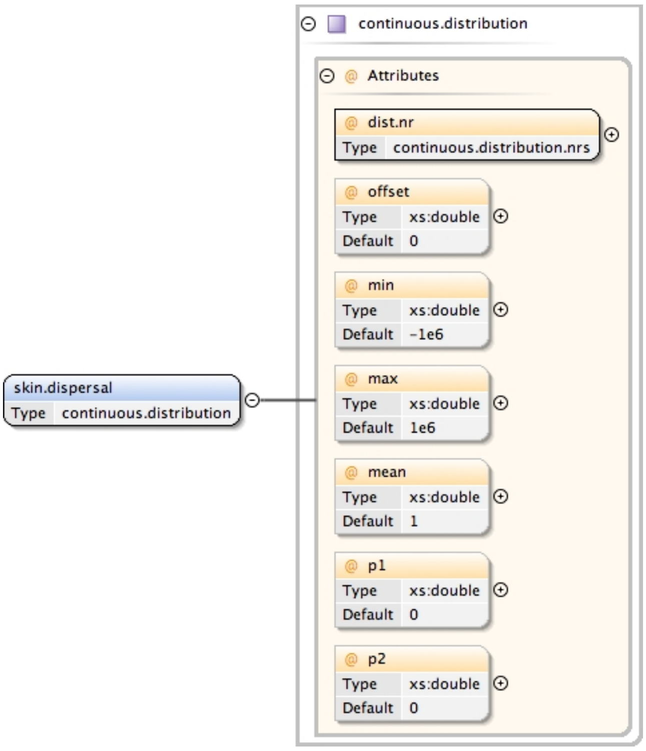
Type	continuous.distribution
Properties	content: complex
Used by	Element worm

Attributes

QName	Type	Default	Use	
**dist.nr**	continuous.distribution.nrs		required	
**max**	xs:double	1e6	optional	
**mean**	xs:double	1	optional	
**min**	xs:double	-1e6	optional	

QName	Type	Default	Use
**offset**	xs:double	0	optional
**p1**	xs:double	0	optional
**p2**	xs:double	0	optional

Source	`<xs:element name="skin.dispersal" type="continuous.distribution"/>`

Element `skin-snip.variability`

Namespace	No namespace
Diagram	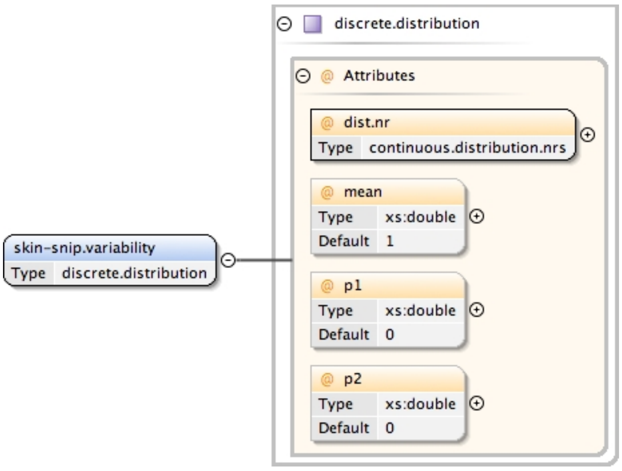
Type	discrete.distribution
Properties	content: `complex`
Used by	Element worm

Attributes

QName	Type	Default	Use
**dist.nr**	continuous.distribution.nrs		required
**mean**	xs:double	1	optional
**p1**	xs:double	0	optional
**p2**	xs:double	0	optional

Source	`<xs:element name="skin-snip.variability" type="discrete.distribution"/>`

Element `fly`

Namespace	No namespace
Diagram	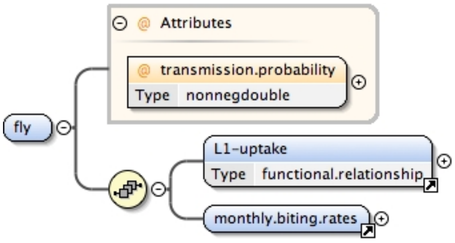
Properties	content: `complex`
Used by	Element wormsim.inputfile
Model	L1-uptake , monthly.biting.rates
Children	L1-uptake, monthly.biting.rates

Instance

```
<fly transmission.probability="">
  <L1-uptake a="" b="0" c="0" fun.nr="">{1,1}</L1-uptake>
  <monthly.biting.rates relative.biting.rate="">{1,1}</monthly.biting.rates>
</fly>
```

Attributes

QName	Type	Use	
**transmission.probability**	nonnegdouble	required	

Source

```
<xs:element name="fly">
  <xs:complexType>
    <xs:sequence>
      <xs:element ref="L1-uptake"/>
      <xs:element ref="monthly.biting.rates"/>
    </xs:sequence>
    <xs:attribute name="transmission.probability" use="required" type="nonnegdouble"/>
  </xs:complexType>
</xs:element>
```

Element `L1-uptake`

Namespace	No namespace
Diagram	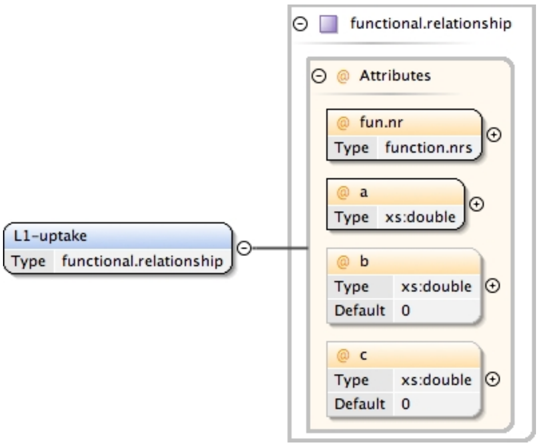
Type	functional.relationship
Properties	content: complex
Used by	Element fly

Attributes

QName	Type	Default	Use	
**a**	xs:double		required	
**b**	xs:double	0	optional	
**c**	xs:double	0	optional	
**fun.nr**	function.nrs		required	

Source

```
<xs:element name="L1-uptake" type="functional.relationship"/>
```

Element `monthly.biting.rates`

Namespace	No namespace
Diagram	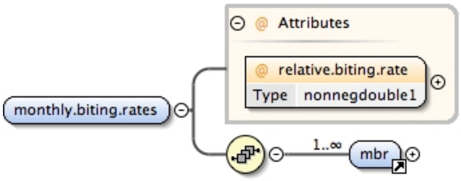
Properties	content: complex
Used by	Element fly
Model	mbr+
Children	mbr

Instance

```
<monthly.biting.rates relative.biting.rate="">
  <mbr month="" rate="">{1,unbounded}</mbr>
</monthly.biting.rates>
```

Attributes	**QName**	**Type**	**Use**	
	**relative.biting.rate**	nonnegdouble1	required	

Source
```
<xs:element name="monthly.biting.rates">
  <xs:complexType>
    <xs:sequence>
      <xs:element maxOccurs="unbounded" ref="mbr"/>
    </xs:sequence>
    <xs:attribute name="relative.biting.rate" use="required" type="nonnegdouble1"/>
  </xs:complexType>
</xs:element>
```

Element mbr

Namespace	No namespace
Diagram	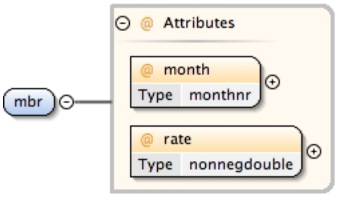
Properties	content: complex
Used by	Elements monthly.birth.rates, monthly.biting.rates

Attributes	**QName**	**Type**	**Use**
	**month**	monthnr	required
	**rate**	nonnegdouble	required

Source
```
<xs:element name="mbr">
  <xs:complexType>
    <xs:attribute name="month" use="required" type="monthnr"/>
    <xs:attribute name="rate" use="required" type="nonnegdouble"/>
  </xs:complexType>
</xs:element>
```

Element snails

Namespace	No namespace
Diagram	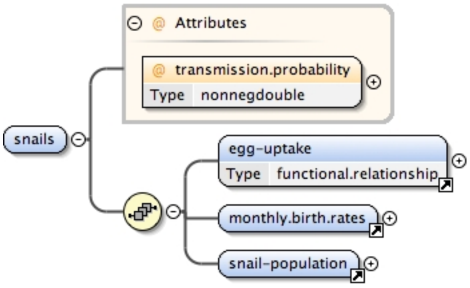
Properties	content: complex
Used by	Element wormsim.inputfile
Model	egg-uptake , monthly.birth.rates , snail-population
Children	egg-uptake, monthly.birth.rates, snail-population

Instance
```
<snails transmission.probability="">
  <egg-uptake a="" b="0" c="0" fun.nr="">{1,1}</egg-uptake>
  <monthly.birth.rates relative.birth.rate="">{1,1}</monthly.birth.rates>
  <snail-population>{1,1}</snail-population>
</snails>
```

Attributes	**QName**	**Type**	**Use**
	**transmission.probability**	nonnegdouble	required

Source
```
<xs:element name="snails">
  <xs:complexType>
    <xs:sequence>
```

```
      <xs:element ref="egg-uptake"/>
      <xs:element ref="monthly.birth.rates"/>
      <xs:element ref="snail-population"/>
    </xs:sequence>
    <xs:attribute name="transmission.probability" use="required" type="nonnegdouble"/>
  </xs:complexType>
</xs:element>
```

Element egg-uptake

Namespace	No namespace
Diagram	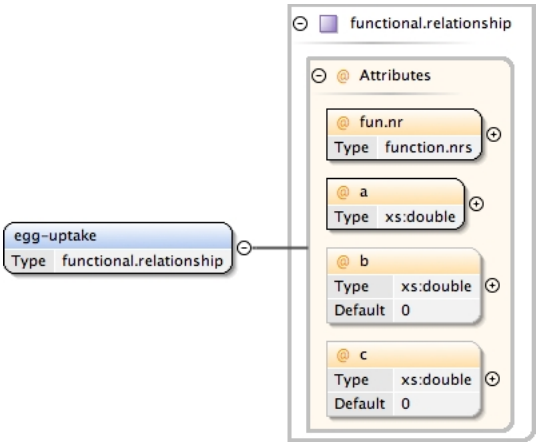

Type	functional.relationship
Properties	content: complex
Used by	Element snails

Attributes

QName	Type	Default	Use	
**a**	xs:double		required	
**b**	xs:double	0	optional	
**c**	xs:double	0	optional	
**fun.nr**	function.nrs		required	

Source

```
<xs:element name="egg-uptake" type="functional.relationship"/>
```

Element monthly.birth.rates

Namespace	No namespace
Diagram	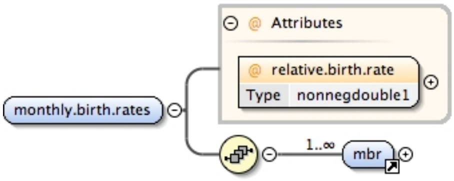

Properties	content: complex
Used by	Element snails
Model	mbr+
Children	mbr

Instance

```
<monthly.birth.rates relative.birth.rate="">
  <mbr month="" rate="">{1,unbounded}</mbr>
</monthly.birth.rates>
```

Attributes

QName	Type	Use	
**relative.birth.rate**	nonnegdouble1	required	

Source

```
<xs:element name="monthly.birth.rates">
  <xs:complexType>
    <xs:sequence>
      <xs:element maxOccurs="unbounded" ref="mbr"/>
```

```
    </xs:sequence>
    <xs:attribute name="relative.birth.rate" use="required" type="nonnegdouble1"/>
  </xs:complexType>
</xs:element>
```

Element snail-population

Namespace	No namespace
Diagram	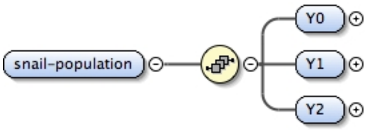
Properties	content: complex
Used by	Element snails
Model	Y0 , Y1 , Y2
Children	Y0, Y1, Y2

Instance

```
<snail-population>
  <Y0 initial="" mu="">{1,1}</Y0>
  <Y1 initial="" mu="" sigma="">{1,1}</Y1>
  <Y2 initial="" mu="">{1,1}</Y2>
</snail-population>
```

Source

```
<xs:element name="snail-population">
  <xs:complexType>
    <xs:sequence>
      <xs:element name="Y0">
        <xs:complexType>
          <xs:attribute name="initial" use="required" type="nonnegdouble"/>
          <xs:attribute name="mu" use="required" type="nonnegdouble"/>
        </xs:complexType>
      </xs:element>
      <xs:element name="Y1">
        <xs:complexType>
          <xs:attribute name="initial" use="required" type="nonnegdouble"/>
          <xs:attribute name="mu" use="required" type="nonnegdouble"/>
          <xs:attribute name="sigma" use="required" type="nonnegdouble"/>
        </xs:complexType>
      </xs:element>
      <xs:element name="Y2">
        <xs:complexType>
          <xs:attribute name="initial" use="required" type="nonnegdouble"/>
          <xs:attribute name="mu" use="required" type="nonnegdouble"/>
        </xs:complexType>
      </xs:element>
    </xs:sequence>
  </xs:complexType>
</xs:element>
```

Element snail-population / Y0

Namespace	No namespace
Diagram	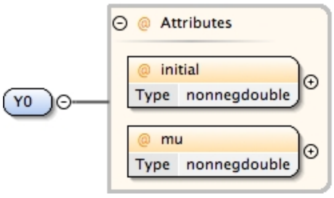
Properties	content: complex

Attributes

QName	Type	Use
**initial**	nonnegdouble	required
**mu**	nonnegdouble	required

Source

```
<xs:element name="Y0">
  <xs:complexType>
    <xs:attribute name="initial" use="required" type="nonnegdouble"/>
    <xs:attribute name="mu" use="required" type="nonnegdouble"/>
  </xs:complexType>
```

```
</xs:element>
```

Element snail-population / Y1

Namespace	No namespace
Diagram	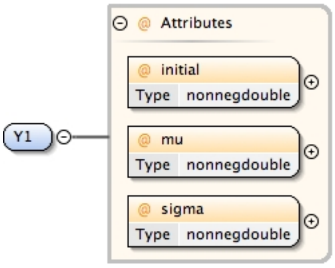
Properties	content: complex

Attributes

QName	Type	Use	
**initial**	nonnegdouble	required	
**mu**	nonnegdouble	required	
**sigma**	nonnegdouble	required	

Source

```
<xs:element name="Y1">
  <xs:complexType>
    <xs:attribute name="initial" use="required" type="nonnegdouble"/>
    <xs:attribute name="mu" use="required" type="nonnegdouble"/>
    <xs:attribute name="sigma" use="required" type="nonnegdouble"/>
  </xs:complexType>
</xs:element>
```

Element snail-population / Y2

Namespace	No namespace
Diagram	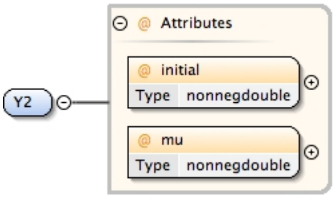
Properties	content: complex

Attributes

QName	Type	Use	
**initial**	nonnegdouble	required	
**mu**	nonnegdouble	required	

Source

```
<xs:element name="Y2">
  <xs:complexType>
    <xs:attribute name="initial" use="required" type="nonnegdouble"/>
    <xs:attribute name="mu" use="required" type="nonnegdouble"/>
  </xs:complexType>
</xs:element>
```

Element mass.treatment

Namespace	No namespace
Diagram	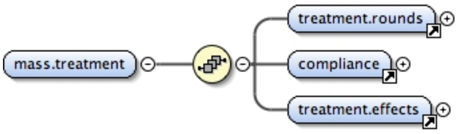
Properties	content: complex
Used by	Element wormsim.inputfile
Model	treatment.rounds , compliance , treatment.effects

Children	compliance, treatment.effects, treatment.rounds
Instance	<mass.treatment><br><treatment.rounds>{1,1}</treatment.rounds><br><compliance compliance.model="0" fraction.excluded="" fraction.malabsorption="" test.first="false">{1,1}</compliance><br><treatment.effects fraction.killed="" period.of.recovery="" permanent.reduction.mf-production="" shape.parameter.recovery.function="">{1,1}</treatment.effects><br></mass.treatment>
Source	<xs:element name="mass.treatment"><br><xs:complexType><br><xs:sequence><br><xs:element ref="treatment.rounds"/><br><xs:element ref="compliance"/><br><xs:element ref="treatment.effects"/><br></xs:sequence><br></xs:complexType><br></xs:element>

Element `treatment.rounds`

Namespace	No namespace
Diagram	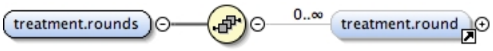
Properties	content: complex
Used by	Element mass.treatment
Model	treatment.round*
Children	treatment.round
Instance	<treatment.rounds><br><treatment.round coverage="" delay="-4" month="" year="">{0,unbounded}</treatment.round><br></treatment.rounds>
Source	<xs:element name="treatment.rounds"><br><xs:complexType><br><xs:sequence><br><xs:element minOccurs="0" maxOccurs="unbounded" ref="treatment.round"/><br></xs:sequence><br></xs:complexType><br></xs:element>

Element `treatment.round`

Namespace	No namespace
Diagram	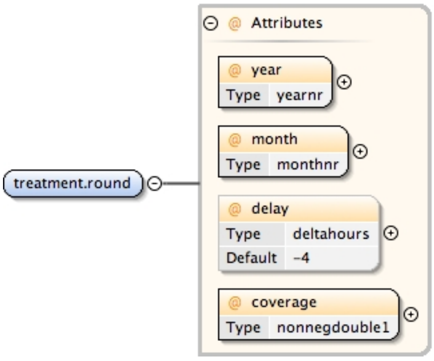
Properties	content: complex
Used by	Element treatment.rounds
Attributes	**QName** / **Type** / **Default** / **Use**<br>**coverage** / nonnegdouble1 / / required<br>**delay** / deltahours / -4 / optional<br>**month** / monthnr / / required<br>**year** / yearnr / / required
Source	<xs:element name="treatment.round">

```
    <xs:complexType>
      <xs:attribute name="year" use="required" type="yearnr"/>
      <xs:attribute name="month" use="required" type="monthnr"/>
      <xs:attribute name="delay" use="optional" type="deltahours" default="-4"/>
      <xs:attribute name="coverage" use="required" type="nonnegdouble1"/>
    </xs:complexType>
  </xs:element>
```

Element compliance

Namespace	No namespace

Diagram

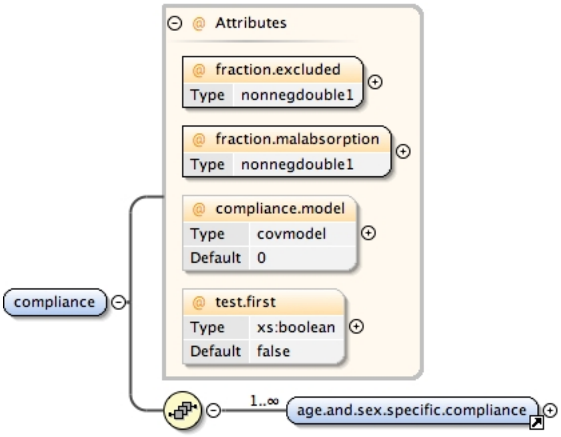

Properties	content:	complex
Used by	Element	mass.treatment
Model	age.and.sex.specific.compliance+	
Children	age.and.sex.specific.compliance	

Instance

```
<compliance compliance.model="0" fraction.excluded="" fraction.malabsorption="" test.first="false">
  <age.and.sex.specific.compliance age.limit="" female.compliance="" male.compliance="">{1,unbounded}</
age.and.sex.specific.compliance>
</compliance>
```

Attributes

QName	Type	Default	Use	
**compliance.model**	covmodel	0	optional	
**fraction.excluded**	nonnegdouble1		required	
**fraction.malabsorption**	nonnegdouble1		required	
**test.first**	xs:boolean	false	optional	

Source

```
<xs:element name="compliance">
  <xs:complexType>
    <xs:sequence>
      <xs:element maxOccurs="unbounded" ref="age.and.sex.specific.compliance"/>
    </xs:sequence>
    <xs:attribute name="fraction.excluded" use="required" type="nonnegdouble1"/>
    <xs:attribute name="fraction.malabsorption" use="required" type="nonnegdouble1"/>
    <xs:attribute name="compliance.model" default="0" type="covmodel"/>
    <xs:attribute name="test.first" default="false" type="xs:boolean"/>
  </xs:complexType>
</xs:element>
```

Element age.and.sex.specific.compliance

Namespace	No namespace

Diagram

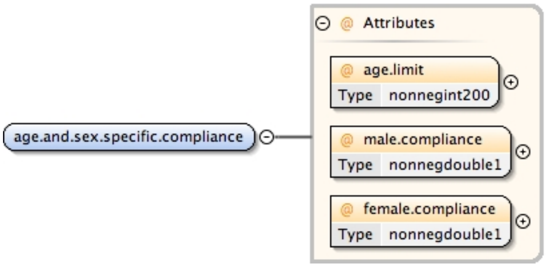

Properties	content: `complex`
Used by	Element compliance

Attributes

QName	Type	Use	
**age.limit**	nonnegint200	required	
**female.compliance**	nonnegdouble1	required	
**male.compliance**	nonnegdouble1	required	

Source

```
<xs:element name="age.and.sex.specific.compliance">
  <xs:complexType>
    <xs:attribute name="age.limit" use="required" type="nonnegint200"/>
    <xs:attribute name="male.compliance" use="required" type="nonnegdouble1"/>
    <xs:attribute name="female.compliance" use="required" type="nonnegdouble1"/>
  </xs:complexType>
</xs:element>
```

Element `treatment.effects`

Namespace	No namespace

Diagram

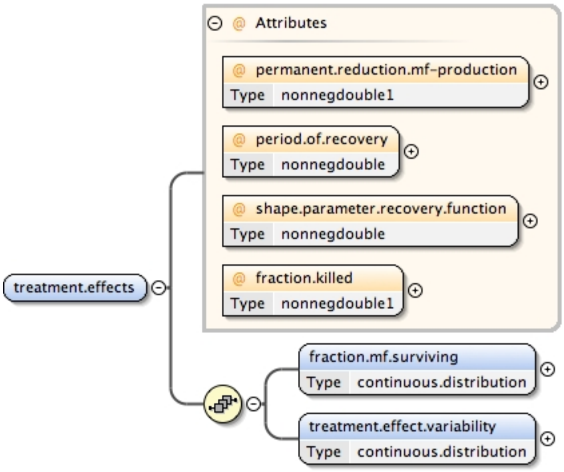

Properties	content: `complex`
Used by	Element mass.treatment
Model	fraction.mf.surviving , treatment.effect.variability
Children	fraction.mf.surviving, treatment.effect.variability

Instance

```
<treatment.effects fraction.killed="" period.of.recovery="" permanent.reduction.mf-
production="" shape.parameter.recovery.function="">
  <fraction.mf.surviving dist.nr="" max="1e6" mean="1" min="-1e6" offset="0" p1="0" p2="0">{1,1}</
fraction.mf.surviving>
  <treatment.effect.variability dist.nr="" max="1e6" mean="1" min="-1e6" offset="0" p1="0" p2="0">{1,1}</
treatment.effect.variability>
</treatment.effects>
```

Attributes

QName	Type	Use	
**fraction.killed**	nonnegdouble1	required	
**period.of.recovery**	nonnegdouble	required	
**permanent.reduction.mf-production**	nonnegdouble1	required	
**shape.parameter.recovery.function**	nonnegdouble	required	

Source

```
<xs:element name="treatment.effects">
  <xs:complexType>
    <xs:sequence>
      <xs:element name="fraction.mf.surviving" type="continuous.distribution"/>
      <xs:element name="treatment.effect.variability" type="continuous.distribution"/>
    </xs:sequence>
    <xs:attribute name="permanent.reduction.mf-production" use="required" type="nonnegdouble1"/>
    <xs:attribute name="period.of.recovery" use="required" type="nonnegdouble"/>
    <xs:attribute name="shape.parameter.recovery.function" use="required" type="nonnegdouble"/>
    <xs:attribute name="fraction.killed" use="required" type="nonnegdouble1"/>
  </xs:complexType>
```

	`</xs:element>`

Element treatment.effects / fraction.mf.surviving

Namespace	No namespace
Diagram	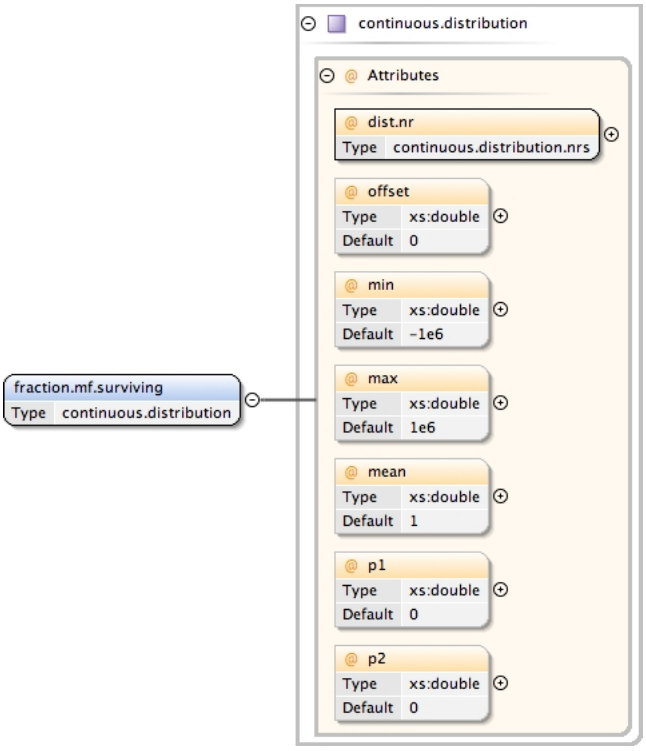
Type	continuous.distribution
Properties	content: complex

Attributes

QName	Type	Default	Use	
**dist.nr**	continuous.distribution.nrs		required	
**max**	xs:double	1e6	optional	
**mean**	xs:double	1	optional	
**min**	xs:double	-1e6	optional	
**offset**	xs:double	0	optional	
**p1**	xs:double	0	optional	
**p2**	xs:double	0	optional	

Source

```
<xs:element name="fraction.mf.surviving" type="continuous.distribution"/>
```

Element treatment.effects / treatment.effect.variability

Namespace	No namespace

Diagram	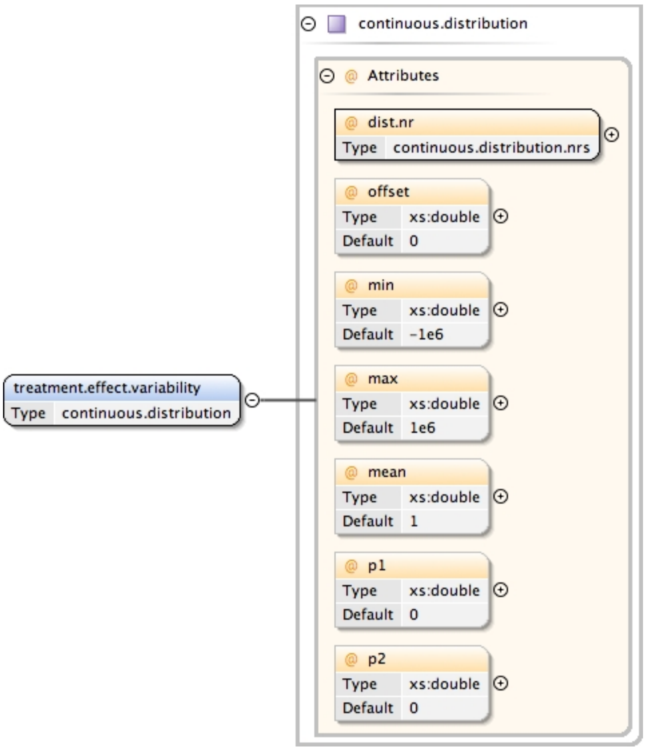
Type	continuous.distribution
Properties	content: complex

Attributes

QName	Type	Default	Use	
**dist.nr**	continuous.distribution.nrs		required	
**max**	xs:double	1e6	optional	
**mean**	xs:double	1	optional	
**min**	xs:double	-1e6	optional	
**offset**	xs:double	0	optional	
**p1**	xs:double	0	optional	
**p2**	xs:double	0	optional	

Source

```
<xs:element name="treatment.effect.variability" type="continuous.distribution"/>
```

Element `vector.control`

Namespace	No namespace
Diagram	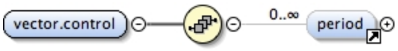
Properties	content: complex
Used by	Element wormsim.inputfile
Model	period*
Children	period

Instance

```
<vector.control>
  <period effectivity="" start.month="0" start.year="" stop.month="0" stop.year="">{0,unbounded}</
period>
</vector.control>
```

Source

```
<xs:element name="vector.control">
  <xs:complexType>
    <xs:sequence>
      <xs:element minOccurs="0" maxOccurs="unbounded" ref="period"/>
    </xs:sequence>
  </xs:complexType>
</xs:element>
```

Element period

Namespace	No namespace

Diagram

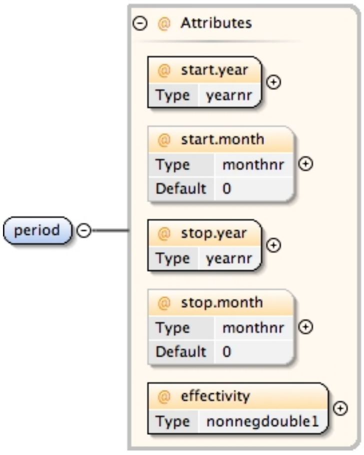

Properties	content:	complex
Used by	Element	vector.control

Attributes

QName	Type	Default	Use	
**effectivity**	nonnegdouble1		required	
**start.month**	monthnr	0	optional	
**start.year**	yearnr		required	
**stop.month**	monthnr	0	optional	
**stop.year**	yearnr		required	

Source

```
<xs:element name="period">
  <xs:complexType>
    <xs:attribute name="start.year" use="required" type="yearnr"/>
    <xs:attribute name="start.month" default="0" type="monthnr"/>
    <xs:attribute name="stop.year" use="required" type="yearnr"/>
    <xs:attribute name="stop.month" default="0" type="monthnr"/>
    <xs:attribute name="effectivity" use="required" type="nonnegdouble1"/>
  </xs:complexType>
</xs:element>
```

Complex Type(s)

Complex Type age.table

Namespace	No namespace

Diagram

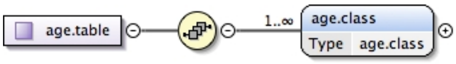

Used by	Element	surveillance/age.classes
Model	age.class+	
Children	age.class	

Source

```
<xs:complexType name="age.table">
  <xs:sequence>
    <xs:element maxOccurs="unbounded" name="age.class" type="age.class"/>
  </xs:sequence>
</xs:complexType>
```

Complex Type age.class

Namespace	No namespace

Diagram

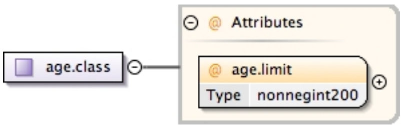

Used by	Element	age.table/age.class

Attributes

QName	Type	Use
**age.limit**	nonnegint200	required

Source

```
<xs:complexType name="age.class">
  <xs:attribute name="age.limit" use="required" type="nonnegint200"/>
</xs:complexType>
```

Complex Type population.table

Namespace	No namespace
Diagram	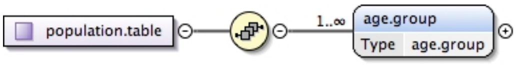

Used by	Elements	demography/initial.population, simulation/standard.population
Model	age.group+	
Children	age.group	

Source

```
<xs:complexType name="population.table">
  <xs:sequence>
    <xs:element maxOccurs="unbounded" name="age.group" type="age.group"/>
  </xs:sequence>
</xs:complexType>
```

Complex Type age.group

Namespace	No namespace
Diagram	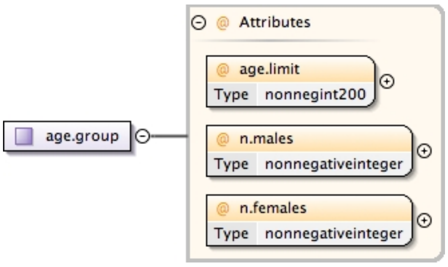

Used by	Element	population.table/age.group

Attributes

QName	Type	Use
**age.limit**	nonnegint200	required
**n.females**	nonnegativeinteger	required
**n.males**	nonnegativeinteger	required

Source

```
<xs:complexType name="age.group">
  <xs:attribute name="age.limit" use="required" type="nonnegint200"/>
  <xs:attribute name="n.males" use="required" type="nonnegativeinteger"/>
  <xs:attribute name="n.females" use="required" type="nonnegativeinteger"/>
</xs:complexType>
```

Complex Type functional.relationship

Namespace	No namespace
Diagram	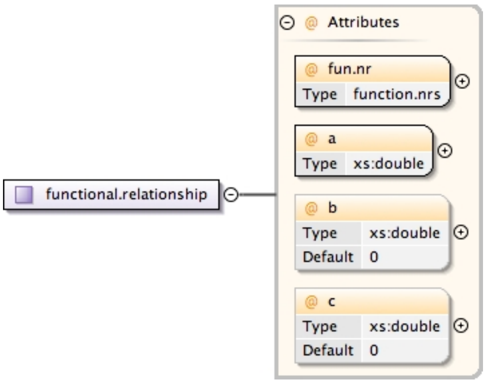

Used by	Elements	L1-uptake, egg-uptake, female/contribution.function, female/exposure.function, immunity.function, male/contribution.function, male/exposure.function, skin.mf-density.per.worm

Attributes

QName	Type	Default	Use	
**a**	xs:double		required	
**b**	xs:double	0	optional	
**c**	xs:double	0	optional	
**fun.nr**	function.nrs		required	

Source

```
<xs:complexType name="functional.relationship">
  <xs:attribute name="fun.nr" use="required" type="function.nrs"/>
  <xs:attribute name="a" use="required" type="xs:double"/>
  <xs:attribute name="b" use="optional" type="xs:double" default="0"/>
  <xs:attribute name="c" use="optional" type="xs:double" default="0"/>
</xs:complexType>
```

Complex Type `MM.kinetics`

Namespace	No namespace
Diagram	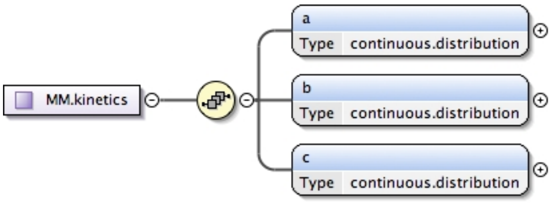

Used by	Element	alt.skin.mf-density.per.worm
Model	a , b , c	
Children	a, b, c	

Source

```
<xs:complexType name="MM.kinetics">
  <xs:sequence>
    <xs:element name="a" type="continuous.distribution"/>
    <xs:element name="b" type="continuous.distribution"/>
    <xs:element name="c" type="continuous.distribution"/>
  </xs:sequence>
</xs:complexType>
```

Complex Type `continuous.distribution`

Namespace	No namespace
Diagram	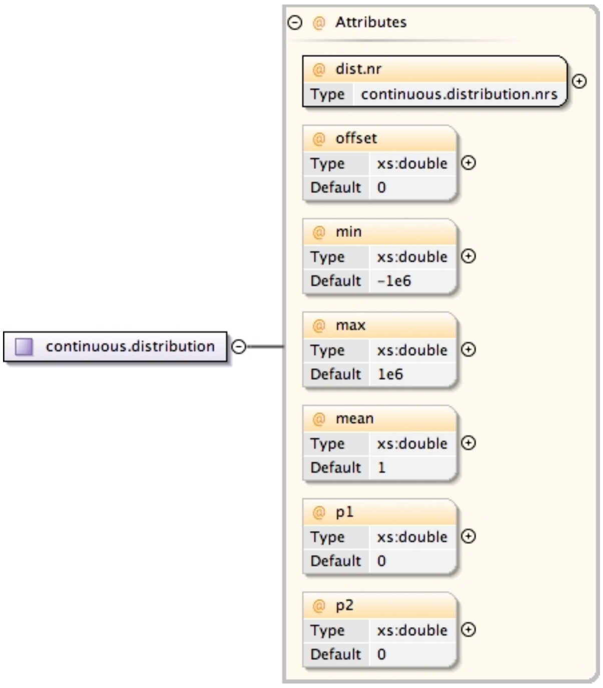

Used by	Elements	MM.kinetics/a, MM.kinetics/b, MM.kinetics/c, female/contribution.index, female/exposure.index, immunity.index, lifespan, male/contribution.index, male/exposure.index, pct-life-expectancy-reduction, prepatent.period, skin.dispersal, treatment.effects/fraction.mf.surviving, treatment.effects/treatment.effect.variability, treshold

Attributes

QName	Type	Default	Use	
**dist.nr**	continuous.distribution.nrs		required	
**max**	xs:double	1e6	optional	
**mean**	xs:double	1	optional	
**min**	xs:double	-1e6	optional	
**offset**	xs:double	0	optional	
**p1**	xs:double	0	optional	
**p2**	xs:double	0	optional	

Source

```
<xs:complexType name="continuous.distribution">
  <xs:attribute name="dist.nr" use="required" type="continuous.distribution.nrs"/>
  <xs:attribute name="offset" use="optional" type="xs:double" default="0"/>
  <xs:attribute name="min" use="optional" type="xs:double" default="-1e6"/>
  <xs:attribute name="max" use="optional" type="xs:double" default="1e6"/>
  <xs:attribute name="mean" use="optional" type="xs:double" default="1"/>
  <xs:attribute name="p1" use="optional" type="xs:double" default="0"/>
  <xs:attribute name="p2" use="optional" type="xs:double" default="0"/>
</xs:complexType>
```

Complex Type `discrete.distribution`

Namespace	No namespace

Diagram

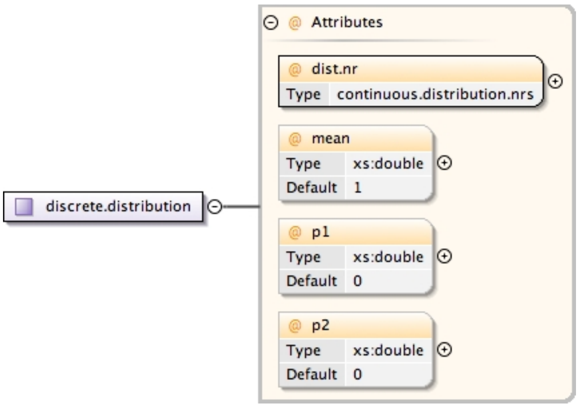

Used by	Element	skin-snip.variability

Attributes

QName	Type	Default	Use	
**dist.nr**	continuous.distribution.nrs		required	
**mean**	xs:double	1	optional	
**p1**	xs:double	0	optional	
**p2**	xs:double	0	optional	

Source

```
<xs:complexType name="discrete.distribution">
  <xs:attribute name="dist.nr" use="required" type="continuous.distribution.nrs"/>
  <xs:attribute name="mean" use="optional" type="xs:double" default="1"/>
  <xs:attribute name="p1" use="optional" type="xs:double" default="0"/>
  <xs:attribute name="p2" use="optional" type="xs:double" default="0"/>
</xs:complexType>
```

Complex Type `ym`

Namespace	No namespace

Diagram

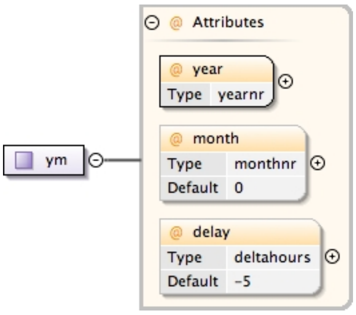

Used by

Elements	extra.surveys/survey, periodic.surveys/start, periodic.surveys/stop

Attributes

QName	Type	Default	Use	
**delay**	deltahours	-5	optional	
**month**	monthnr	0	optional	
**year**	yearnr		required	

Source

```
<xs:complexType name="ym">
  <xs:attribute name="year" use="required" type="yearnr"/>
  <xs:attribute name="month" use="optional" type="monthnr" default="0"/>
  <xs:attribute name="delay" use="optional" type="deltahours" default="-5"/>
</xs:complexType>
```

Complex Type `intervalym`

Namespace: No namespace

Diagram

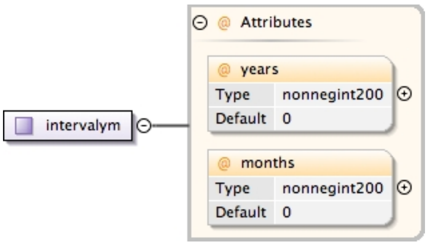

Used by

Element	periodic.surveys/interval

Attributes

QName	Type	Default	Use	
**months**	nonnegint200	0	optional	
**years**	nonnegint200	0	optional	

Source

```
<xs:complexType name="intervalym">
  <xs:attribute name="years" use="optional" type="nonnegint200" default="0"/>
  <xs:attribute name="months" use="optional" type="nonnegint200" default="0"/>
</xs:complexType>
```

Complex Type `environment.definition`

Namespace: No namespace

Diagram

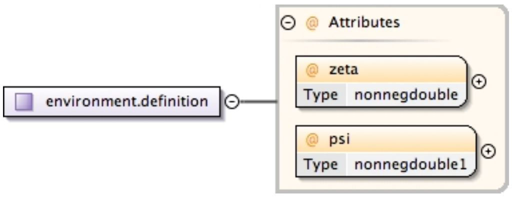

Used by

Element	exposure.and.contribution/environment

Attributes

QName	Type	Use	
**psi**	nonnegdouble1	required	
**zeta**	nonnegdouble	required	

Source

```
<xs:complexType name="environment.definition">
  <xs:attribute name="zeta" use="required" type="nonnegdouble"/>
```

```
  <xs:attribute name="psi" use="required" type="nonnegdouble1"/>
</xs:complexType>
```

Complex Type `age.dependency.table`

Namespace	No namespace
Diagram	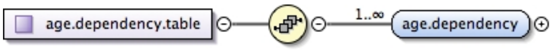
Used by	Elements: female/age.dependent.contribution, female/age.dependent.exposure, male/age.dependent.contribution, male/age.dependent.exposure
Model	age.dependency+
Children	age.dependency

Source:

```
<xs:complexType name="age.dependency.table">
  <xs:sequence>
    <xs:element name="age.dependency" maxOccurs="unbounded">
      <xs:complexType>
        <xs:attribute name="age" use="required" type="nonnegdouble"/>
        <xs:attribute name="x" use="required" type="nonnegdouble"/>
      </xs:complexType>
    </xs:element>
  </xs:sequence>
</xs:complexType>
```

Simple Type(s)

Simple Type `nonnegint200`

Namespace	No namespace
Diagram	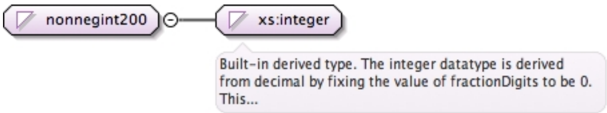
Type	restriction of xs:integer
Facets	maxInclusive: `200`<br>minInclusive: `0`
Used by	Attributes: age.and.sex.specific.compliance/@age.limit, age.class/@age.limit, age.group/@age.limit, fertility/@age.limit, intervalym/@months, intervalym/@years, surveillance/@nr.skin-snips, survival/@age.limit

Source:

```
<xs:simpleType name="nonnegint200">
  <xs:restriction base="xs:integer">
    <xs:minInclusive value="0"/>
    <xs:maxInclusive value="200"/>
  </xs:restriction>
</xs:simpleType>
```

Simple Type `nonnegativeinteger`

Namespace	No namespace
Diagram	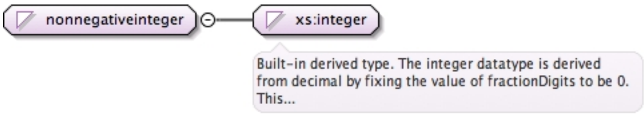
Type	restriction of xs:integer
Facets	maxInclusive: `2000000000`<br>minInclusive: `0`
Used by	Attributes: age.group/@n.females, age.group/@n.males, the.reaper/@max.population.size, worm/@mf-lifespan

Source:

```
<xs:simpleType name="nonnegativeinteger">
  <xs:restriction base="xs:integer">
    <xs:minInclusive value="0"/>
    <xs:maxInclusive value="2000000000"/>
  </xs:restriction>
</xs:simpleType>
```

Simple Type function.nrs

Namespace	No namespace
Diagram	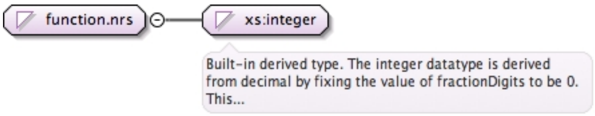
Type	restriction of xs:integer
Facets	maxInclusive 5<br>minInclusive 0
Used by	Attribute functional.relationship/@fun.nr
Source	<xs:simpleType name="function.nrs"><br><xs:restriction base="xs:integer"><br><xs:minInclusive value="0"/><br><xs:maxInclusive value="5"/><br></xs:restriction><br></xs:simpleType>

Simple Type continuous.distribution.nrs

Namespace	No namespace
Diagram	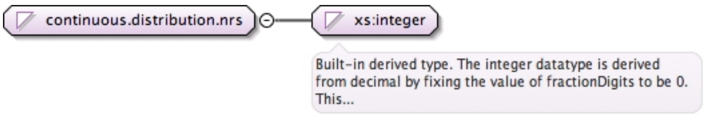
Type	restriction of xs:integer
Facets	maxInclusive 7<br>minInclusive 0
Used by	Attributes continuous.distribution/@dist.nr, discrete.distribution/@dist.nr
Source	<xs:simpleType name="continuous.distribution.nrs"><br><xs:restriction base="xs:integer"><br><xs:minInclusive value="0"/><br><xs:maxInclusive value="7"/><br></xs:restriction><br></xs:simpleType>

Simple Type discrete.distribution.nrs

Namespace	No namespace
Diagram	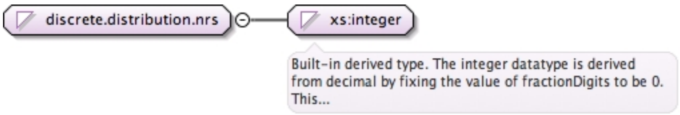
Type	restriction of xs:integer
Facets	maxInclusive 6<br>minInclusive 0
Source	<xs:simpleType name="discrete.distribution.nrs"><br><xs:restriction base="xs:integer"><br><xs:minInclusive value="0"/><br><xs:maxInclusive value="6"/><br></xs:restriction><br></xs:simpleType>

Simple Type deltahours

Namespace	No namespace
Diagram	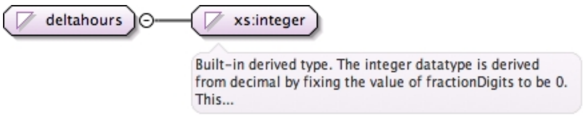

Type	restriction of xs:integer	
Facets	maxInclusive	12
	minInclusive	-12
Used by	Attributes	fertility.table/@delay, the.reaper/@delay, treatment.round/@delay, worm/@monthly.event.delay, ym/@delay

Source

```
<xs:simpleType name="deltahours">
  <xs:restriction base="xs:integer">
    <xs:minInclusive value="-12"/>
    <xs:maxInclusive value="12"/>
  </xs:restriction>
</xs:simpleType>
```

Simple Type monthnr

Namespace	No namespace	
Diagram	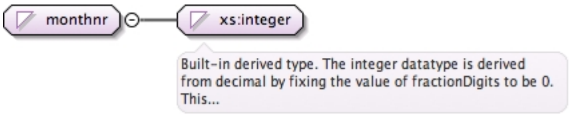	
Type	restriction of xs:integer	
Facets	maxInclusive	12
	minInclusive	0
Used by	Attributes	mbr/@month, period/@start.month, period/@stop.month, treatment.round/@month, ym/@month

Source

```
<xs:simpleType name="monthnr">
  <xs:restriction base="xs:integer">
    <xs:minInclusive value="0"/>
    <xs:maxInclusive value="12"/>
  </xs:restriction>
</xs:simpleType>
```

Simple Type yearnr

Namespace	No namespace	
Diagram	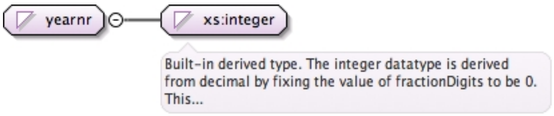	
Type	restriction of xs:integer	
Facets	maxInclusive	2200
	minInclusive	1700
Used by	Attributes	period/@start.year, period/@stop.year, simulation/@start.year, treatment.round/@year, ym/@year

Source

```
<xs:simpleType name="yearnr">
  <xs:restriction base="xs:integer">
    <xs:minInclusive value="1700"/>
    <xs:maxInclusive value="2200"/>
  </xs:restriction>
</xs:simpleType>
```

Simple Type modeltype

Namespace	No namespace	
Diagram	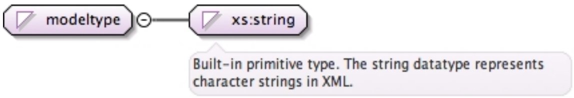	
Type	restriction of xs:string	
Facets	enumeration	onchosim
	enumeration	schistosim
	enumeration	lymfasim

Used by	Attribute	wormsim.inputfile/@model

Source

```
<xs:simpleType name="modeltype">
  <xs:restriction base="xs:string">
    <xs:enumeration value="onchosim"/>
    <xs:enumeration value="schistosim"/>
    <xs:enumeration value="lymfasim"/>
  </xs:restriction>
</xs:simpleType>
```

Simple Type nonnegdouble1

Namespace	No namespace	
Diagram	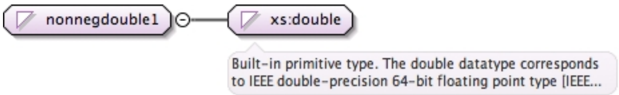	
Type	restriction of xs:double	
Facets	maxInclusive	1.0
	minInclusive	0.0
Used by	Attributes	age.and.sex.specific.compliance/@female.compliance, age.and.sex.specific.compliance/@male.compliance, compliance/@fraction.excluded, compliance/@fraction.malabsorption, environment.definition/@psi, fertility.table/@fraction.male.newborns, fertility/@birth.rate, monthly.birth.rates/@relative.birth.rate, monthly.biting.rates/@relative.biting.rate, period/@effectivity, survival/@female.survival, survival/@male.survival, the.reaper/@reap, treatment.effects/@fraction.killed, treatment.effects/@permanent.reduction.mf-production, treatment.round/@coverage

Source

```
<xs:simpleType name="nonnegdouble1">
  <xs:restriction base="xs:double">
    <xs:minInclusive value="0.0"/>
    <xs:maxInclusive value="1.0"/>
  </xs:restriction>
</xs:simpleType>
```

Simple Type nonnegdouble

Namespace	No namespace	
Diagram	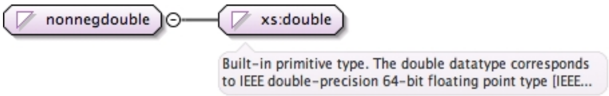	
Type	restriction of xs:double	
Facets	maxInclusive	1e100
	minInclusive	0.0
Used by	Attributes	age.dependency.table/age.dependency/@age, age.dependency.table/age.dependency/@x, age.dependent.mf-production/@labda, environment.definition/@zeta, fly/@transmission.probability, immunity/female/@alpha, immunity/female/@beta, immunity/male/@alpha, immunity/male/@beta, initial.foi/@duration, initial.foi/@foi, mating/@cycle, mating/@sex.ratio, mbr/@rate, mf-production/@age.limit, mf-production/@production, snail-population/Y0/@initial, snail-population/Y0/@mu, snail-population/Y1/@initial, snail-population/Y1/@mu, snail-population/Y1/@sigma, snail-population/Y2/@initial, snail-population/Y2/@mu, snails/@transmission.probability, treatment.effects/@period.of.recovery, treatment.effects/@shape.parameter.recovery.function

Source

```
<xs:simpleType name="nonnegdouble">
  <xs:restriction base="xs:double">
    <xs:minInclusive value="0.0"/>
    <xs:maxInclusive value="1e100"/>
  </xs:restriction>
</xs:simpleType>
```

Simple Type covmodel

Namespace	No namespace
Diagram	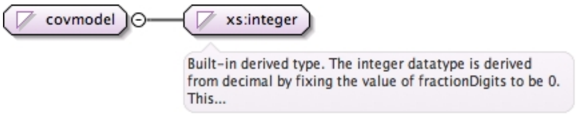

Type	restriction of xs:integer
Facets	maxInclusive 2<br>minInclusive 0
Used by	Attribute compliance/@compliance.model
Source	`<xs:simpleType name="covmodel"> <xs:restriction base="xs:integer"> <xs:minInclusive value="0"/> <xs:maxInclusive value="2"/> </xs:restriction> </xs:simpleType>`

Attribute(s)

Attribute age.class / @age.limit

Namespace	No namespace
Type	nonnegint200
Properties	use: required
Facets	maxInclusive 200<br>minInclusive 0
Used by	Complex Type age.class
Source	`<xs:attribute name="age.limit" use="required" type="nonnegint200"/>`

Attribute age.group / @age.limit

Namespace	No namespace
Type	nonnegint200
Properties	use: required
Facets	maxInclusive 200<br>minInclusive 0
Used by	Complex Type age.group
Source	`<xs:attribute name="age.limit" use="required" type="nonnegint200"/>`

Attribute age.group / @n.males

Namespace	No namespace
Type	nonnegativeinteger
Properties	use: required
Facets	maxInclusive 2000000000<br>minInclusive 0
Used by	Complex Type age.group
Source	`<xs:attribute name="n.males" use="required" type="nonnegativeinteger"/>`

Attribute age.group / @n.females

Namespace	No namespace
Type	nonnegativeinteger
Properties	use: required
Facets	maxInclusive 2000000000<br>minInclusive 0
Used by	Complex Type age.group

Source	<xs:attribute name="n.females" use="required" type="nonnegativeinteger"/>	

Attribute functional.relationship / @fun.nr

Namespace	No namespace	
Type	function.nrs	
Properties	use:	required
Facets	maxInclusive	5
	minInclusive	0
Used by	Complex Type	functional.relationship
Source	<xs:attribute name="fun.nr" use="required" type="function.nrs"/>	

Attribute functional.relationship / @a

Namespace	No namespace	
Type	xs:double	
Properties	use:	required
Used by	Complex Type	functional.relationship
Source	<xs:attribute name="a" use="required" type="xs:double"/>	

Attribute functional.relationship / @b

Namespace	No namespace	
Type	xs:double	
Properties	use:	optional
	default:	0
Used by	Complex Type	functional.relationship
Source	<xs:attribute name="b" use="optional" type="xs:double" default="0"/>	

Attribute functional.relationship / @c

Namespace	No namespace	
Type	xs:double	
Properties	use:	optional
	default:	0
Used by	Complex Type	functional.relationship
Source	<xs:attribute name="c" use="optional" type="xs:double" default="0"/>	

Attribute continuous.distribution / @dist.nr

Namespace	No namespace	
Type	continuous.distribution.nrs	
Properties	use:	required
Facets	maxInclusive	7
	minInclusive	0
Used by	Complex Type	continuous.distribution
Source	<xs:attribute name="dist.nr" use="required" type="continuous.distribution.nrs"/>	

Attribute continuous.distribution / @offset

Namespace	No namespace

Type	xs:double	
Properties	use:	optional
	default:	0
Used by	Complex Type	continuous.distribution
Source	`<xs:attribute name="offset" use="optional" type="xs:double" default="0"/>`	

Attribute continuous.distribution / @min

Namespace	No namespace	
Type	xs:double	
Properties	use:	optional
	default:	-1e6
Used by	Complex Type	continuous.distribution
Source	`<xs:attribute name="min" use="optional" type="xs:double" default="-1e6"/>`	

Attribute continuous.distribution / @max

Namespace	No namespace	
Type	xs:double	
Properties	use:	optional
	default:	1e6
Used by	Complex Type	continuous.distribution
Source	`<xs:attribute name="max" use="optional" type="xs:double" default="1e6"/>`	

Attribute continuous.distribution / @mean

Namespace	No namespace	
Type	xs:double	
Properties	use:	optional
	default:	1
Used by	Complex Type	continuous.distribution
Source	`<xs:attribute name="mean" use="optional" type="xs:double" default="1"/>`	

Attribute continuous.distribution / @p1

Namespace	No namespace	
Type	xs:double	
Properties	use:	optional
	default:	0
Used by	Complex Type	continuous.distribution
Source	`<xs:attribute name="p1" use="optional" type="xs:double" default="0"/>`	

Attribute continuous.distribution / @p2

Namespace	No namespace	
Type	xs:double	
Properties	use:	optional
	default:	0
Used by	Complex Type	continuous.distribution
Source	`<xs:attribute name="p2" use="optional" type="xs:double" default="0"/>`	

Attribute discrete.distribution / @dist.nr

Namespace	No namespace	
Type	continuous.distribution.nrs	
Properties	use:	required
Facets	maxInclusive	7
	minInclusive	0
Used by	Complex Type	discrete.distribution
Source	`<xs:attribute name="dist.nr" use="required" type="continuous.distribution.nrs"/>`	

Attribute discrete.distribution / @mean

Namespace	No namespace	
Type	xs:double	
Properties	use:	optional
	default:	1
Used by	Complex Type	discrete.distribution
Source	`<xs:attribute name="mean" use="optional" type="xs:double" default="1"/>`	

Attribute discrete.distribution / @p1

Namespace	No namespace	
Type	xs:double	
Properties	use:	optional
	default:	0
Used by	Complex Type	discrete.distribution
Source	`<xs:attribute name="p1" use="optional" type="xs:double" default="0"/>`	

Attribute discrete.distribution / @p2

Namespace	No namespace	
Type	xs:double	
Properties	use:	optional
	default:	0
Used by	Complex Type	discrete.distribution
Source	`<xs:attribute name="p2" use="optional" type="xs:double" default="0"/>`	

Attribute ym / @year

Namespace	No namespace	
Type	yearnr	
Properties	use:	required
Facets	maxInclusive	2200
	minInclusive	1700
Used by	Complex Type	ym
Source	`<xs:attribute name="year" use="required" type="yearnr"/>`	

Attribute ym / @month

Namespace	No namespace
Type	monthnr

Properties	use:	optional
	default:	0
Facets	maxInclusive	12
	minInclusive	0
Used by	Complex Type	ym
Source	`<xs:attribute name="month" use="optional" type="monthnr" default="0"/>`	

Attribute ym / @delay

Namespace	No namespace	
Type	deltahours	
Properties	use:	optional
	default:	-5
Facets	maxInclusive	12
	minInclusive	-12
Used by	Complex Type	ym
Source	`<xs:attribute name="delay" use="optional" type="deltahours" default="-5"/>`	

Attribute intervalym / @years

Namespace	No namespace	
Type	nonnegint200	
Properties	use:	optional
	default:	0
Facets	maxInclusive	200
	minInclusive	0
Used by	Complex Type	intervalym
Source	`<xs:attribute name="years" use="optional" type="nonnegint200" default="0"/>`	

Attribute intervalym / @months

Namespace	No namespace	
Type	nonnegint200	
Properties	use:	optional
	default:	0
Facets	maxInclusive	200
	minInclusive	0
Used by	Complex Type	intervalym
Source	`<xs:attribute name="months" use="optional" type="nonnegint200" default="0"/>`	

Attribute surveillance / @nr.skin-snips

Namespace	No namespace	
Type	nonnegint200	
Properties	use:	required
Facets	maxInclusive	200
	minInclusive	0
Used by	Element	surveillance
Source	`<xs:attribute name="nr.skin-snips" use="required" type="nonnegint200"/>`	

Attribute surveillance / @skin-snip.categories

Namespace	No namespace	
Type	xs:string	
Properties	default:	0.5,1,2,4,8,16,32,64,128,256,512,1e6
Used by	Element	surveillance
Source	`<xs:attribute name="skin-snip.categories" default="0.5,1,2,4,8,16,32,64,128,256,512,1e6" type="xs:string"/>`	

Attribute simulation / @start.year

Namespace	No namespace	
Type	yearnr	
Properties	default:	1875
Facets	maxInclusive	2200
	minInclusive	1700
Used by	Element	simulation
Source	`<xs:attribute name="start.year" default="1875" type="yearnr"/>`	

Attribute the.reaper / @max.population.size

Namespace	No namespace	
Type	nonnegativeinteger	
Properties	use:	optional
	default:	440
Facets	maxInclusive	2000000000
	minInclusive	0
Used by	Element	the.reaper
Source	`<xs:attribute name="max.population.size" use="optional" type="nonnegativeinteger" default="440"/>`	

Attribute the.reaper / @reap

Namespace	No namespace	
Type	nonnegdouble1	
Properties	use:	optional
	default:	0.1
Facets	maxInclusive	1.0
	minInclusive	0.0
Used by	Element	the.reaper
Source	`<xs:attribute name="reap" use="optional" type="nonnegdouble1" default="0.1 "/>`	

Attribute the.reaper / @delay

Namespace	No namespace	
Type	deltahours	
Properties	use:	optional
	default:	-2
Facets	maxInclusive	12
	minInclusive	-12
Used by	Element	the.reaper

Source	`<xs:attribute name="delay" use="optional" type="deltahours" default="-2"/>`

Attribute survival / @age.limit

Namespace	No namespace	
Type	nonnegint200	
Properties	use:	required
Facets	maxInclusive	200
	minInclusive	0
Used by	Element	survival
Source	`<xs:attribute name="age.limit" use="required" type="nonnegint200"/>`	

Attribute survival / @male.survival

Namespace	No namespace	
Type	nonnegdouble1	
Properties	use:	required
Facets	maxInclusive	1.0
	minInclusive	0.0
Used by	Element	survival
Source	`<xs:attribute name="male.survival" use="required" type="nonnegdouble1"/>`	

Attribute survival / @female.survival

Namespace	No namespace	
Type	nonnegdouble1	
Properties	use:	required
Facets	maxInclusive	1.0
	minInclusive	0.0
Used by	Element	survival
Source	`<xs:attribute name="female.survival" use="required" type="nonnegdouble1"/>`	

Attribute fertility / @age.limit

Namespace	No namespace	
Type	nonnegint200	
Properties	use:	required
Facets	maxInclusive	200
	minInclusive	0
Used by	Element	fertility
Source	`<xs:attribute name="age.limit" use="required" type="nonnegint200"/>`	

Attribute fertility / @birth.rate

Namespace	No namespace	
Type	nonnegdouble1	
Properties	use:	required
Facets	maxInclusive	1.0
	minInclusive	0.0
Used by	Element	fertility

Source	`<xs:attribute name="birth.rate" use="required" type="nonnegdouble1"/>`

Attribute fertility.table / @fraction.male.newborns

Namespace	No namespace	
Type	nonnegdouble1	
Properties	default:	0.50
Facets	maxInclusive	1.0
	minInclusive	0.0
Used by	Element	fertility.table
Source	`<xs:attribute name="fraction.male.newborns" type="nonnegdouble1" default="0.50"/>`	

Attribute fertility.table / @delay

Namespace	No namespace	
Type	deltahours	
Properties	default:	-3
Facets	maxInclusive	12
	minInclusive	-12
Used by	Element	fertility.table
Source	`<xs:attribute name="delay" type="deltahours" default="-3"/>`	

Attribute environment.definition / @zeta

Namespace	No namespace	
Type	nonnegdouble	
Properties	use:	required
Facets	maxInclusive	1e100
	minInclusive	0.0
Used by	Complex Type	environment.definition
Source	`<xs:attribute name="zeta" use="required" type="nonnegdouble"/>`	

Attribute environment.definition / @psi

Namespace	No namespace	
Type	nonnegdouble1	
Properties	use:	required
Facets	maxInclusive	1.0
	minInclusive	0.0
Used by	Complex Type	environment.definition
Source	`<xs:attribute name="psi" use="required" type="nonnegdouble1"/>`	

Attribute initial.foi / @duration

Namespace	No namespace	
Type	nonnegdouble	
Properties	use:	required
Facets	maxInclusive	1e100
	minInclusive	0.0
Used by	Element	initial.foi

Source	`<xs:attribute name="duration" use="required" type="nonnegdouble"/>`

Attribute initial.foi / @foi

Namespace	No namespace	
Type	nonnegdouble	
Properties	use:	required
Facets	maxInclusive	1e100
	minInclusive	0.0
Used by	Element	initial.foi
Source	`<xs:attribute name="foi" use="required" type="nonnegdouble"/>`	

Attribute age.dependency.table / age.dependency / @age

Namespace	No namespace	
Type	nonnegdouble	
Properties	use:	required
Facets	maxInclusive	1e100
	minInclusive	0.0
Used by	Element	age.dependency.table/age.dependency
Source	`<xs:attribute name="age" use="required" type="nonnegdouble"/>`	

Attribute age.dependency.table / age.dependency / @x

Namespace	No namespace	
Type	nonnegdouble	
Properties	use:	required
Facets	maxInclusive	1e100
	minInclusive	0.0
Used by	Element	age.dependency.table/age.dependency
Source	`<xs:attribute name="x" use="required" type="nonnegdouble"/>`	

Attribute immunity / male / @alpha

Namespace	No namespace	
Type	nonnegdouble	
Properties	use:	required
Facets	maxInclusive	1e100
	minInclusive	0.0
Used by	Element	immunity/male
Source	`<xs:attribute name="alpha" use="required" type="nonnegdouble"/>`	

Attribute immunity / male / @beta

Namespace	No namespace	
Type	nonnegdouble	
Properties	use:	required
Facets	maxInclusive	1e100
	minInclusive	0.0
Used by	Element	immunity/male

Source	`<xs:attribute name="beta" use="required" type="nonnegdouble"/>`

Attribute immunity / female / @alpha

Namespace	No namespace
Type	nonnegdouble
Properties	use: required
Facets	maxInclusive 1e100<br>minInclusive 0.0
Used by	Element immunity/female
Source	`<xs:attribute name="alpha" use="required" type="nonnegdouble"/>`

Attribute immunity / female / @beta

Namespace	No namespace
Type	nonnegdouble
Properties	use: required
Facets	maxInclusive 1e100<br>minInclusive 0.0
Used by	Element immunity/female
Source	`<xs:attribute name="beta" use="required" type="nonnegdouble"/>`

Attribute mating / @sex.ratio

Namespace	No namespace
Type	nonnegdouble
Properties	use: optional<br>default: 1
Facets	maxInclusive 1e100<br>minInclusive 0.0
Used by	Element mating
Source	`<xs:attribute name="sex.ratio" use="optional" type="nonnegdouble" default="1"/>`

Attribute mating / @cycle

Namespace	No namespace
Type	nonnegdouble
Properties	use: required
Facets	maxInclusive 1e100<br>minInclusive 0.0
Used by	Element mating
Source	`<xs:attribute name="cycle" use="required" type="nonnegdouble"/>`

Attribute mating / @male.potential

Namespace	No namespace
Type	xs:double
Properties	use: required
Used by	Element mating
Source	`<xs:attribute name="male.potential" use="required" type="xs:double"/>`

Attribute mf-production / @age.limit

Namespace	No namespace	
Type	nonnegdouble	
Properties	use:	required
Facets	maxInclusive	1e100
	minInclusive	0.0
Used by	Element	mf-production
Source	`<xs:attribute name="age.limit" use="required" type="nonnegdouble"/>`	

Attribute mf-production / @production

Namespace	No namespace	
Type	nonnegdouble	
Properties	use:	required
Facets	maxInclusive	1e100
	minInclusive	0.0
Used by	Element	mf-production
Source	`<xs:attribute name="production" use="required" type="nonnegdouble"/>`	

Attribute age.dependent.mf-production / @labda

Namespace	No namespace	
Type	nonnegdouble	
Properties	default:	0.0
Facets	maxInclusive	1e100
	minInclusive	0.0
Used by	Element	age.dependent.mf-production
Source	`<xs:attribute name="labda" default="0.0" type="nonnegdouble"/>`	

Attribute worm / @mf-lifespan

Namespace	No namespace	
Type	nonnegativeinteger	
Properties	use:	optional
	default:	9
Facets	maxInclusive	2000000000
	minInclusive	0
Used by	Element	worm
Source	`<xs:attribute name="mf-lifespan" use="optional" type="nonnegativeinteger" default="9"/>`	

Attribute worm / @monthly.event.delay

Namespace	No namespace	
Type	deltahours	
Properties	use:	optional
	default:	-1
Facets	maxInclusive	12
	minInclusive	-12
Used by	Element	worm

Source	`<xs:attribute name="monthly.event.delay" use="optional" type="deltahours" default="-1"/>`

Attribute mbr / @month

Namespace	No namespace	
Type	monthnr	
Properties	use:	required
Facets	maxInclusive	12
	minInclusive	0
Used by	Element	mbr
Source	`<xs:attribute name="month" use="required" type="monthnr"/>`	

Attribute mbr / @rate

Namespace	No namespace	
Type	nonnegdouble	
Properties	use:	required
Facets	maxInclusive	1e100
	minInclusive	0.0
Used by	Element	mbr
Source	`<xs:attribute name="rate" use="required" type="nonnegdouble"/>`	

Attribute monthly.biting.rates / @relative.biting.rate

Namespace	No namespace	
Type	nonnegdouble1	
Properties	use:	required
Facets	maxInclusive	1.0
	minInclusive	0.0
Used by	Element	monthly.biting.rates
Source	`<xs:attribute name="relative.biting.rate" use="required" type="nonnegdouble1"/>`	

Attribute fly / @transmission.probability

Namespace	No namespace	
Type	nonnegdouble	
Properties	use:	required
Facets	maxInclusive	1e100
	minInclusive	0.0
Used by	Element	fly
Source	`<xs:attribute name="transmission.probability" use="required" type="nonnegdouble"/>`	

Attribute monthly.birth.rates / @relative.birth.rate

Namespace	No namespace	
Type	nonnegdouble1	
Properties	use:	required
Facets	maxInclusive	1.0
	minInclusive	0.0
Used by	Element	monthly.birth.rates

Source	<xs:attribute name="relative.birth.rate" use="required" type="nonnegdouble1"/>

Attribute snail-population / Y0 / @initial

Namespace	No namespace	
Type	nonnegdouble	
Properties	use:	required
Facets	maxInclusive	1e100
	minInclusive	0.0
Used by	Element	snail-population/Y0
Source	<xs:attribute name="initial" use="required" type="nonnegdouble"/>	

Attribute snail-population / Y0 / @mu

Namespace	No namespace	
Type	nonnegdouble	
Properties	use:	required
Facets	maxInclusive	1e100
	minInclusive	0.0
Used by	Element	snail-population/Y0
Source	<xs:attribute name="mu" use="required" type="nonnegdouble"/>	

Attribute snail-population / Y1 / @initial

Namespace	No namespace	
Type	nonnegdouble	
Properties	use:	required
Facets	maxInclusive	1e100
	minInclusive	0.0
Used by	Element	snail-population/Y1
Source	<xs:attribute name="initial" use="required" type="nonnegdouble"/>	

Attribute snail-population / Y1 / @mu

Namespace	No namespace	
Type	nonnegdouble	
Properties	use:	required
Facets	maxInclusive	1e100
	minInclusive	0.0
Used by	Element	snail-population/Y1
Source	<xs:attribute name="mu" use="required" type="nonnegdouble"/>	

Attribute snail-population / Y1 / @sigma

Namespace	No namespace	
Type	nonnegdouble	
Properties	use:	required
Facets	maxInclusive	1e100
	minInclusive	0.0
Used by	Element	snail-population/Y1

Source	<xs:attribute name="sigma" use="required" type="nonnegdouble"/>

Attribute snail-population / Y2 / @initial

Namespace	No namespace	
Type	nonnegdouble	
Properties	use:	required
Facets	maxInclusive	1e100
	minInclusive	0.0
Used by	Element	snail-population/Y2
Source	<xs:attribute name="initial" use="required" type="nonnegdouble"/>	

Attribute snail-population / Y2 / @mu

Namespace	No namespace	
Type	nonnegdouble	
Properties	use:	required
Facets	maxInclusive	1e100
	minInclusive	0.0
Used by	Element	snail-population/Y2
Source	<xs:attribute name="mu" use="required" type="nonnegdouble"/>	

Attribute snails / @transmission.probability

Namespace	No namespace	
Type	nonnegdouble	
Properties	use:	required
Facets	maxInclusive	1e100
	minInclusive	0.0
Used by	Element	snails
Source	<xs:attribute name="transmission.probability" use="required" type="nonnegdouble"/>	

Attribute treatment.round / @year

Namespace	No namespace	
Type	yearnr	
Properties	use:	required
Facets	maxInclusive	2200
	minInclusive	1700
Used by	Element	treatment.round
Source	<xs:attribute name="year" use="required" type="yearnr"/>	

Attribute treatment.round / @month

Namespace	No namespace	
Type	monthnr	
Properties	use:	required
Facets	maxInclusive	12
	minInclusive	0
Used by	Element	treatment.round

Source	`<xs:attribute name="month" use="required" type="monthnr"/>`

Attribute treatment.round / @delay

Namespace	No namespace	
Type	deltahours	
Properties	use:	optional
	default:	-4
Facets	maxInclusive	12
	minInclusive	-12
Used by	Element	treatment.round
Source	`<xs:attribute name="delay" use="optional" type="deltahours" default="-4"/>`	

Attribute treatment.round / @coverage

Namespace	No namespace	
Type	nonnegdouble1	
Properties	use:	required
Facets	maxInclusive	1.0
	minInclusive	0.0
Used by	Element	treatment.round
Source	`<xs:attribute name="coverage" use="required" type="nonnegdouble1"/>`	

Attribute age.and.sex.specific.compliance / @age.limit

Namespace	No namespace	
Type	nonnegint200	
Properties	use:	required
Facets	maxInclusive	200
	minInclusive	0
Used by	Element	age.and.sex.specific.compliance
Source	`<xs:attribute name="age.limit" use="required" type="nonnegint200"/>`	

Attribute age.and.sex.specific.compliance / @male.compliance

Namespace	No namespace	
Type	nonnegdouble1	
Properties	use:	required
Facets	maxInclusive	1.0
	minInclusive	0.0
Used by	Element	age.and.sex.specific.compliance
Source	`<xs:attribute name="male.compliance" use="required" type="nonnegdouble1"/>`	

Attribute age.and.sex.specific.compliance / @female.compliance

Namespace	No namespace	
Type	nonnegdouble1	
Properties	use:	required
Facets	maxInclusive	1.0
	minInclusive	0.0

Used by	Element age.and.sex.specific.compliance
Source	`<xs:attribute name="female.compliance" use="required" type="nonnegdouble1"/>`

Attribute compliance / @fraction.excluded

Namespace	No namespace	
Type	nonnegdouble1	
Properties	use:	required
Facets	maxInclusive	1.0
	minInclusive	0.0
Used by	Element	compliance
Source	`<xs:attribute name="fraction.excluded" use="required" type="nonnegdouble1"/>`	

Attribute compliance / @fraction.malabsorption

Namespace	No namespace	
Type	nonnegdouble1	
Properties	use:	required
Facets	maxInclusive	1.0
	minInclusive	0.0
Used by	Element	compliance
Source	`<xs:attribute name="fraction.malabsorption" use="required" type="nonnegdouble1"/>`	

Attribute compliance / @compliance.model

Namespace	No namespace	
Type	covmodel	
Properties	default:	0
Facets	maxInclusive	2
	minInclusive	0
Used by	Element	compliance
Source	`<xs:attribute name="compliance.model" default="0" type="covmodel"/>`	

Attribute compliance / @test.first

Namespace	No namespace	
Type	xs:boolean	
Properties	default:	false
Used by	Element	compliance
Source	`<xs:attribute name="test.first" default="false" type="xs:boolean"/>`	

Attribute treatment.effects / @permanent.reduction.mf-production

Namespace	No namespace	
Type	nonnegdouble1	
Properties	use:	required
Facets	maxInclusive	1.0
	minInclusive	0.0
Used by	Element	treatment.effects
Source	`<xs:attribute name="permanent.reduction.mf-production" use="required" type="nonnegdouble1"/>`	

Attribute `treatment.effects / @period.of.recovery`

Namespace	No namespace	
Type	nonnegdouble	
Properties	use:	`required`
Facets	maxInclusive	`1e100`
	minInclusive	`0.0`
Used by	Element	treatment.effects
Source	`<xs:attribute name="period.of.recovery" use="required" type="nonnegdouble"/>`	

Attribute `treatment.effects / @shape.parameter.recovery.function`

Namespace	No namespace	
Type	nonnegdouble	
Properties	use:	`required`
Facets	maxInclusive	`1e100`
	minInclusive	`0.0`
Used by	Element	treatment.effects
Source	`<xs:attribute name="shape.parameter.recovery.function" use="required" type="nonnegdouble"/>`	

Attribute `treatment.effects / @fraction.killed`

Namespace	No namespace	
Type	nonnegdouble1	
Properties	use:	`required`
Facets	maxInclusive	`1.0`
	minInclusive	`0.0`
Used by	Element	treatment.effects
Source	`<xs:attribute name="fraction.killed" use="required" type="nonnegdouble1"/>`	

Attribute `period / @start.year`

Namespace	No namespace	
Type	yearnr	
Properties	use:	`required`
Facets	maxInclusive	`2200`
	minInclusive	`1700`
Used by	Element	period
Source	`<xs:attribute name="start.year" use="required" type="yearnr"/>`	

Attribute `period / @start.month`

Namespace	No namespace	
Type	monthnr	
Properties	default:	`0`
Facets	maxInclusive	`12`
	minInclusive	`0`
Used by	Element	period
Source	`<xs:attribute name="start.month" default="0" type="monthnr"/>`	

Attribute period / @stop.year

Namespace	No namespace
Type	yearnr
Properties	use: required
Facets	maxInclusive 2200<br>minInclusive 1700
Used by	Element period
Source	`<xs:attribute name="stop.year" use="required" type="yearnr"/>`

Attribute period / @stop.month

Namespace	No namespace
Type	monthnr
Properties	default: 0
Facets	maxInclusive 12<br>minInclusive 0
Used by	Element period
Source	`<xs:attribute name="stop.month" default="0" type="monthnr"/>`

Attribute period / @effectivity

Namespace	No namespace
Type	nonnegdouble1
Properties	use: required
Facets	maxInclusive 1.0<br>minInclusive 0.0
Used by	Element period
Source	`<xs:attribute name="effectivity" use="required" type="nonnegdouble1"/>`

Attribute wormsim.inputfile / @model

Namespace	No namespace
Type	modeltype
Properties	use: required
Facets	enumeration onchosim<br>enumeration schistosim<br>enumeration lymfasim
Used by	Element wormsim.inputfile
Source	`<xs:attribute name="model" type="modeltype" use="required"/>`
