# Supplementary material for: Required duration of mass ivermectin treatment for onchocerciasis elimination in Africa: a comparative modelling analysis
Source: Parasit Vectors. 2015 Oct 22;8:552. doi: 10.1186/s13071-015-1159-9 (PMC4618738; doi:10.1186/s13071-015-1159-9)
Supplement: Additional file 3: — A Word document containing instructions for installing and running EPIONCHO (Instructions for installing & running EPIONCHO.docx). (DOCX 71 kb) [file 13071_2015_1159_MOESM3_ESM.docx]

# Required duration of mass ivermectin treatment for onchocerciasis elimination in Africa: a comparative modelling analysis

Wilma Stolk, Martin Walker, Luc E Coffeng, María-Gloria Basáñez, Sake J de Vlas

Additional file 1

Instructions for installing and running EPIONCHO

EPIONCHO is written in C code (EPIONCHO.c) which is called from within R [1] and solved numerically using a numerical integration algorithm implemented with the deSolve package [2]. Here we give instructions for installing and running EPIONCHO.

# Getting started

The first step is to set up a new working directory for R. In Windows this could be C:/EPIONCHO or in Mac OS ~/EPIONCHO. Now place the EPIONCHO source code (EPIONCHO.c) and the R script (EPIONCHO.R) in this newly created directory. The next step is to open R, set the working directory, and install the required deSolve package.

> setwd("~/EPIONCHO")

> install.packages("deSolve")

> library("deSolve")

# Compiling EPIONCHO source code and loading the model into R

The EPIONCHO source code is complied into either a shared object .so (Mac OS/Unix) or a dynamic link library .dll (Windows). This is then loaded into R so that it can be called like any other R function.

> system("R CMD SHLIB EPIONCHO.c")

> dyn.load(paste("EPIONCHO", .Platform$dynlib.ext, sep=""))

# Loading the associated R script

The R script in file EPIONCHO.R comprises a vector of default parameter values for EPIONCHO; a function for initialising the numerical integration routine; and two functions for calling EPIONCHO and returning the desired output, either for running the model to endemic equilibrium, or for simulating an ivermectin mass drug administration (MDA) programme. The R script is run, and the parameters and functions loaded, using the source function.

> source("EPIONCHO.R")

# Default parameter values

Default parameter values for EPIONCHO are defined within the (named) R vector theta and correspond to those used in Filipe et al., 2005 [3] and Turner et al., 2013, 2014a and 2014b [4-6] with the exception of kW, the overdispersion parameter describing the distribution of adult worms, which is here assigned an arbitrarily large value in accordance with the assumption of a Poisson distribution, and the fraction of vector blood meals taken from humans, which is aligned with that used by ONCHOSIM (Table 2 in the main text), particularly for comparison of the relationship between the endemic prevalence of infection and the annual biting rate (Figure 1 in main text).

The key parameters varied to generate the results presented in this paper are: (i) the annual biting rate, ABR (the number of vector bites per person per year), to modify the intensity of transmission and the starting endemic prevalence of infection; (ii) the number of treatments, ntreat; (iii) the frequency of treatments, ftreat, whereby ftreat=1 for annual treatments with ivermectin and ftreat=0.5 for biannual treatments, and (iv) the coverage of treatment in the eligible population cov, which is internally converted into a coverage of treatment in the total population and given as a model output (and matched to the desired coverage level for the simulations). Parameter noncmp defines the fraction of the *eligible* population that *never takes treatment*, so-called the proportion of systematic non-compliers [4]. This parameter is left at its default value of 0 for the simulation results presented in this paper. The default values of ABR and cov correspond to a baseline microfilarial prevalence of 51% in people aged ≥ 5 years and a total population coverage of 65%.

> theta["kW"]

kW

999

> theta["h"]

h

0.97

> theta[35:39]

ABR ntreat ftreat cov noncmp

2275.000 5.000 1.000 0.805 0.000

# Generating initial values for numerical integration

The function init takes the parameter vector theta as its sole input and returns appropriate starting values for running a simulation. These starting values *do not* correspond to endemic equilibrium and, therefore, it is important to run the model for a suitably long initial duration before simulating an MDA programme that it assumed to start at endemic equilibrium (i.e. at the pre-control, initial epidemiological situation with a stable parasite population in humans and blackfly vectors). The parameter startTreat defines this initial duration that EPIONCHO needs to run for, and by default is set to 80 years.

head( init(theta) )

[1] 5 0 0 0 0 0

> theta["startTreat"]

startTreat

80

# Running EPIONCHO to endemic equilibrium

The differential equations that form EPIONCHO are solved numerically using a Runga Kutta 4 numerical integration algorithm implemented using the R function rk that is included in the deSolve package. Because EPIONCHO tracks the mean number of parasites exposed to different numbers of treatments with ivermectin, in different age, sex and treatment compliance groups [4], the number of differential equations can become very large and the raw model output rather unwieldy. The principal quantities of interest—the population mean number and prevalence of skin microfilariae in different host age groups—are calculated within the EPIONCHO C code and R wrapper functions are used to extract and return these quantities. The function simequib runs EPIONCHO to the time indicated by parameter startTreat, returning the quantities of interest, but *does not* simulate the proposed MDA programme (defined by parameters ntreat, ftreat, cov and noncmp).

Running EPIONCHO (to equilibrium) with the default parameter values produces an R data frame with 11 columns: time, the time in years; Mm, the mean number of microfilariae per mg of skin in the entire population (all ages); Mm5 the mean number of microfilariae per mg of skin in individuals aged ≥ 5 years; Mm20 the mean number of microfilariae per mg of skin in individuals aged ≥ 20 years; Mpr, the prevalence of microfilariae in the entire population (all ages); Mpr5, the prevalence of microfilariae in individuals aged ≥ 5 years; Mpr20, the prevalence of microfilariae the individuals aged ≥ 20 years; ATP, the annual transmission potential (the number of infective, L3 larvae to which people are exposed, per person per year); covpop, the *population* coverage of the proposed (but not run) MDA programme; cmp; the fraction of the eligible population treated every round; and semicmp, the fraction of the eligible population treated *every other* round. (A brief description of how treatment coverage and compliance are modelled in EPIONCHO is given in the main text.) The outputs covpop, cmp, semicmp are model outputs derived from the input parameter cov and noncmp and are useful to report here—despite not simulating the proposed MDA programme—because running the model to equilibrium is much faster than running a treatment programme which requires a much smaller step size for the numerical integration. Hence, one can quickly check the values of covpop, cmp, semicmp calculated from the inputted values of cov and noncmp.

> tail( round (simequib(theta), digits = 1) )

time Mm Mm5 Mm20 Mpr Mpr5 Mpr20 ATP covpop cmp semicmp

1386 79.7 13.3 16.2 22.1 0.5 0.5 0.6 104.9 0.7 0.6 0.4

1387 79.7 13.3 16.2 22.1 0.5 0.5 0.6 104.9 0.7 0.6 0.4

1388 79.8 13.3 16.2 22.1 0.5 0.5 0.6 104.9 0.7 0.6 0.4

1389 79.9 13.3 16.2 22.1 0.5 0.5 0.6 104.9 0.7 0.6 0.4

1390 79.9 13.3 16.2 22.1 0.5 0.5 0.6 104.9 0.7 0.6 0.4

1391 80.0 13.3 16.2 22.1 0.5 0.5 0.6 104.9 0.7 0.6 0.4

# Simulating a mass drug administration (MDA) programme

Simulating an ivermectin MDA programme proceeds by calling EPIONCHO twice in two consecutive steps. The first step runs the model to endemic equilibrium, temporarily storing the raw model output, which is then passed, in the second step, as initial values to a second model run that simulates the infection dynamics through rounds of treatment. This process is implemented by the function simtrt that returns, like simequib, an 11-column data frame with the output of interest. The illustrative output below corresponds to the default parameter values (stored in theta) of 50% endemic microfilarial prevalence with 5 annual treatments given at 65% population coverage.

> out <- simtrt(theta)

> plot(out[,"time"], I(out[,"Mpr5"]*100), type="l", lwd = 2,

+ ylab = "Microfilarial prevalence in 5+ (%)",

+ xlab = "time in years")

Different numbers and frequencies of treatment given at a different coverage can be modelled by changing parameters ntreat, ftreat and cov respectively (stored within theta). Different endemic settings are modelled by changing parameter ABR. For example, the final illustrative example models 10 biannual treatments given at 80% coverage to a population with an endemic microfilarial prevalence in those aged ≥ 5 years of 62%. Note that cov describes the coverage of the eligible population (aged ≥ 5 years), which to achieve a *population* coverage of 80%, is close to 100%.

> theta["ntreat"] <- 10

> theta["ftreat"] <- 0.5

> theta["cov"] <- 0.988

> theta["ABR"] <- 3375

> out <- simtrt(theta)

# References

1. R Development Core Team. R: A language and environment for statistical computing. Vienna: R Foundation for Statistical Computing, 2011.
2. Soetaert K, Petzoldt T, Setzer RW. Solving differential equations in R: package deSolve. J Stat Soft 2010; 33: 1-25.
3. Filipe JAN, Boussinesq M, Renz A, Collins RC, Vivas-Martinez S, Grillet ME, et al. Human infection patterns and heterogeneous exposure in river blindness. Proc Natl Acad Sci U S A. 2005; 102:15265-70.
4. Turner HC, Churcher TS, Walker M, Osei-Atweneboana MY, Prichard RK, Basáñez MG. Uncertainty surrounding projections of the long-term impact of ivermectin treatment on human onchocerciasis. *PLoS Negl Trop Dis* 2013; 7: e2169.
5. Turner HC, Walker M, Churcher TS, Basáñez MG. Modelling the impact of ivermectin on river blindness and its burden of morbidity and mortality in African savannah. *Parasit Vectors* 2014; 7: 241.
6. Turner HC, Walker M, Churcher TS, Osei-Atweneboana MY, Biritwum NK, Hopkins A, et al. Reaching the London Declaration on Neglected Tropical Diseases goals for onchocerciasis: an economic evaluation of increasing the frequency of ivermectin treatment in Africa. *Clin Infect Dis* 2014; 59: 923-32.
